# An illustrated checklist of the genus *Elymnias* Hübner, 1818 (Nymphalidae, Satyrinae)

**DOI:** 10.3897/zookeys.676.12579

**Published:** 2017-05-25

**Authors:** Chia-Hsuan Wei, David J. Lohman, Djunijanti Peggie, Shen-Horn Yen

**Affiliations:** 1 Department of Biological Sciences, National Sun Yat-Sen University, Kaohsiung, 80424, Taiwan; 2 Department of Biology, City College of New York, City University of New York, New York, NY, 10031, USA; 3 Ph.D. Program in Biology, Graduate Center, City University of New York, New York, NY, 10016, USA; 4 Entomology Section, National Museum of the Philippines, Manila, 1000, Philippines; 5 Division of Zoology, Research Centre for Biology-LIPI, Cibinong-Bogor, 16911, Indonesia

**Keywords:** Batesian mimicry, convergent evolution, Elymniini, polymorphism, sexual dimorphism, Southeast Asia

## Abstract

We review the genus *Elymnias* Hübner, 1818, a morphologically diverse satyrine butterfly clade involved in multifarious Batesian mimicry relationships throughout Asia and Africa. A variety of different model species are mimicked, and many *Elymnias* species are sexually dimorphic mimics, with males and females resembling different model species. We revise species and subspecies delimitations in light of an integrative taxonomic investigation using external morphology, male and female genital morphology, and a multi-locus molecular phylogeny. There is little interspecific genitalic variation among species in this group, and previous taxonomists therefore relied almost entirely on wing patterns. Our molecular phylogenetic analysis reveals several examples of polymorphism or wing pattern divergence within a single species currently classified as two or more different species. We also found examples of wing pattern convergence among disparate lineages that mimic the same widespread model species. Frequently, two or more phenotypically similar species were classified as a single species. This comprehensive checklist reviews all names associated with *Elymnias* to align its taxonomy with the evolutionary history of the group. All available information on nomenclature, type localities, repositories of type specimens, and geographical distributions is summarized, and images of adult specimens and genitalia are provided along with distribution maps of all species and selected subspecies. We identify 2 species *incertae sedis*, establish 15 monophyletic species groups (including 1 species unplaced in any species group), and make 49 taxonomic changes, including 35 new synonyms, 7 new combinations (2 of which have new status), 1 resurrected combination, 1 resurrected subspecies, and 7 status changes.

## Introduction

*Elymnias* Hübner, 1818 (Nymphalidae: Satyrinae) is a species-rich and widespread butterfly genus distributed throughout the Old World tropics (Aoki et al. 1982). It derives its name from *Elymnias jynx* Hübner, 1818 (now *Elymnias hypermnestra hypermnestra* (Linnaeus, 1763)) (Fruhstorfer 1907). The genus’ widespread range and remarkable wing pattern diversity, together with the economic importance of several species, has attracted the attention of taxonomists and agricultural entomologists for centuries (Merrett 1993; Wallace 1869). However, the entire group has not been examined systematically in over 100 years (Fruhstorfer 1907; 1911), and no studies to date have used genetic data to substantiate taxonomic hypotheses. Most species are found in the Indo-Australian Archipelago, a geographically complex and geologically dynamic area with over 20,000 islands that are likely to have contributed to diversification in this taxon (Lohman et al. 2011).

We recognize a single Afrotropical species with two subspecies and 52 Australasian species with 181 subspecies distributed from Nepal to Sri Lanka in the west, throughout tropical and subtropical Asia, and extending east to Taiwan and south to Australia’s Cape York and the Bismark Archipelago of Papua New Guinea. A few species are widespread across several countries or landmasses, but many are restricted to single islands. Several new species have been discovered recently (Monastyrskii 2004; Okubo 2010; Saito and Koshi 2012), highlighting the rarity of many species and their predilection for relatively inaccessible locales, such as high mountains and remote islands.

Species in the genus differ markedly in wing color, pattern, shape, and size, making *Elymnias* one of the most morphologically heterogeneous butterfly genera (Feltwell 1993). This morphological diversity is apparently because most species are Batesian mimics of strikingly different, unpalatable model butterfly species (Corbet 1933; 1943). Many *Elymnias* species are monomorphic; conspecific males and females mimic the same model species (*e.g.*, *E. paradoxa* and *E. vasudeva*). However, some species are sexually dimorphic mimics (Moore 1894; Punnett 1915; Vane-Wright 1976), with males and females mimicking different models and differing markedly in wing color and pattern (*e.g.*, *E. kuenstleri* and *E. harterti*; Corbet et al. 1992; Parsons 2000). A few sexually dimorphic *Elymnias* species mimic a single sexually dimorphic model species, such as *E. casiphone* and *E. saueri*, which both mimic *Euploea mulciber*. A few species exhibit variability in sexual dimorphism: males and females in some populations mimic the same model species and are monomorphic, while the same species is strongly dimorphic in other locales (*e.g.*, *E. hypermnestra* and *E. agondas*).

Larvae of all species with known life histories feed exclusively on palms (Arecaceae) (Bascombe et al. 1999; Ek-Amnuay 2012; Parsons 2000; Robinson et al. 2017), and several species are also agricultural pests on economically important oil palm, *Elaeis guineensis* (Koh and Gan 2007; Merrett 1993). Adults are known to feed on exudates from rotting fruit (Treadaway and Schroeder 2012). Palm-feeding butterflies are not known to sequester noxious secondary compounds from their larval hosts, and naïve avian predators readily consumed adult *E. hypermnestra* in laboratory trials (S.-H. Yen, unpublished results), supporting the hypothesis that *Elymnias* are Batesian and not Müllerian mimics.

After the description of Linnaeus’s *Papilio hypermenstra* (now *Elymnias hypermnestra hypermnestra*) in 1763, the accumulation of new species/subspecies publications was slow and confined to few localities, for instance, *E. nesaea* (Linnaeus, 1764) from Java, *E. panthera* (Fabricius, 1787) from Borneo, and *E. vitellia* (Stoll, [1781]) from Ambon. Most species and subspecies were described during between the middle of the 19^th^ century and early 20^th^ century in the following works: Hewitson (1858; 1861; 1867; 1874; South East Asian Islands), Felder and Felder (1860; 1863; 1867; Philippines), Butler (1867; 1871; 1874; 1882; 1883; Asia), Distant (1882a; b; 1883; 1886; Malaysia), Semper (1887; 1892; Philippines), Grose-Smith (1889; 1892; 1894a; b; 1897; South East Asian Islands), Staudinger (1889; 1894a; b; Palawan and New Guinea), de Nicéville (1890; 1893; 1895; 1898; 1900; 1902; Indo-Malaya) , Moore (1857; 1875; 1878a; b; 1880; 1886; 1894; Indian subcontinent and Indochina), Fruhstorfer (1894a; b; c; 1895a; b; 1896a; b; c; 1898a; b; c; 1899; 1900; 1902a; b; c; 1904a; b; c; d; Southeast Asia; 1911), Rothschild (1915a; b; c; d; islands surrounding New Guinea), Hemming (1967; global), and Talbot (1929; 1932; Malaysia). Several new taxa have been discovered during the last 40 years, including species described by Jumalon (1975; Philippines), Tsukada and Nishiyama (1979; Southeast Asia), Tateishi (2001; Southeast Asia), Uémura and Kitamura (2001; Philippines), Monastyrskii (2004; the Indochina), Suzuki (2006; the Andaman Islands), Okubo (2010; the Lesser Sunda Islands), and Saito and Koshi (2012; Indochina).

The first checklist of *Elymnias* was compiled by Wallace (1869), followed by Butler (1871); Gaede (1931) enumerated the *Elymnias* species recognized at that time. The most recent comprehensive generic revision was completed by Fruhstorfer (1907; 1911), who recognized over 200 species-level taxa. This most recent study established the genus’ higher classification, infrageneric grouping, geographical distributions, diagnostic characters, and synonyms. He recognized *Elymniopsis* Westwood, [1851] (now a junior synonym of *Elymnias*) as a valid genus and separated *Elymnias* into two subgenera: *Elymnias* and *Mimadelias* Moore, 1894. However, Fruhstorfer’s taxonomic treatment relied entirely on comparisons of wing shape and color pattern; he did not examine genitalia. After this work, a few additional publications provide regional checklists with images of adults, including: Aoki and Uémura (1982), D’Abrera (1985), Pinratana (1988), Corbet et al. (1992), Osada et al. (1999), Chou (2000), Monastyrskii (2005), Ek-Amnuay (2012), Treadaway and Schroeder (2012), and Inayoshi (2017). These works, together with G. Lamas’ catalog of butterfly names established the taxonomic groundwork for the genus. Prior to the present study, *ca.* 47 species and 190 subspecies were recognized (G. Lamas, *pers. comm*.).

This checklist enumerates and verifies all current combinations and synonyms, and provides original literature citations, type localities, repositories of type specimens, photographs of specimens and genitalia, and maps of each subspecies’ geographical range and type locality. Integrative taxonomic practice employing multi-locus molecular phylogenetics in concert with data from wing and genitalic characters has informed taxonomic decisions to retain or revise contentious classification and nomenclature. This checklist is meant to clarify taxonomic problems in the genus and aid biologists interested in studying butterfly biodiversity, but will also serve as a framework for future studies on the phylogeny, biogeography, wing pattern evolution, and speciation of this fascinating radiation of Batesian mimetic butterfly species.

## Material and methods

### Examination of original literature and type specimens

The taxonomic changes we propose are based on examinations of hundreds of specimens in dozens of museums, quantification of wing and genitalic characters including over 100 dissections of males and females (Wei et al. in prep.), and a multi-locus molecular phylogenetic analysis based on six genetic markers from over 200 specimens including nearly every species that we recognize (Lohman et al. in prep.).

Verification of type specimens was based on information provided in the original literature as well as critical review of the collection of specific authors, especially Fruhstorfer. All taxonomic treatments proposed in the present study, including the availability of infrasubspecific taxa, follow regulations and suggestions of the latest version of ICZN (1999).

All publications with original descriptions of new taxa or describing new taxonomic acts were consulted to verify the status and collection localities of type material. Geographical information was obtained directly from specimen labels and from literature to provide accurate locality data and minimize misinterpretation of geographical localities caused by misidentified or mislabeled specimens.

All images of specimens photographed in various museum collections are used here with permission from each museum. Except for the photographs provided by KUTH (Department of Entomology, Kasetsart University), David J. Lohman, and the Museum of Comparative Zoology, Harvard University, all the other photographs were taken by Chia-Hsuan Wei and Shen-Horn Yen.

The following abbreviations are used to specify the repository of type material. Specimens, including type specimens, were borrowed and/or photographed from many of these institutions and private collections.

### Abbreviations of specimen repositories

**DNPFIC** Forest Insect Collection, Department of National Parks, Wildlife and Plant Conservation, Thailand

**ECMP** Entomology Collection, Bureau of Science, Manila, Philippines

**FMNH** Field Museum of Natural History, Chicago, USA

**HPC** Hiroto Hanafusa Private Collection, Japan

**HSPC** Hiroyuki Soeda Personal Collection, Japan

**IM** Indian Museum, Calcutta, India

**IPC** Yutaka Inayoshi Private Collection, Chiang Mai, Thailand

**JPC** Julian Jumalon Private Collection, Cebu City, Philippines

**KMSPC** Kazu-Michi Suzuki Private Collection, Japan

**KUTH** Department of Entomology, Kasetsart University, Thailand

**LSL** Linnaean Society of London, London, London, UK

**MCZ** Museum of Comparative Zoology, Harvard University, USA

**MEPR** Museo Entomologico Pietro Rossi, Duino, Italy

**NODAI** Tokyo University of Agriculture, Tokyo, Japan

**MUS** Malaysia Universiti Sarawak, Kota Samarahan, Malaysia

**NBC** National Biodiversity Center, Leiden, Netherlands

**NHM** The National History Museum, London, UK

**NHMT** The National History Museum at Tring, Tring, UK

**NHMW** Vienna Museum of Natural History, Vienna, Austria

**NMNH** National Museum of Natural History, USA

**NRM** Naturhistoriska Riksmuseet, Stockholm, Sweden

**NSYSU** National Sun Yat-Sen University, Kaohsiung, Taiwan

**NWSUAF** Northwest A & F University, Shaanxi, China

**OMPC** Kikumaro Okano Private Collection, Japan

**OPC** Kiyoshi Okubo Private Collection, Japan

**PNM** National Museum of the Philippines, Manila, Philippines

**SMFD** Naturmuseum Senckenberg, Frankfurt am Main, Germany

**SMK** Sarawak Museum Kuching, Sarawak, Malaysia

**SMTD** Staatliches Museum für Tierkunde, Dresden, Germany

**SPC** Kotaro Saito Private Collection, Japan

**TME** Toyosato Museum of Entomology, Tsukuba, Japan

**TPC** Tsukada Private Collection, Japan

**UPC** Yoshinobu Uémura Private Collection, Japan

**ZMHB** Museum für Naturkunde, Berlin, Germany

**ZMUC** Zoological Museum University of Copenhagen, Copenhagen, Denmark

### Species concept and integrative taxonomic approach

We ascribe to the Biological Species Concept, which defines species as reproductively isolated groups of populations (Mayr 1940). We have attempted to recognize reproductive isolation between species by identifying coordinated morphological and/or genetic differences among species in several traits. We also expect that species should not be polyphyletic and that species should generally be monophyletic after sufficient time since divergence from their sister taxon. We regard subspecies as phenotypically distinctive geographic variants and do not expect them to be monophyletic or reproductively isolated from other subspecies (Braby et al. 2012). However, a subspecies should be differentiable from other conspecific subspecies using morphology, genetic data, or other characteristics.

Given the within-species morphological variability known from this and other mimetic butterfly taxa (Punnett 1915), we adopted the following procedures for evaluating the validity of prior taxonomic hypotheses: (1) We reconstructed phylogenies using both morphological (Wei et al. in prep.) and molecular data (Lohman et al. in prep.), and used these as guides for interpreting relationships among species and taxonomic boundaries within species complexes; (2) In these analyses, we endeavored to include specimens from the type localities (or the surrounding area—at least the same island group) of the nominotypical subspecies to substantiate taxonomic boundaries of geographically widespread species with multiple subspecies (*e.g.*, *agondas*, *casiphone*, *hypermnestra*, *nesaea*, and *panthera*); (3) We considered the geological history of a species’ range (Hall 2001; Hall and Smyth 2008; Sathiamurthy and Voris 2006), particularly for taxa that are rare in museum collections and not readily available for morphological or molecular study. For example, present-day Sulawesi comprises multiple terranes, some of which originated in different biogeographical subregions, that collided in the Miocene (Lohman et al. 2011; Stelbrink et al. 2012). If two or more subspecies of the same species are described from a large and geologically complex island such as Sulawesi or New Guinea and we had limited material for phylogenetic study, then we generally retained the landmass’s different subspecies for lack of evidence to synonymize them; (4) When genetic and/or ecological data suggested that different names had been applied to different mimetic forms, sexes, or seasonal forms, we synonymized these taxa.

For convenience, we have divided the genus into 15 monophyletic species groups (Lohman et al. in prep.) named after each clade’s oldest named species. Because of the uniformity of genitalia and extreme intraspecific variability in wing patterns, there are few if any morphological synapomorphies that can be used to discriminate these species groups. They have been circumscribed based on relatedness as inferred by a multi-locus molecular phylogeny.

### Distribution maps and type localities

A variety of sources were used to infer the distribution maps that we provide, including museum data, taxonomic and other publications (Aoki et al. 1982; Braby 2000; Ek-Amnuay 2012; Hanafusa 2001; Inayoshi 2017; Monastyrskii 2004; 2005; Okubo 2010; Parsons 2000; Saito and Koshi 2012; Suzuki 2006; Tateishi 2001; Treadaway and Schroeder 2012; Uémura and Kitamura 2001; Vane-Wright and de Jong 2003). The majority of *Elymnias* taxa were described when most of South and Southeast Asia were colonized by European countries, and many of the type locality names given in the species descriptions have changed since colonial times. Therefore, in addition to the original type locality names given in the species description, we have attempted to provide the modern locality names in parentheses. In the text below, we do not attempt to use present knowledge of the taxon’s distribution to infer the precise location where the type was collected. However, when designating type localities on the distribution maps, we have attempted to use knowledge of the taxon’s current distribution and other information to indicate the type locality as precisely as possible. Nonetheless, many type localities are imprecise and cannot be localized because many labels simply list the island where the specimen was found (*e.g*., New Guinea) rather than a precise locality.

### Format of the checklist

This annotated checklist is formatted in the following way:

***valid species name* author, year**

Specimens: Figs X, Y, Z; Male genitalia: Figs X, Y, Z; Distribution: Fig: X

**ssp. *recognized valid subspecies name* , author, year**

*Original combination of subspecies*, Author, Year. **TL**: Type locality provided in original description (Current name of type locality in a standardized format- Country: Province, locality). **TS**: Depository of type specimen. Original citation.

*Junior synonym original combination*, Author, Year. **TL**: Type locality provided in original description (Current name of type locality); **TS**: Depository of type specimen. Original citation.

## Checklist of *Elymnias*

***ELYMNIAS* Hübner, 1818 (Type species: *Elymnias jynx* Hübner**, 1818, = *Papilio hypermnestra* Linnaeus, 1763) *Zuträge Samml. exot. Schmett.* 1:12.

*Didonis* Hübner, [1819] (Type species: *Papilio vitellia* Stoll, 1781)^1^

*Verz. bek. Schmett.* 2: 17.

*Dyctis* Boisduval, 1832 (Type species: *Dyctis agondas* Boisduval, 1832)

*Voy. Astrolabe.* 1: 138.

*Agrusia* Moore, 1894 (Type species: *Melanitis esaca* Westwood, 1851)

*Lepidoptera Indica* 2 (18): 169.

*Bruasa* Moore, 1894 (Type species: *Melanitis penanga* Westwood, 1851)

*Lepidoptera Indica* 2 (18): 164–165.

*Melynias* Moore, 1894 (Type species: *Papilio lais* Cramer, [1777])

*Lepidoptera Indica* 2 (18): 156–163.

*Mimadelias* Moore, 1894 (Type species: *Elymnias vasudeva* Moore, 1858)^2^

*Lepidoptera Indica* 2 (18): 165–168.

*Elymniopsis* Fruhstorfer, 1907 (Type species: *Papilio phegea* Fabricius, 1793)^3^

*Dt. ent. Z. Iris* 20 (3): 173–174.

### *bammakoo*-group

***bammakoo* (Westwood, 1851)**

**Figure 1.**
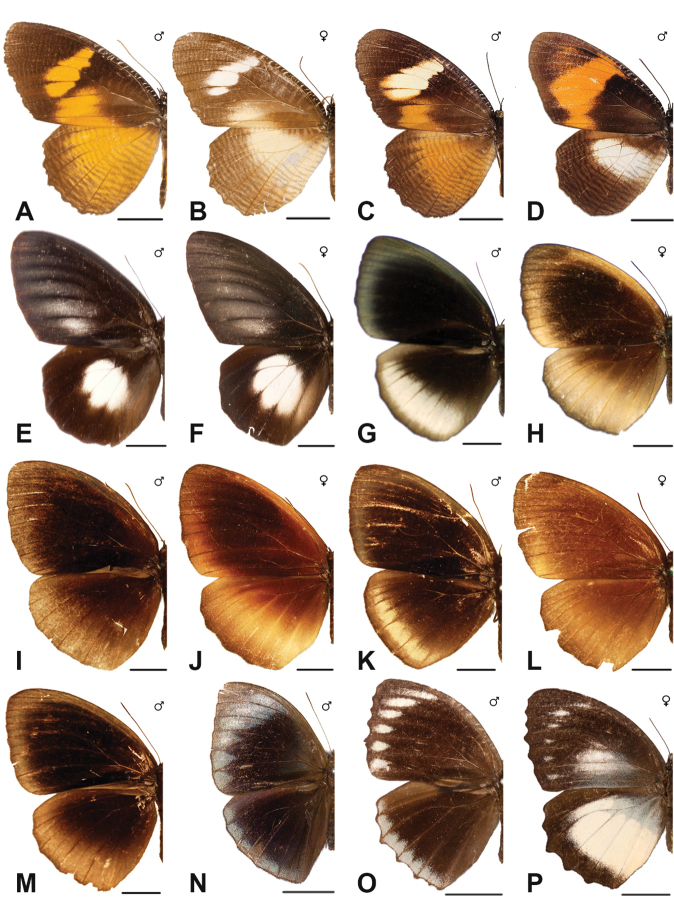
**A** *bammakoobammakoo* ♂ D NHM central Africa **B** *bammakoobammakoo* ♀ D NHM collection locality unknown **C** *bammakoobammakoo* ♂ D NMNH Central African Republic: Bangui **D** *bammakoorattrayi* ♂ D NMNH Uganda: Bwamba **E** *paradoxa* ♂ D NHM Indonesia: Papua, Weyland Mountains **F** *paradoxa* ♀ D NHM Indonesia: Papua, Weyland Mountains **G** *papuapapua* ♂ D NHM Indonesia: Papua, Yos Sudarso Bay; Syntype of *Elymnias papua viridescens* **H** *papuapapua* ♀ D NHM Indonesia: Papua, Yos Sudarso Bay; Syntype of *Elymnias papua viridescens* **I** *papuapapua* ♂ D NHM Papua New Guinea **J** *papuapapua* ♀ D NHM Papua New Guinea **K** *papuacinereomargo* ♂ D NHM Indonesia: Papua, Biak **L** *papuacinereomargo* ♀ D NHM Indonesia: Papua, Biak **M** *papualactentia* ♂ D NHM Indonesia: West Papua, Raja Ampat Regency, Waigeo **N** *esacaandersonii* ♂ D KUTH Thailand: Yala, Than To **O** *esacamaheswara* ♂ D NHM Indonesia: Java **P** *esacamaheswara* ♀ D NHM Indonesia: Java.

Specimens: Fig. 1A–D; Male Genitalia: Fig. 22A; Distribution: Fig. 27

**ssp. bammakoo (Westwood, 1851)**

*Papilio phegea* Fabricius, 1793. **TL**: Uganda. **TS**: ZMUC. (preoccupied by *Papilio phegea* Borkhausen, 1788). *Ent. Syst.* 3 (1): 132.

*Melanitis bammakoo* Westwood, 1851. **TL**: Ashanti (Ghana: Ashanti Region). **TS**: NHM. *Gen. diurn. Lep.* 2: 405, pl. 68, fig. 3.

Elymnias phegea var. intermedia Aurivillius, 1898. **TL**: not indicated. **TS**: NRM. *K. svenska Vetenskakad. Handl.* 31 (5): 45.

Elymnias phegea ab. angustata Bartel, 1905. **TL**: Kamerun, Barombi Station (Cameroon: Southwest Region, Barombi Mbo). **TS**: unknown. *Novit. Zool.* 12: 129.

Elymiopsis bammakoo var. hybrida Niepelt, 1915. **TL**: Kassai River (Democratic Republic of Congo: Kasai River). **TS**: NHM. *Int. Ent. Zs.* 9: 58.

*Elymniopsis lise* Hemming, 1960. **TL**: Uganda. **TS**: ZMUC. (replaced *Papilio phegea* Fabricius, 1793). *Annot. lep.* 1: 30.

**ssp. rattrayi Sharpe, 1902**

*Elymnias rattrayi* Sharpe, 1902. **TL**: Entebbe (Uganda: Central Uganda, Entebbe). **TS**: NHM. *Entomologist* 35: 41.

*Elymnias ugandae* Grünberg, 1908. **TL**: Uganda. **TS**: unknown. *Sitzungsber. Ges. Naturf. Freunde. Berlin.* 1908: 51.

Elymniopsis ugandae f. rattrayi Lewis, 1974. **TL**: Uganda. **TS**: unknown. *Butterflies of the World*, p. 266, pl. 115, fig. 15.

*Elymniopsis bammakoo rattrayi* (Sharpe, 1902). **TL**: Uganda. **TS**: unknown. *Butterflies of West Africa*, p. 283.

### *paradoxa*-group

***paradoxa* Staudinger, 1894**

Specimens: Fig. 1E–F; Male Genitalia: Fig. 22B; Distribution: Fig. 28

*Elymnias paradoxa* Staudinger, 1894**. TL**: Kubary (Papua New Guinea: Madang Province, Mt. Kubari). **TS**: ZMHB. *Dt. ent. Z. Iris* 7 (1): 116.

*Elymnias erastus* Grose-Smith, 1894. **TL**: Sattelberg (Papua New Guinea: Morobe Province, Huon Peninsula, Sattelberg). **TS**: NHM. *Novit. Zool.* 1 (3): 588.

### *papua*-group

***papua* Wallace, 1869**

Specimens: Fig. 1G–M; Male Genitalia: Fig. 22C; Distribution: Fig. 29

**ssp. papua Wallace, 1869**^4^

*Elymnias papua* Wallace, 1869. **TL**: New Guinea^5^. **TS**: NHM. *Trans. Ent. Soc. Lond.* 1869 (4): 329.

*Elymnias viridescens* Grose-Smith, 1894. **TL**: Humboldt Bay (Indonesia: Papua, Jayapura, Yos Sudarso Bay). **TS**: NHM. *Novit. Zool.* 1(2): 365, pl. 12, figs 5–6.

Dyctis viridescens var. kakarona Hagen, 1897. **TL**: Sattelberg (Papua New Guinea: Morobe Province, Huon Peninsula, Sattelberg). **TS**: NHMT. *Jarhb. Nass. Ver. Nat.* 50: 78.

*Elymnias papua bivittata* van Eecke, 1915. **syn. n. TL**: Bivakeiland, Koofbivak, New Guinea (Indonesia: Papua, Asmat Regency, Bivak Island). **TS**: NBC. *Nova Guinea* 13 (1): 65, pl. 3, fig. 5 & 5a.

**ssp. lactentia Fruhstorfer, 1907**

*Elymnias papua lactentia* Fruhstorfer, 1907. **TL**: Waigiu Island (Indonesia: West Papua, Raja Ampat Regency, Waigeo). **TS**: NHM. *Dt. ent. Z. Iris* 20 (3): 240.

**ssp. cinereomargo Joicey & Noakes, 1915**

*Elymnias viridescens cinereomargo* Joicey & Noakes, 1915. **TL**: Biak Island (Indonesia: Papua, Biak). **TS**: NHM. *Trans. ent. Soc. Lond.* 1915 (2): 196.

**ssp. climena Talbot, 1932**

*Elymnias climena* Talbot, 1932. **TL**: Mysol Island (Indonesia: West Papua, Raja Ampat Regency, Misool). **TS**: NHM. *Bull. Hill Mus. Witley* 4 (3): 168.

**ssp. euploeoides Talbot, 1932**

*Elymnias euploeoides* Talbot, 1932. **TL**: Batchian (Indonesia: North Maluku, Bacan). **TS**: NHM. *Bull. Hill Mus. Witley* 4 (3): 167.

### *esaca*-group

***esaca* (Westwood 1851)**^6^

**Figure 2.**
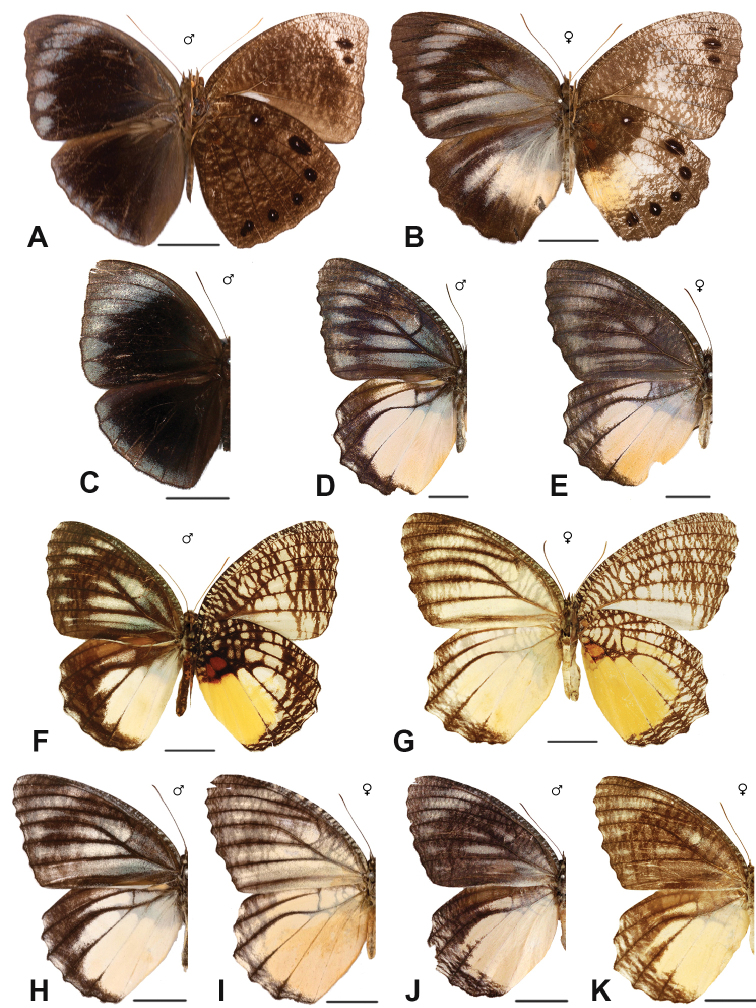
**A** *esacaleontina* ♂ D+V NHM Indonesia: North Sumatra, Nias **B** *esacaleontina* ♀ D+V NHM Indonesia: North Sumatra, Nias **C** *esacaesaca* ♂ D KUTH Thailand: Yala, Than To **D** *vasudeva* ♂ D KUTH Thailand: Kanchanburi, Sri Sawat **E** *vasudeva* ♀ D KUTH Thailand: Chaiyaphum, Phu Khieo **F** *vasudeva* ♂ D+V NHM India: Assam **G** *vasudeva* ♀ D+V NHM India: Assam **H** *vasudeva* ♂ D NHM India: Meghalaya, Khasi Hills **I** *vasudeva* ♀ D NHM India: Meghalaya, Khasi Hills **J** *vasudeva* ♂ D DNPFIC Thailand: Kanchanaburi **K** *vasudeva* ♀ D NHM India: Assam.

Specimens: Figs 1N–P, 2A–C; Male Genitalia: Fig. 22D–E; Distribution: Fig. 30

**ssp. esaca (Westwood, 1851)**

*Melanitis esaca* Westwood, 1851. **TL**: East Indies. **TS**: NHM. *Gen. diurn. Lep.* 2: 405.

*Elymnias godferyi* Distant, 1883. **TL**: Sungei Ujong (Peninsular Malaysia: Negeri Sembilan, Sungei Ujong). **TS**: NHM. *Ann. Mag. nat. Hist.* 12 (71): 351.

*Dyctis esacoides* de Nicéville, [1893]. **TL**: Perak (Peninsular Malaysia: Perak), Battak Mountains^7^ (Indonesia: North Sumatra). **TS**: IM. *J. Bomb. Nat. Hist. Soc.* 7 (3): 323, pl. H, fig. 2.

**ssp. egialina (C. & R. Felder, 1863)**

*Melanitis egialina* C. & R. Felder, 1863. **TL**: Luzon (Philippines: Luzon). **TS**: NHMW.

*Wien. ent. Monats.* 7 (4): 121.

*Melanitis ligya* C. & R. Felder, 1863. **nom. nud. TL**: Luzon (Philippines: Luzon). **TS**: NHMW. *Wien. ent. Monats.* 7 (4): 121.

*Melanitis pallas* C. & R. Felder, 1863. **nom. nud. TL**: Luzon (Philippines: Luzon). **TS**: NHMW. *Wien. ent. Monats.* 7 (4): 121.

**ssp. borneensis Wallace, 1869**^8^

*Elymnias borneensis* Wallace, 1869. **TL**: Sarawak (East Malaysia: Sarawak). **TS**: NHM. *Trans. ent. Soc. Lond.* 1869 (4): 324.

Elymnias (Mimadelias) esaca taeniola Fruhstorfer, 1907. **syn. n. TL**: southeast Borneo. **TS**: NHM. *Dt. ent. Z. Iris* 20 (3): 250.

**ssp. andersonii (Moore, 1886)**^9^

*Dyctis andersonii* Moore, 1886. **TL**: Mergui (Myanmar: Thanintharyi, Mergui Archipelago). **TS**: NHM. *J. Linn. Soc. Lond.* 21 (1): 33, pl. 3, fig. 5.

*Elymnias* (*Mimadelias) oberthuri* Fruhstorfer, 1902. **syn. n. TL**: Renong, Siam (Thailand: Ranong). **TS**: NHM. *Soc. Ent.* 17 (11): 82.

**ssp. maheswara Fruhstorfer, 1894**

Elymnias (Dyctis) maheswara Fruhstorfer, 1894. **TL**: Gede Vulcan (Indonesia: West Java, Mt. Gede). **TS**: NHM. *Ent. Nachr.* 20 (2): 21.

**ssp. leontina Fruhstorfer, 1898**

*Elymnias esaca leontina* Fruhstorfer, 1898. **TL**: Nias (Indonesia: North Sumatra, Nias). **TS**: NHM. *Ent. Zs.* 12 (14): 99.

**ssp. pseudodelias Fruhstorfer, 1907**

Elymnias (Mimadelias) esaca pseudodelias Fruhstorfer, 1907. **TL**: Sumatra (Indonesia: Sumatra). **TS**: NHM. *Dt. ent. Z. Iris* 20 (3): 250.

**ssp. georgi Fruhstorfer, 1907**

Elymnias (Mimadelias) esaca georgi Fruhstorfer, 1907. **TL**: Mindanao (Philippines: Mindanao). **TS**: NHM. *Dt. ent. Z. Iris* 20 (3): 251.

**ssp. saifuli Hanafusa, 1993**

*Elymnias esaca saifuli* Hanafusa, 1993. **TL**: Siberut Island (Indonesia: West Sumatra, Mentawai Islands, Siberut). **TS**: HPC. *Futao* (11): 3.

**ssp. popularis Hanafusa, 1994**

*Elymnias esaca popularis* Hanafusa, 1994. **TL**: Tanahmasa Island (Indonesia: North Sumatra, South Nias Regency, Batu Islands, Tanahmasa). **TS**: HPC. *Futao* (17): 19.

**ssp. splendida Tateishi, 2001**

*Elymnias esaca splendida* Tateishi, 2001. **TL**: Singkep Island (Indonesia: Riau Islands, Lingga Archipelago, Singkep Island). **TS**: FMNH. *Futao* (39): 13.

**ssp. lingga Tateishi, 2001**

*Elymnias esaca lingga* Tateishi, 2001. **TL**: Lingga Island (Indonesia: Riau Islands, Lingga Archipelago, Lingga Island). **TS**: FMNH. *Futao* (39): 14.

**ssp. nigricans Tateishi, 2001**

*Elymnias esaca nigricans* Tateishi, 2001. **TL**: Enggano Island (Indonesia: Bengkulu, Enggano Island). **TS**: FMNH. *Futao* (39): 14.

**ssp. andrewi Schröder & Treadaway, 2003**

*Elymnias esaca andrewi* Schröder & Treadaway, 2003. **TL**: Philippines: Oriental Mindoro, Mt. Halcon. **TS**: SMFD. *Nachr. ent. Ver. Apollo* 23 (4): 193, pl. 1, figs 3–4.

**ssp. leytensis Schröder & Treadaway, 2003**

*Elymnias esaca leytensis* Schröder & Treadaway, 2003. **TL**: Philippines: Southern Leyte, Saint Bernard, Hinabian. **TS**: SMFD. *Nachr. ent. Ver. Apollo* 23 (4): 194, pl. 1, figs 7–8.

**ssp. tateishii Lamas, 2010**

*Elymnias esaca tateishii* Lamas, 2010. *SHILAP* 38 (150): 198. (replacement name of *Elymnias esaca lautensis* Teteishi, 2001).

*Elymnias esaca lautensis* Tateishi, 2001. **TL**: Laut Island (Indonesia: South Kalimantan, Kota Baru, Laut Island). **TS**: FMNH. *Futao* (39): 13. (preoccupied by *Elymnias harterti lautensis* Medicielo & Hanafusa, 1994).

***vasudeva* Moore, 1857**^10^

Specimens: Fig. 2D–K; Male Genitalia: Fig. 22F; Distribution: Fig. 31

**ssp . *vasudeva* Moore, 1857**

Elymnias (Mimadelias) vasudeva vasudeva Moore, 1857. **TL**: Darjeeling (India: West Bengal, Darjeeling). **TS**: NHM. *Cat. lep. Ins. Mus. East India Coy.* 1: 238.

*Elymnias thycana* Wallace, 1869. **syn. n. TL**: India. **TS**: NHM. *Trans. ent. Soc. Lond.* 1869 (4): 323. ^(8)^

*Mimadelias deva* Moore, 1894. **syn. n. TL**: Khasia Hills, Assam (India: Meghalaya, Khasi Hills). **TS**: NHM. *Lepid. Ind.* 2 (19): 167, pl. 142, fig. 2a.

*Mimadelias burmensis* Moore, 1893. **syn. n. TL**: Tenasserim (Myanmar: Tanintharyi, Tenasserim). **TS**: NHM. *Lepid. Ind.* 2 (19): 168, pl. 143, fig. 1a–e.

*Elymnias vacudera* [sic] *sinensis* Chou, Zhang & Xie, 2000. **syn. n. TL**: Yunnan (China: Yunnan). **TS**: NWSUAF. *Entomotaxonomia* 22 (3): 224, figs 7–8.

### *dara*-group

***dara***﻿﻿ **Distant & Pryer, 1887**

**Figure 3.**
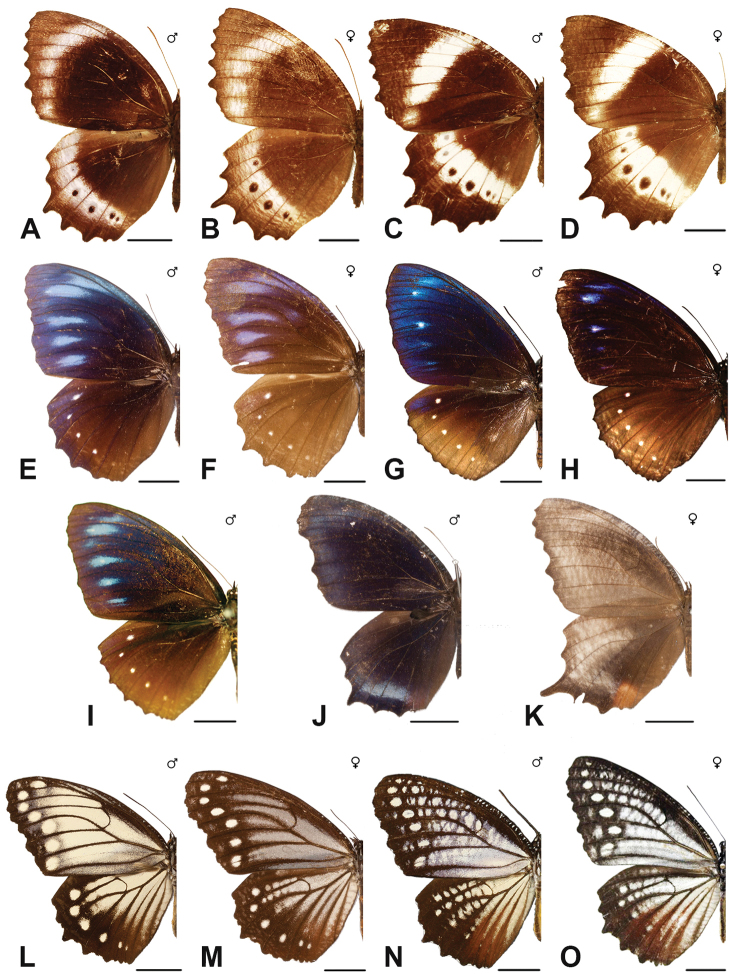
**A** *darabengena* ♂ D NHM Indonesia: West Java **B** *darabengena* ♀ D NHM Indonesia: West Java **C** *daradaedalion* ♂ D NHM Myanmar **D** *daradaedalion* ♀ D NHM Myanmar **E** *patnapatna* ♂ D NHM India: Sikkim **F** *patnapatna* ♀ D NHM India: West Bengal, Darjeeling, Pedong **G** *patna* “*inayoshii*” (nomem nudum) ♂ D KUTH Thailand: Ranong; Holotype **H** *patna* “*inayoshii*” (nomen nudum) ♀ D KUTH Thailand: Trang, Khao Chong; Paratype **I** *patnahanitschi* ♂ D NHM Peninsular Malaysia **J** *peali* ♂ D NHM India: Assam, Sivasagar **K** *peali* ♀ D NHM India: Assam; Holotype **L** *ceryx* ♂ D NHM Indonesia: West Java, Mt. Gede **M** *ceryx* ♀ D NHM Indonesia: West Java, Mt. Gede **N** *ceryxoides* ♂ D MCZ Indonesia: North Sumatra, Mt. Sinabung **O** *ceryxoides* ♀ D UPC Indonesia: West Sumatra.

Specimens: Fig. 3A–D; Male Genitalia: Fig. 22G–I; Distribution: Fig. 32

**ssp. dara Distant & Pryer, 1887**

*Elymnias dara* Dinstant & Pryer, 1887. **TL**: north Borneo. **TS**: NHM. *Ann. Mag. nat. Hist.* (5) 19 (109): 50.

**ssp. albofasciata Staudinger, 1889**

*Elymnias albofasciata* Staudinger, 1889. **TL**: Philippines: Palawan. **TS**: ZMHB *Dt. ent. Z. Iris* 2 (1): 39.^11^

**ssp. deminuta Staudinger, 1889**

Elymnias albofasciata var. deminuta Staudinger, 1889. **TL**: Lawang (Indonesia: East Java, Malang, Lawang). **TS**: ZMHB. *Dt. ent. Z. Iris* 2 (1): 40.

**ssp. bengena Fruhstorfer, 1907**

*Elymnias dara bengena* Fruhstorfer, 1907. **TL**: Palabuan (Indonesia: West Java, Sukabumi, Pelabuhan Ratu). **TS**: NHM. *Dt. ent. Z. Iris* 20 (3): 216.

**ssp. darina Fruhstorfer, 1907**

*Elymnias dara darina* Fruhstorfer, 1907. **TL**: Battak Mountains (Indonesia: North Sumatra). **TS**: NHM. *Dt. ent. Z. Iris* 20 (3): 215.

**ssp. daedalion (de Nicéville, 1890)**

**Figure 4.**
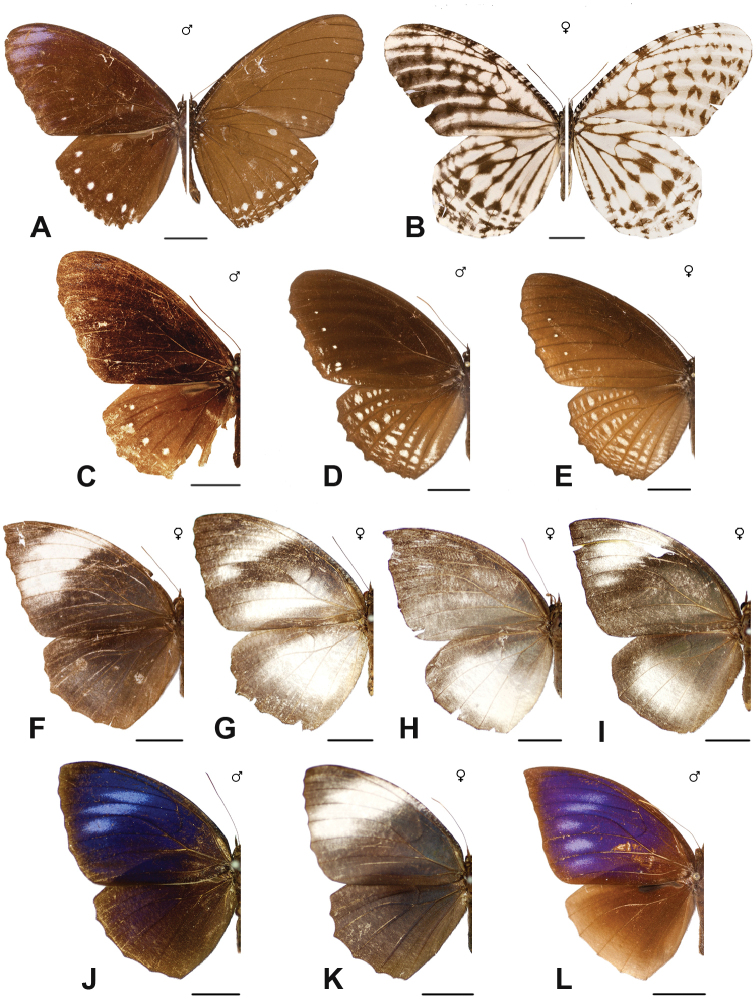
**A** *kuenstlerikuenstleri* ♂ D+V NHM Specimen locality unknown **B** *kuenstlerikuenstleri* ♀ D+V NHM Peninsular Malaysia: Selangor, Bukit Kutu **C** *kuenstleririleyi* ♂ D NHM Borneo **D** *pellucida* ♂ D NHM Malaysia: Sabah, Mt. Kinabalu **E** *pellucida* ♀ D NHM Malaysia: Sabah, Mt. Kinabalu **F** *penangapenanga* ♀ D NHM Malaysia **G** *penangapenanga* ♀ D NHM Singapore; Allotype of *Elymnias abrisa* **H** *penangapenanga* ♀ D NHM Peninsular Malaysia: Penang; Holotype of Elymnias penanga penanga f. johnsoni **I** *penangasumatrana* ♀ D NHM Indonesia: Sumatra; Holotype **J** *penangakonga* ♂ D NHM North Borneo **K** *penangakonga* ♀ D NHM North Borneo **L** *penangachelensis* ♂ D NHM Thailand: Ranong.

*Dyctis daedalion* de Nicéville, 1890. **TL**: Myittha (Myanmar: Mandalay, Kyaukse, Myittha). **TS**: IM. *J. Bomb. nat. Hist. Soc.* 5 (3): 202, pl. D, fig. 4.

### *patna*-group

***patna* (Westwood, 1851**)^12^

Specimens: Fig. 3E–I; Male Genitalia: Fig. 22J; Distribution: Fig. 33

**ssp. patna (Westwood, 1851)**

*Melanitis patna* Westwood, 1851. **TL**: East India. **TS**: NHM. *Gen. diurn. Lep.* 2: 405, pl. 68, fig. 2.

*Elymnias patna bercovitzi* Joicey & Talbot, 1921. **TL**: Five Finger Mountains (China: Hainan, Wuzhi Mountain). **TS**: NHM. *Bull. Hill Mus. Witley* 1 (1): 173.

*Melanyias patnoides* Moore, 1893. **syn. n. TL**: Burma, Karen Hills, East Pegu (Myanmar: Bago). **TS**: NHM. *Lepid. Ind.* 2 (19): 163, pl. 141, fig. 2 & 2a.

*Elymnias patna stictica* Fruhstorfer, 1902. **syn. n. TL**: Than-Moi, Nordtonkin (Vietnam: Lang Son, Than Moi). **TS**: NHM. *Dt. ent. Z. Iris* 14 (2): 271.

**ssp. hanitschi Martin, 1909**

*Elymnias patna hanitschi* Martin, 1909. **TL**: Malayische Halbinsel (Thai-Malay Peninsula). **TS**: NHMT. *Dt. ent. Z. Iris* 22 (1): 52.

***peali* Wood-Mason, 1883**

Specimens: Fig. 3J–K; Male Genitalia: Fig. 22K; Distribution: Fig. 34

*Elymnias peali* Wood-Mason, 1883. **TL**: Aideo, Sibsagar district, Assam (India: Assam, Sivasagar). **TS**: NHM. *Ann. Mag. nat. Hist.* (5) 11: 62, pl. 2, fig. A & B.

### *ceryx*-group

***ceryx* (Boisduval, 1836)**^13^

Specimens: Fig. 3L–M; Male Genitalia: Fig. 22L; Distribution: Fig. 35

*Melanitis ceryx* Boisduval, 1836. **TL**: West Java (Indonesia: West Java). **TS**: NHM. *Hist. Nat. Ins., Spec. Gén. Lépid.* 1: pl. 9, fig. 8.

*Elymnias hestinia* Fruhstorfer, 1911. **TL**: Java (Indonesia: Java). **TS**: NHM. *Gross-Schmett. Erde* 9: 383.

***kuenstleri* Honrath, [1885**]

Specimens: Fig. 4A–C; Male Genitalia: Fig. 22M; Distribution: Fig. 36

**ssp. kuenstleri Honrath, [1885**]

*Elymnias künstleri* (=*kuenstleri*) Honrath, [1885]. **TL**: Perak and Malacca (Peninsular Malaysia: Perak and Malacca). **TS**: NHM. *Berl. ent. Z.* 29 (2): 276, pl. 8, fig. 3.

**ssp. gauroides Fruhstorfer, 1894**

*Elymnias gauroides* Fruhstorfer, 1894. **TL**: Tjisewu, West Java (Indonesia: West Java, Cisewu). **TS**: NHM. *Ent. Nachr.* 20 (3): 43.

**ssp. rileyi Corbet, 1933**

*Elymnias kuenstleri rileyi* Corbet, 1933. **TL**: Borneo. **TS**: NHM. *Stylops* 2: 132.

*Elymnias borneensis* Riley, 1923. **TL**: Borneo. **TS**: unknown. *Entomologist* 56 (717): 36.

**ssp. dohrnii de Nicéville, 1895**^14^

Elymnias (Melynias) dohrnii de Nicéville, 1895. **TL**: Bohorok, East Sumatra (Indonesia: North Sumatra, Langkat Regency, Bohorok). **TS**: IM. *J. Bomb. nat. Hist. Soc.* 10 (1): 21, pl. S, fig. 12.

*Elymnias kuenstleri mariae* Toxopeus, 1936. **syn. n. TL**: Bekoelen (Indonesia: South Sumatra, Bengkulu). **TS**: NBC. *Ent. Med. Ned. Ind.* 2: 46, fig. 1.

***ceryxoides* de Nicéville, 1895. stat. rev.**^15^

Specimens: Fig. 3N–O; Distribution: Fig. 37

Elymnias (Melynias) ceryxoides de Nicéville, 1895. **TL**: Battak Mountains (Indonesia: North Sumatra). **TS: IM.** *J. Bomb. nat. Hist. Soc.* 10 (1): 22, pl. S, fig. 13.

Elymnias ceryx ceryxoides f. nigritia Fruhstorfer, 1907. **TL**: Vulkan Singalang (Indonesia: West Sumatra, Agam Regency, Mt. Singgalang). **TS**: NHM. *Dt. ent. Z. Iris* 20 (3): 213.

***pellucida* Fruhstorfer, 1895**

Specimens: Fig. 4D–E; Male Genitalia: Fig. 22N; Distribution: Fig. 38

*Elymnias pellucida* Fruhstorfer, 1895. **TL**: Kinabalu (East Malaysia: Sabah, Mt. Kinabalu). **TS**: NHM. *Ent. Nachr.* 21 (11): 168.

*Elymnias annea* Pryer & Cator, 1894. **TL**: Borneo. **TS**: NHM. *Br. N. Borneo Herald* 12 (9): 234.

*Elymnias aroa* Shelford, 1902. **TL**: Mount Penrissen, Sarawak (East Malaysia: Sarawak, Mt. Penrissen). **TS**: SMK. *Proc. Zool. Soc. Lond.* 1902 (2): 272.

### *penanga*-group

***penanga* (Westwood, 1851)**^16^

Specimens: Fig. 4F–L; Male Genitalia: Fig. 22O; Distribution: Fig. 39

**ssp. penanga (Westwood, 1851)**

*Melanitis penanga* Westwood, 1851. **TL**: Penang (Peninsular Malaysia: Penang). **TS**: NHM. *Gen. diurn. Lep.* (2): 405.

*Melaninis mehida* Hewitson, 1863. **TL**: Singapore. **TS**: NHM. *Ill. exot. Butts.* [4] (*Melanitis*): [69], pl. [36], figs 2–3.

*Elymnias abrisa* Distant, 1886. **TL**: Province Wellesley (Peninsular Malaysia: Penang, Seberang Perai). **TS**: NHM. *Ann. Mag. nat. Hist.* 17 (102): 531.

Elymnias penanga penanga f. hislopi (♀) Eliot, 1967. **TL**: Langkawi (Peninsular Malaysia: Kedah, Langkawi). **TS**: NHM(?). *Entomologist* 100 (1244): 3.

Elymnias penanga f. immaculata Martin, 1909. **TL**: Indonesia: Sumatra. **TS**: NHMT. *Dt. ent. Z. Iris* 22 (1): 55.

Elymnias penanga penanga f. johnsoni Talbot, 1929. **TL**: Penang (Peninsular Malaysia: Penang). **TS**: NHM. *Bull. Hill Mus. Witley* 3 (1): 80.

**ssp. sumatrana Wallace, 1869**

*Elymnias sumatrana* Wallace, 1869. **TL**: Sumatra (Indonesia: Sumatra). **TS**: NHM. *Trans. ent. Soc. Lond.* 1869 (4): 325.

**ssp. konga Grose-Smith, 1889**

*Elymnias konga* Grose-Smith, 1889. **TL**: Kina Balu Mountain, (East Malaysia: Sabah, Mt. Kinabalu). **TS**: NHM. *Ann. Mag. nat. Hist.* (6) 3 (16): 317.

*Elymnias borneensis* Grose-Smith, 1892. **TL**: Northeast Borneo. **TS**: NHM. *Ann. Mag. nat. Hist.* (6) 10 (60): 428. (preoccupied by *Elymnnias borneensis* Wallace 1869)

*Elymnias penanga trepsichroides* Shelford, 1904. **TL**: North Borneo. **TS**: NHM. *J. Straits Asiat. Soc.* (41): 103. (replacement name for *Elymnias borneensis* Grose-Smith, 1892)

Elymnias penanga konga f. mehidina, Fruhstorfer, 1907. **TL**: Borneo. **TS**: NHM. *Dt. ent. Z. Iris* 20 (3): 226.

Elymnias penanga konga f. ptychandrina, Fruhstorfer, 1907. **TL**: North Borneo. **TS**: NHM. *Dt. ent. Z. Iris* 20 (3): 227.

**ssp. chelensis de Nicéville, 1890**

*Elymnias chelensis* de Nicéville, 1890. **TL**: Khasi Hills (India: Meghalaya, Khasi Hills). **TS**: IM. *J. Bomb. nat. Hist. Soc.* 5 (3): 200, pl. D, fig. 3.

### *hypermnestra*-group

***hypermnestra* (Linnaeus, 1763)**^17^

**Figure 5.**
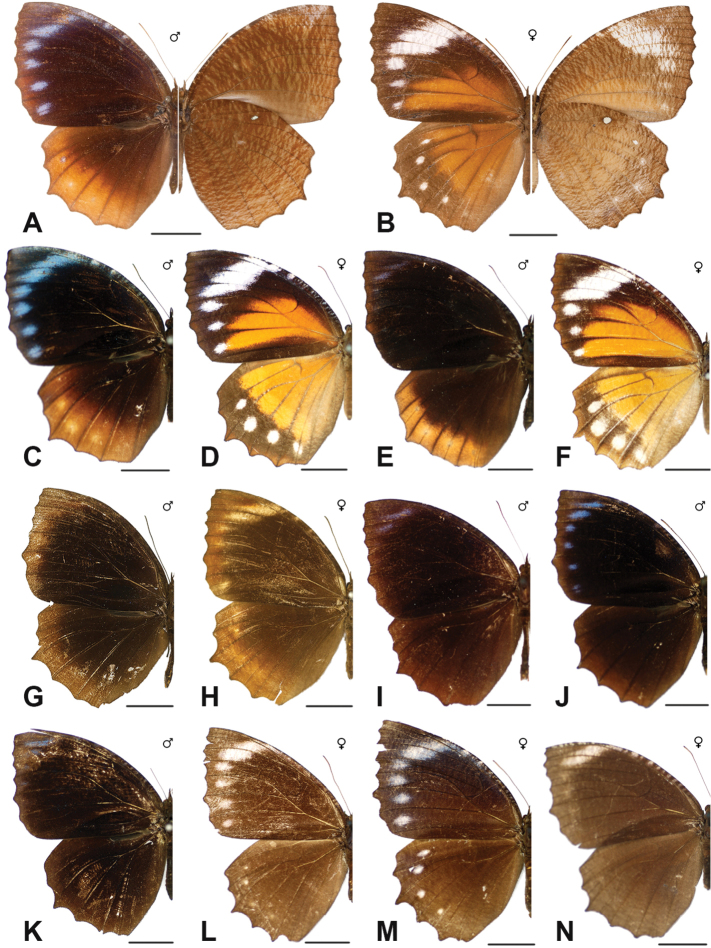
**A** *hypermnestrahypermnestra* ♂ D+V NHM Indonesia: Java, Bogor **B** *hypermnestrahypermnestra* ♀ D+V NHM Indonesia: Java, Bogor **C** *hypermnestraundularis* ♂ D NHM India: Assam **D** *hypermnestraundularis* ♀ D NHM India: Sikkim **E** *hypermnestrafraterna* ♂ D NHM Sri Lanka **F** *hypermnestrafraterna* ♀ D NHM Sri Lanka **G** *hypermnestracottonis* ♂ D NHM India: Andaman Islands **H** *hypermnestracottonis* ♀ D NHM India: Andaman Islands **I** *hypermnestranigrescens* ♂ D NHM Brunei: Tutong **J** *hypermnestranigrescens* ♂ D NHM East Malaysia: Sarawak, Mt. Marapok **K** *hypermnestranigrescens* ♂ D NHM East Malaysia: Labuan; Holotype of *Elymnias hecate* **L** *hypermnestranigrescens* ♀ D NHM East Malaysia: Sarawak, Mt. Mulu **M** *hypermnestranigrescens* “f. pseudagina” ♀ D NHM East Malaysia: Sarawak **N** *hypermnestranigrescens* ♀ D NHM Indonesia: Riau Islands.

**Figure 6.**
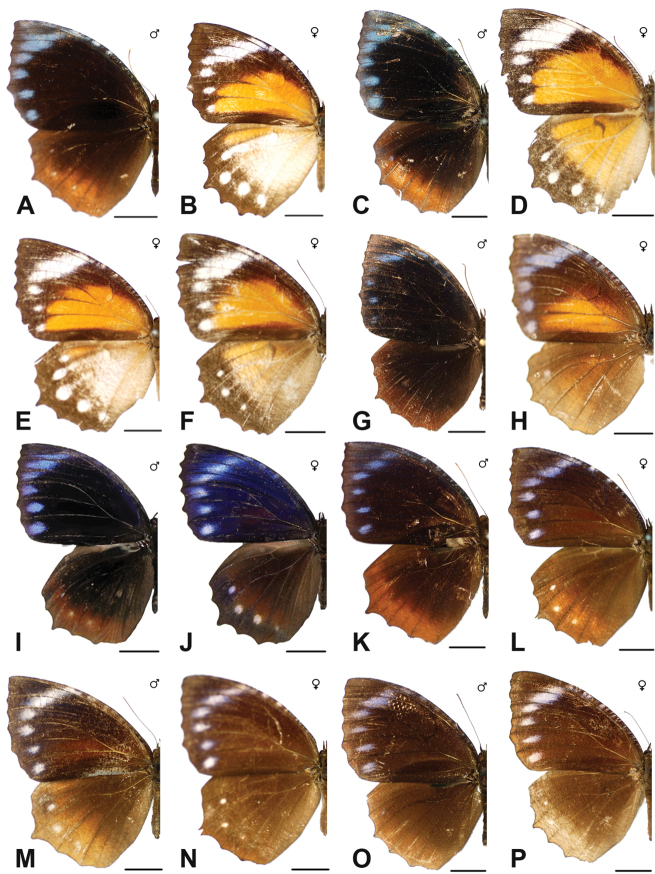
**A** *hypermnestratinctoria* ♂ D NHM Myanmar: Bago **B** *hypermnestratinctoria* ♀ D NHM Myanmar: Ayeyarwady, Pathein **C** *hypermnestrta tinctoria* ♂ D NHM Thailand: Bangkok **D** *hypermnestratinctoria* ♀ D NHM Thailand: Bangkok **E** *hypermnestratinctoria* ♀ D NHM Myanmar: Tanintharyi; Syntype of Elymnias hypermnestra tinctoria f. paraleuca **F** *hypermnestratinctoria* ♀ D NHM Peninsular Malaysia: Kedah, Langkawi Island **G** *hypermnestra discrepens* ♂ D NSYSU Peninsular Malaysia: Penang **H** *hypermnestra discrepens* ♀ D NHM Peninsular Malaysia: Penang; Allotype **I** *hypermnestrahainana* ♂ D NSYSU Taiwan: Kaohsiung **J** *hypermnestrahainana* ♀ D NSYSU Taiwan: Kaohsiung **K** *hypermnestrahainana* ♂ D NHM Vietnam **L** *hypermnestrahainana* ♀ D NHM Vietnam **M** *hypermnestrahainana* (f. depicta) ♂ D NHM Vietnam: Haiphong **N** *hypermnestrahainana* (f. depicta) ♀ D NHM Vietnam: Haiphong **O** *hypermnestraorientalis* ♂ D NHM Indonesia: East Nusa Tenggara, Flores, Ende Island; Holotype of *Elymnias nigrescens dohertyi* **P** *hypermnestraorientalis* ♀ D NHM Indonesia: East Nusa Tenggara, Flores.

**Figure 7.**
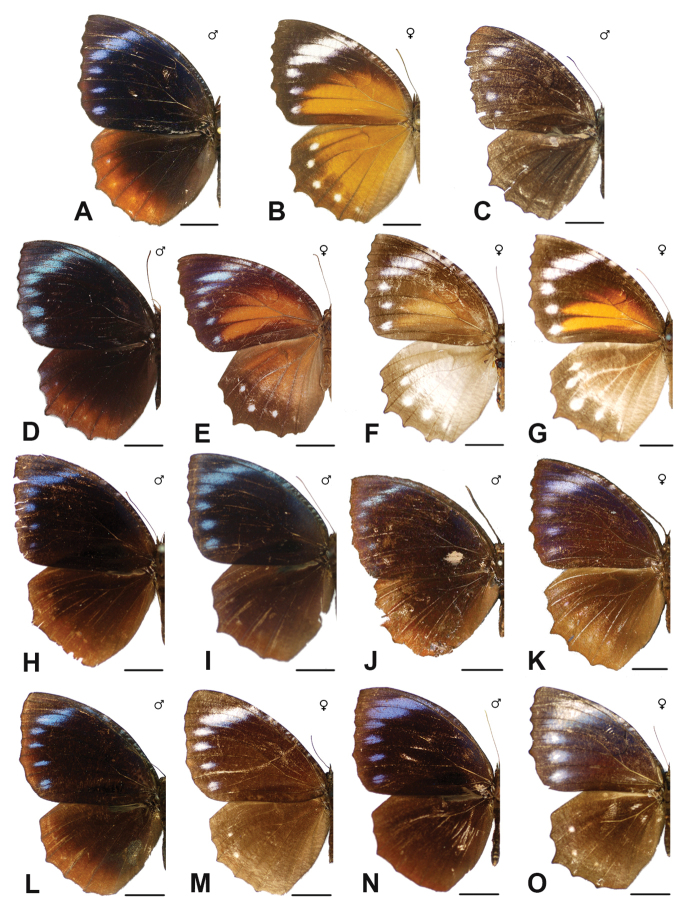
**A** *hypermnestrabaliensis* ♂ D NHM Indonesia: Bali **B** *hypernmestra baliensis* ♀ D NHM Indonesia: Bali **C** *hypermnestrabaliensis* ♂ D NHM Indonesia: Bali; Holotype of *Elymnias nigrescens bulelenga* **D** *hypermnestratinctoria* ♂ D KUTH Thailand: Chiang Mai **E** *hypermnestratinctoria* ♀ D KUTH Thailand: Chanthaburi **F** *hypermnestravioletta* ♀ D NHM Thailand: Sri Racha; Holotype of Elymnias hypermnestra violetta f. obfuscata **G** *hypermnestra meridonalis* ♀ D NHM southern Vietnam; **Holoype** of Elymnias meridionalis f. orphnia **H** *hypermnestrabeatrice* ♂ D NHM Peninsular Malaysia: Perak, Taiping **I** *hypermnestrabeatrice* ♂ D NHM Peninsular Malaysia: Pahang, Gunung Tahan **J** *hypermnestrabeatrice* ♂ D MCZ Peninsular Malaysia: Perak **K** *hypermnestrabeatrice* ♀ D MCZ Singapore **L** *hypermnestrasumbana* ♂ D NHM Indonesia: East Nusa Tenggara, Sumba **M** *hypermnestrasumbana* ♀ D NHM Indonesia: East Nusa Tenggara, Sumba **N** *hypermnestradecolorata* ♂ D NHM Indonesia: Sumatra **O** *hypermnestradecolorata* ♀ D NHM Indonesia: Sumatra.

**Figure 8.**
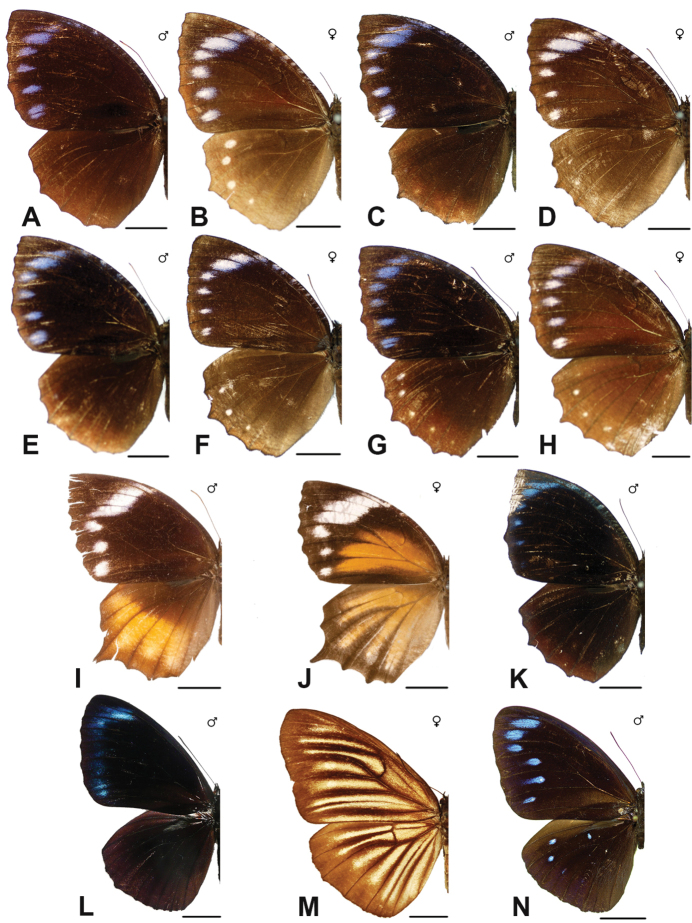
**A** *hypermnestrasumbawana* ♂ D NHM Indonesia: West Nusa Tenggara, Sumbawa **B** *hypermnestrasumbawana* ♀ D NHM Indonesia: West Nusa Tenggara, Sumbawa **C** *hypermnestratimorensis* ♂ D NHM East Timor: Dili **D** *hypermnestratimorensis* ♀ D NHM East Timor: Dili **E** *hypermnestraalorensis* ♂ D NHM Indonesia: East Nusa Tenggara, Adonara **F** *hypermnestraalorensis* ♀ D NHM Indonesia: East Nusa Tenggara, Adonara **G** *hypermnestrauemurai* ♂ D NHM Indonesia: West Nusa Tenggara, Lombok **H** *hypermnestrauemurai* ♀ D NHM Indonesia: West Nusa Tenggara, Lombok **I** *caudata* ♂ D NHM Myanmar (specimen is likely mislabeled) **J** *caudata* ♀ D NHM India: Kerala, Malabar **K** *merula* ♂ D NHM Sri Lanka: Central Province, Kandy; Holotype **L** *nepheronidesnepheronides* ♂ D HSPC Indonesia: East Nusa Tenggara, Flores **M** *nepheronidesnepheronides* ♀ D NHM Indonesia: East Nusa Tenggara, Flores **N** *nepheronidestamborana* ♂ D OPC Indonesia: Sumbawa, Mt. Sengenges; Holotype.

Specimens: Figs 5A–N, 6A–P, 7A–O, 8A–H; Male Genitalia: Fig. 23A–K; Distribution: Fig. 40

**ssp. hypermnestra (Linnaeus, 1763)**

*Papilio hypermnestra* Linnaeus, 1763. **TL**: Java (Indonesia: Java). **TS**: LSL. *Amoenitates Acad.* 6: 407.

*Papilio protogenia* Cramer, 1779. **TL**: Java (Indonesia: Java). **TS**: NBC. *Uitl. Kapellen.* 2 (16): 141, pl. 189, fig. F–G.

*Hamadryas jynx* Hübner, 1808. **TL**: not indicated. **TS**: unknown. *Erste Zutr. Samml. exot. Schmett.* p. 4.

*Elymnias jynx* Hübner, 1818. **TL**: East Indies. **TS**: unknown. *Zuträge Samml. exot. Schmett.* 1: 12.

Elymnias hypermnestra hypermnestra f. perpusilla Fruhstorfer, 1907. **TL**: Java (Indonesia: Java). **TS**: NHM. *Dt. ent. Z. Iris* 20 (3): 181.

Elymnias hypermnestra f. atrata Roepke, 1942. **TL**: Java (Indonesia: Java). **TS**: NBC. *Rhop. Javan.* (4): 422.^18^

**ssp. undularis (Drury, 1773)**^19^

*Papilio undularis* Drury, 1773. **TL**: East Indies. **TS**: NHM. *Ill. Nat. Hist. Exot. Insects* 2: 17, pl. 10, f. 1–2.

*Biblis undularis* Westwood, 1837. **TL**: East Indies, Java (Indonesia: Java). **TS**: NHM. *Ill. Exo. Ent.* 2: 18, pl. X, figs 1–2.

*Melanitis undularis* Westwood, 1851. **TL**: East India, Java (Indonesia: Java). **TS**: NHM. *Gen. diurn. Lep.* 2: 404.

**ssp. fraterna Butler, 1871**

*Elymnias fraterna* Butler, 1871. **TL**: Ceylon (Sri Lanka). **TS**: NHM. *Proc. Zool. Soc. Lond.* 1871: 520, pl. 42, fig. 3.

**ssp. nigrescens Butler, 1871**

*Elymnias nigrescens* Butler, 1871. **TL**: Sarawak (East Malaysia: Sarawak). **TS**: NHM. *Proc. Zool. Soc. Lond.* 1871: 520, pl. 42, fig. 1.

*Elymnias hecate* Butler, 1871. **TL**: Labuan, Borneo (East Malaysia: Labuan). **TS**: NHM. *Proc. Zool. Soc. Lond.* 1871 (2): 520, pl. 42, f. 2.

Elymnias nigrescens nigrescens f. pseudagina Fruhstorfer, 1907. **TL**: Sarawak, Borneo (East Malaysia: Sarawak). **TS**: NHM. *Dt. ent. Z. Iris* 20 (3): 191.

Elymnias nigrescens nigrescens f. edela Fruhstorfer, 1907. **TL**: Pontianak (Indonesia: West Kalimantan, Pontianak). **TS**: NHM. *Dt. ent. Z. Iris* 20 (3): 191.

Elymnias nigrescens nigrescens f. virilis Fruhstorfer, 1907. **TL**: Lawas (East Malaysia: Sarawak, Lawas). **TS**: NHM. *Dt. ent. Z. Iris* 20 (3): 191.

Elymnias nigrescens nigrescens f. hecate Fruhstorfer, 1907. **TL**: Labuan (East Malaysia: Labuan). **TS**: NHM. *Dt. ent. Z. Iris* 20 (3): 191.

**ssp. cottonis (Hewitson, 1874). comb. n.**^20^

*Melanitis cottonis* Hewitson, 1874. **TL**: Andaman Islands (India: Andaman Islands). **TS**: NHM. *Ann. Mag. nat. Hist.* 14 (83): 358.

*Elymnias cottonis cottonis* Fruhstorfer, 1907. **TL**: Andaman Islands (India: Andaman Islands). **TS**: NHM. *Dt. ent. Z. Iris* 20 (3): 183.

**ssp. tinctoria Moore, [1879**]^21^

*Elymnias tinctoria* Moore, [1879]. **TL**: Meetan, Moolai (Myanmar: Tanintharyi) **TS**: NHM. *Proc. Zool. Soc. Lond.* 1878 (4): 826.

Elymnias hypermnestra tinctoria f. paraleuca Fruhstorfer, 1907. **TL**: Mergui-Archiel, Tenasserim (Myanmar: Thanintharyi, Mergui Archipelago). **TS**: NHM. *Dt. ent. Z. Iris* 20 (3): 177.

**ssp. hainana Moore, 1878**^22^

*Elymnias hainana* Moore, 1878. **TL**: Hainan (China: Hainan). **TS**: NHM. *Proc. zool. Soc. Lond.* 1878 (3): 696.

*Elymnias nigrescens formosana* Fruhstorfer, 1903. **TL**: Takau (Taiwan: Kaohsiung). **TS**: NHM. *Dt. ent. Z. Iris* 16 (1): 17.

*Elymnias nigrescens tonkiniana* Fruhstorfer, 1902. **syn. n. TL**: Tonkin, Haiphong (Vietnam: Haiphong). **TS**: NHM. *Dt. ent. Z. Iris* 14 (2): 271.

Elymnias hypermnestra nigrescens f. depicta Fruhstorfer, 1907. **syn. n. TL**: Tonkin (northern Vietnam). **TS**: NHM. *Dt. ent. Z. Iris* 20 (3): 188.

*Elymnias hypermnestra septentrionalis* Chou & Huang, 1994. **syn. n. TL**: Nanning (China: Guangxi, Nanning). **TS**: NWSUAF. *Monographia Rhopalocerum Sinensium* 1: 375, fig. 27.

**ssp. discrepans Distant, 1882**^23^

*Elymnias discrepans* Distant, 1882. **TL**: Penang, Province Wellesley (Peninsular Malaysia: Penang, Seberang Perai). **TS**: NHM or NHMT. *Ann. Mag. nat. Hist.* (5) 9 (53): 397.

**ssp. orientalis Röber, 1891**

*Elymnias orientalis* Röber, 1891. **TL**: Flores (Indonesia: East Nusa Tenggara, Flores). **TS**: unknown. *Tijdschr. Ent.* 34: 311.

*Elymnias nigrescens dohertyi* Fruhstorfer, 1902. **TL**: Ende Island (Indonesia: East Nusa Tenggara, Flores, Ende Island). **TS**: NHM. *Dt. ent. Z. Iris* 14 (2): 273.

**ssp. baliensis Fruhstorfer, 1896**

*Elymnias protegenia baliensis* Fruhstorfer, 1896. **TL**: Bali (Indonesia: Bali). **TS**: NHM. *Soc. Ent.*11 (18): 147.

*Elymnias nigrescens bulelenga* Rothschild, 1915. **TL**: Buleleng (Indonesia: Bali, Buleleng Regency). **TS**: NHM. *Novit. Zool.* 22 (1): 124.

**ssp. violetta Fruhstorfer, 1902**^19^

*Elymnias undularis violetta* Fruhstorfer, 1902. **TL**: Muok-Lek (Thailand: Saraburi, Muak Lek). **TS**: NHM. *Soc. Ent*. *Soc. Ent.* 16 (22):169.

Elymnias hypermnestra violetta f. epixantha Fruhstorfer, 1907. **TL**: Bangkok (Thailand: Bangkok). **TS**: NHM. *Dt. ent. Z. Iris* 20 (3):178.

Elymnias hypermnestra violetta f. obfuscata Riley, 1932. **TL**: Siam (Thailand). **TS**: NHM. *J. Siam. Soc.* 8 (4, Suppl.): 249.

**ssp. meridionalis Fruhstorfer, 1902**^19^

*Elymnias undularis meridionalis* Fruhstorfer, 1902. **TL**: south Annam (southern Vietnam). **TS**: NHM. *Soc. Ent.* 16 (22): 169.

Elymnias meridionalis f. orphnia, Fruhstorfer, 1907. **TL**: south Annam (southern Vietnam). **TS**: NHM. *Dt. ent. Z. Iris* 20 (3): 179.

**ssp. beatrice Fruhstorfer, 1902. comb. n.**^24^

*Elymnias nigrescens*, Distant, 1882. *Rhopalocera Malayana*: 61.

*Elymnias nigrescens beatrice* Fruhstorfer, 1902. **nomen n.** for Distant’s *nigrescens*. **TL**: Singapore, Perak (Peninsular Malaysia: Perak), Lingga (Indonesia: Riau Islands, Lingga Archipelago, Lingga Island), Deli, (Indonesia: North Sumatra Province, Deli Serdang Regency), Sumatra (Indonesia: Sumatra), Wellesley Province (Peninsular Malaysia: Penang, Seberang Perai), Billiton (Indonesia: Bangka-Belitung Province, Belitung). **TS**: NHM. *Dt. ent. Z. Iris* 14 (2): 272.^22^

Elymnias nigrescens ab. agina Fruhstorfer, 1902. **unavailable name. TL**: Singapore, Sumatra (Indonesia: Sumatra), Perak (Peninsular Malaysia: Perak). **TS**: NHM. *Dt. ent. Z. Iris* 14 (2): 272.^22^

Elymnias nigrescens beatrice f. ornamenta Fruhsorfer, 1907. **unavailable name. TL**: Malay (Peninsular Malaysia). **TS**: NHM. *Dt. ent. Z. Iris* 20 (3): 190.^22^

*Elymnias hypermnestra agina*, Corbet, 1943. *Proc. Roy. Ent. Soc. Lond.* (B) 12: 117–119.

**ssp. sumbana Fruhstorfer, 1902**^25^

*Elymnias nigrescens sumbana* Fruhstorfer, 1902**. TL**: Sumba (Indonesia: East Nusa Tenggara, Sumba). **TS**: NHM. *Dt. ent. Z. Iris* 14 (2): 273.

**ssp. decolorata Fruhstorfer, 1907**

Elymnias nigrescens beatrice forma decolorata Fruhstorfer, 1907. **unavailable name. TL**: Sumatra (Indonesia: Sumatra). **TS**: NHM. *Dt. ent. Z. Iris* 20 (3): 189.

*Elymnias hypermnestra decolorata*, Aoki, Yamaguchi & Uémura, 1982. *Butterflies of the Southeast Asian Islands* 3: 175–176.

**ssp. sumbawana Fruhstorfer, 1907**

*Elymnias nigrescens sumbawana* Fruhstorfer, 1907. **TL**: Tambora, Sumbawa (Indonesia: West Nusa Tenggara, Sumbawa, Mt. Tambora). **TS**: NHM. *Dt. ent. Z. Iris* 20 (3): 197.

**ssp. timorensis Fruhstorfer, 1907**

*Elymnias nigrescens timorensis* Fruhstorfer, 1907. **TL**: Timor. **TS**: NHM. *Dt. ent. Z. Iris* 20 (3): 198.

**ssp. alorensis Talbot, 1932**

*Elymnias nigrescens alorensis* Talbot, 1932. **TL**: Alor (Indonesia: East Nusa Tenggara, Alor). **TS**: NHM. *Bull. Hill Mus. Witley* 4: 167.

**ssp. nimota Corbet, 1937**

*Elymnias hypermnestra nimota* Corbet, 1937. **TL**: Tioman (Peninsular Malaysia: Pahang, Rompin, Tioman Island). **TS**: NHM. *Proc. R. ent. Soc. Lond.* 6 (5): 97.

**ssp. kangeana Aoki & Uémura, 1982**

*Elymnias hypermnestra kangeana* Aoki & Uémura, 1982. **TL**: Kangean (Indonesia: East Java, Sumenap Regency, Kangean). **TS**: TPC. *Mem. Tsukada Coll.* 4: 2.

**ssp. robinsona Monastyrskii & Devyatkin, 2003**

*Elymnias hypermnestra robinsona* Monastyrskii & Devyatkin, 2003. **TL**: Con Dao, Con Son Island (Vietnam: Ba Ria–Vung Tau Province, Con Dao Archipelago, Con Son Island). **TS**: NHM. *Atalanta* 34 (1/2): 81, pl. 5, figs 5, 7–8.

**ssp. jennifferae Suzuki, 2006. comb. n.**

*Elymnias cottonis jennifferae* Suzuki, 2006. **TL**: Little Andaman (India: Andaman Islands, Little Andaman Island). **TS**: KMSPC. *Futao* (52): 13.

**ssp. uemurai Lamas, 2010** (replaced *Elymnias nigrescens meliophila* Fruhstorfer, 1896a). *Shilap* 38 (150): 198.

*Elymnias nigrescens meliophila* Fruhstorfer, 1896a. **TL**: Lombok (Indonesia: West Nusa Tenggara, Lombok). **TS**: NHM. *Soc. Ent.*11 (18): 147. (preoccupied by *Elymnias hewitsoni meliophila* Fruhstorfer 1896b).

***caudata* Butler, 1871**^26^

Specimens: Fig. 8J–K; Male Genitalia: Fig. 23L; Distribution: Fig. 41

*Elymnias caudata* Butler, 1871. **TL**: Canara (India: Karnataka, Kanara). **TS**: NHM. *Proc. Zool. Soc. Lond.* 1871: 520, pl. 42, fig. 4.

***merula* Swinhoe, 1915. *incertae sedis***^27^

Specimen: Fig. 8K; Distribution: Fig. 42

*Elymnias merula* Swinhoe, 1915. **TL**: Kandy, Ceylon (Sri Lanka: Central Province, Kandy). **TS**: NHM. *Ann. Mag. nat. Hist.* 16 (93): 171.

***leucocyma* Godart, 1819. *incertae sedis***^28^

Distribution: Fig. 43

*Biblis leucocyma* Godart, 1819. **TL**: Java (Indonesia: Java). **TS**: unknown. *Encyc. Méth.* 9: 326.

### *nepheronides*-group

***nepheronides* Fruhstorfer, 1907**^29^

Specimens: Fig. 8L–N; Male Genitalia: Fig. 23M; Distribution: Fig. 44

**ssp. nepheronides Fruhstoerfer, 1907**

*Elymnias nepheronides* Fruhstorfer, 1907. **TL**: Flores Island (Indonesia: East Nusa Tenggara, Flores). **TS**: NHM. *Dt. ent. Z. Iris* 20 (3): 228.

*Elymnias detanii* Aoki & Uémura, 1982. **TL**: Flores (Indonesia: East Nusa Tenggara, Flores). **TS**: NODAI. *Butterflies of the Southeast Asian Islands* 3: 208.

**ssp. tamborana Okubo, 2010**

*Elymnias tamborana* Okubo, 2010. **TL**: Mt. Ngegep, Sumbawa (Indonesia: West Nusa Tenggara, Sumbawa, Mt. Sengenges). **TS**: OPC. *Trans. Lep. Soc. Jpn.* 60 (4): 255–257.

### *harterti*-group

***harterti* Honrath, 1889**^30^

**Figure 9.**
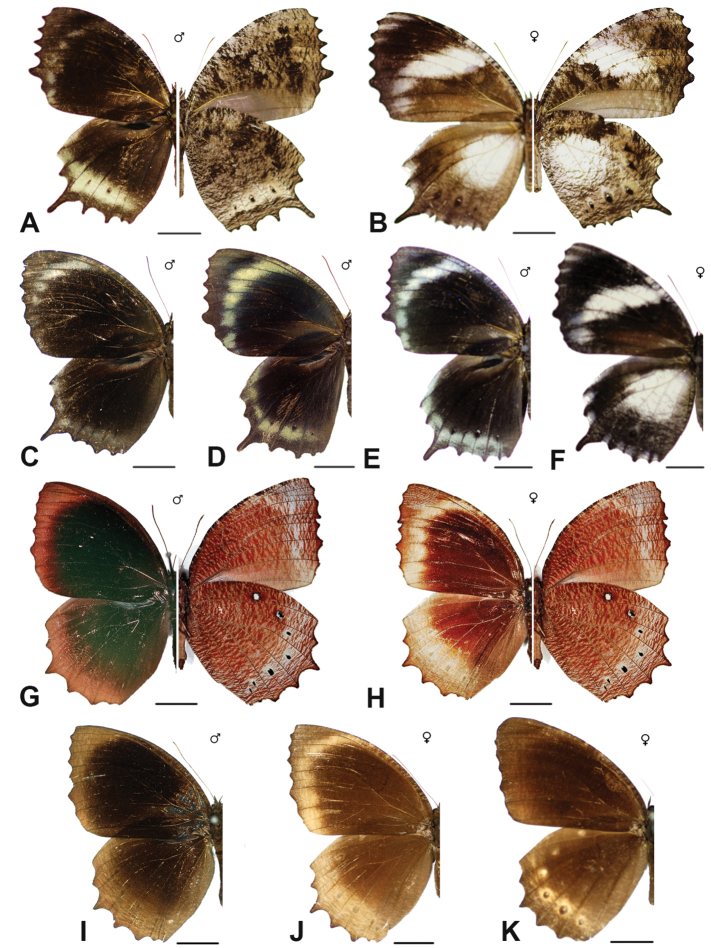
**A** *hartertiharterti* ♂ D+V OPC Malaysia: Perak, Batang Padang, Tapah **B** *hartertiharterti* ♀ D+V OPC Malaysia: Perak, Batang Padang, Tapah **C** *hartertibrookei* ♂ D NHM collection locality unknown **D** *hartertilautensis* ♂ D OPC Indonesia: South Kalimantan, Laut Island; Holotype **E** *hartertiarbaimuni* ♂ D OPC Indonesia: central Sumatra, Jambi; Holotype **F** *hartertiarbaimuni* ♀ D OPC Indonesia: central Sumatra, Jambi **G** *parcejustini* ♂ D+V SMFD Philippines: Palawan Province, Calamian Islands, Busuanga Island, Coron; Holotype **H** *parcejustini* ♀ D+V SMFD Philippines: Palawan Province, Calamian Islands, Busuanga Island, Coron; Paratype **I** *parceparce* ♂ D NHM Philippines: Palawan **J** *parceparce* ♀ D NHM Philippines: Palawan **K** *pantheraenganica* ♀ D NHM Indonesia: Bengkulu, Enggano Island.

Specimens: Fig. 9A–F; Distribution: Fig. 45

**ssp. harterti Honrath, 1889**

*Elymnias harterti* Honrath, 1889. **TL**: Perak (Peninsular Malaysia: Perak). **TS**: NHM. *Berl. ent. Z.* 33 (1): 165.

**ssp. brookei Shelford, 1904**

*Elymnias brookei* Shelford, 1904**. TL**: Sarawak (East Malaysia: Sarawak). **TS**: NHM. *J. Straits Asiat. Soc.* (41): 102.

*Elymnias smithi* Moulton, 1915. **syn. n. TL**: Mt. Molu (East Malaysia: Sarawak, Mt. Molu). **TS**: NHM. *Entomologist* 48: 98.

**ssp. lautensis Medicielo & Hanafusa, 1994**

*Elymnias harterti lautensis* Medicielo & Hanafusa, 1994. **TL**: Laut Island (Indonesia: South Kalimantan, Kota Baru Regency, Laut Island). **TS**: HPC. *Futao* (15): 17, pl. 4, figs 17–18.

**ssp. arbaimuni Hanfusa, 2005**

*Elymnias haterti* [sic] *arbaimuni* Hanfusa, 2005. **TL**: Indonesia: Jambi Province, Kuala Tungkal, Suban. **TS**: HPC. *Futao* (49): 11, pl. 1, figs 11–12.

***parce* Staudinger, 1889**

Specimens: Fig. 9G–J; Male Genitalia: Fig. 23N; Distribution: Fig. 46

**ssp. parce Staudinger, 1889**^31^

*Elymnias panthera parce* Saudinger, 1889. **TL**: Palawan (Philippines: Palawan). **TS**: ZMHB. *Dt. ent. Z. Iris* 2 (1): 39.

**ssp. justini Schröder & Treadaway, 2003**

*Elymnias parce justini* Schröder & Treadaway, 2003. **TL**: Philippines: Palawan, Busuanga Island. **TS**: SMFD. *Nachr. ent. Ver. Apollo* 23 (4): 194, pl. 1, fig. 21.

### *panthera*-group

***panthera* (Fabricius, 1787)**

**Figure 10.**
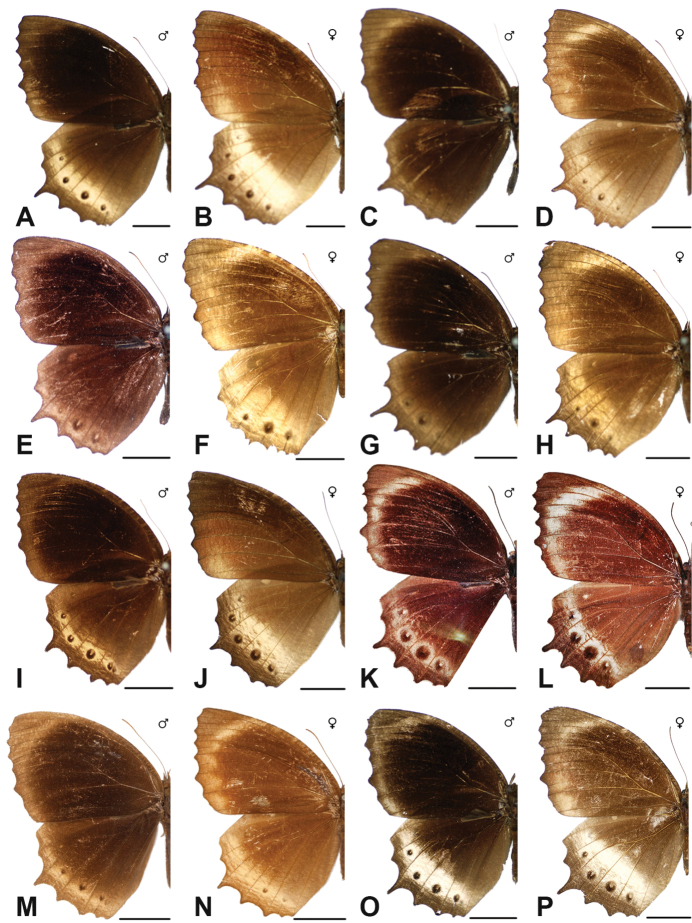
**A** *pantherapanthera* ♂ D NHM Peninsular Malaysia **B** *pantherapanthera* ♀ D NHM Peninsular Malaysia **C** *pantheradusara* ♂ D NHM Indonesia: Java **D** *pantheradusara* ♀ D NHM Indonesia: Java **E** *pantheramimus* ♂ D NHM India: Nicobar Islands **F** *pantheramimus* ♀ D NHM India: Nicobar Islands **G** *pantheradolorosa* ♂ D NHM Indonesia: North Sumatra, Nias **H** *pantheradolorosa* ♀ D NHM Indonesia: North Sumatra, Nias **I** *pantheralutescens* ♂ D NHM North Borneo **J** *pantheralutescens* ♀ D NHM East Malaysia: Sarawak **K** *pantherasuluana* ♂ D SMFD collection locality unknown **L** *pantherasuluana* ♀ D SMFD Philippines: Tawi-tawi, Mapun Island **M** *pantheratautra* ♂ D NHM Indonesia: Sumatra, Bengkalis, Senggoro **N** *pantheratautra* ♀ D NHM Indonesia: Sumatra, Bengkalis, Senggoro **O** *pantheraarikata* ♂ D NHM Indonesia: Riau Islands, Natuna **P** *pantheraarikata* ♀ D NHM Indonesia: Riau Islands, Natuna.

Specimens: Figs 9K, 10A–P; Male Genitalia: Figs 23O, 24A; Distribution: Fig. 47

**ssp. panthera (Fabricius, 1787)**^32^

*Papilio panthera* Fabricius, 1787. **TL**: Tranquebariae (India: Tamil Nadu, Tharangambadi). **TS**: ZMUC. *Mantissa Ins.* 2: 39.

**ssp. dusara (Horsfield, [1829])**^33^

*Melanitis dusara* Horsfield, [1829]. **TL**: West Java (Indonesia: West Java). **TS**: NHM. *Descr. Cat. lep. Ins. Mus. East India Coy.* 2: pl. 5, f. 7.

**ssp. mimus Wood-Mason & de Nicéville, 1881**

*Elymnias mimus* Wood-Mason & de Nicéville, 1881. **TL**: Nicobar Islands (India: Nicobar Islands). **TS**: uknown. *J. Asiat. Soc. Bengal* 50: 230.

**ssp. dolorosa Butler, 1883**.

*Elymnias dolorosa* Butler, 1883. **TL**: Nias Island (Indonesia: North Sumatra, Indonesia, Nias). **TS**: NHM. *Ent. mon. Mag.* 20: 53.

**ssp. lutescens Butler, 1867. comb. n., stat. n.**^34^

*Elymnias lutescens* Butler, 1867. **TL**: Malacca, Singapore and Penang (Singapore & Peninsular Malaysia: Penang and Malacca). **TS**: NHM. *Ann. Mag. nat. Hist.* 20 (120): 404, pl. 9, f. 10.

Elymnias panthera var. labuana Staudinger, 1889. **syn. n. TL**: Labuan Island (East Malaysia: Labuan). **TS**: ZMHB. *Dt. ent. Z. Iris* 2 (1): 39.

*Elymnias panthera lacrima* Fruhstorfer, 1904. **syn. n. TL**: [North Borneo], [Banka] (Indonesia: Banka-Belitung Province, Banka Island). **TS**: NHM. *Berl. ent. Zs.* 49: 188.

*Elymnias defasciata* Fruhstorfer, 1911, **syn. n. TL**: Borneo. **TS**: **TS**: NHM. *Gross-Schmett. Erde* 9: 372.

*Elymnias panthera alfredi* Fruhstorfer, 1907. **syn. n. TL**: Southeast Borneo. **TS**: NHM. *Dt. ent. Z. Iris* 20 (3): 220.

Elymnias panthera alfredi f. pantherina Fruhstorfer, 1907. **unavailable name. TL**: Southeast Borneo. **TS**: NHM. *Dt. ent. Z. Iris* 20 (3): 220.

Elymnias panthera alfredi f. alfredi Fruhstorfer, 1907. **unavailable name. TL**: Southeast Borneo. **TS**: NHM. *Dt. ent. Z. Iris* 20 (3): 220.

**ssp. enganica Doherty, 1891**

*Elymnias enganica* Doherty, 1891. **TL**: Engano (Indonesia: Bengkulu, Enggano Island). **TS**: NHM. *J. Asiat. Soc. Bengal. Part 2*, 60 (1): 24.

**ssp. lacrimosa Fruhstorfer, 1898**

*Elymnias panthera lacrimosa* Fruhstorfer, 1898. **TL**: Bawean Island (Indonesia: East Java, Gresik Regency, Bawean). **TS**: NHM. *Berl. ent. Zs.* 43: 196.

**ssp. suluana Fruhstorfer, 1899**

*Elymnais panthera suluana* Fruhstorfer, 1899. **TL**: Sulu Island (Philippines: Sulu Province, Sulu Island). **TS**: NHM. *Berl. ent. Zs.* 44: 57.

**ssp. bangueyana Fruhstorfer, 1899**

*Elymnias panthera bangueyana* Fruhstorfer, 1899. **TL**: Banguey Island (Malaysia: Sabah, Banggi Island). **TS**: NHM. *Berl. ent. Zs.* 44: 58.

**ssp. dulcibella Fruhstorfer, 1907**

Elymnias panthera f. dulcibella Fruhstorfer, 1907. **TL**: East Java (Indonesia: East Java). **TS**: NHM *Dt. ent. Z. Iris* 20 (3): 223.^31^

**ssp. tautra Fruhstorfer, 1907**

*Elymnias panthera tautra* Fruhstorfer, 1907. **TL**: Northeast Sumatra (Indonesia: North Sumatra). **TS**: NHM. *Dt. ent. Z. Iris* 20 (3): 218 (repl. *E. lutescens* Martin & de Nicéville, 1896)

**ssp. arikata Fruhstorfer, 1907**

*Elymnias panthera arikata* Fruhstorfer, 1907. **TL**: Natuna Island (Indonesia: Riau Province, Natuna Island). **TS**: NHM. *Dt. ent. Z. Iris* 20 (3): 219.

**ssp. balina Martin, 1909**

*Elymnias panthera balina* Martin, 1909. **TL**: Bali Island (Indonesia: Bali). **TS**: NHMT. *Dt. ent. Z. Iris* 22 (1): 58.

**ssp. exsulata van Eecke, 1918**

*Elymnias panthera exsulata* van Eecke, 1918. **TL**: Pulu [sic] Lasia (Indonesia: North Sumatra, Lasia Island). **TS**: NBC. *Zoologische Mededeelingen* 4 (2): 82.

**ssp. winkleri Kalis, 1933**

*Elymnias panthera winkleri* Kalis, 1933. **TL**: Sabang, Weh Island (Indonesia: Aceh, Sabang, Weh Island). **TS**: MEPR. *Tijdschrift voor Entomologie* 76 (1–2): 80.

**ssp. mira Corbet, 1942**

*Elymnias panthera mira* Corbet, 1942. **TL**: Sipora Island (Indonesia: West Sumatra, Mentawai Regency, Sipora). **TS**: NHM. *Ann. Mag. nat. Hist.* (11) 9 (56): 612.

**ssp. tiomanica Eliot, 1978**

*Elymnias panthera tiomanica* Eliot, 1978. **TL**: Tioman (Peninsular Malaysia: Pahang, Rompin, Tioman Island). **TS**: NHM. *Butterflies of the Malay Peninsula*, 3^rd^ ed: 413.

**ssp. belitungensis Okano, 1986**

*Elymnias panthera belitungensis* Okano, 1986. **TL**: Belitung Island (Indonesia: Bangka-Belitung Province, Belitung). **TS**: OMPC. *Tokurana* 11 (1): 1, figs 1–6.

**ssp. ruricolaris Hanafusa, 1989**

*Elymnias panthera ruricolaris* Hanafusa, 1989. **TL**: Karimata Island (Indonesia: West Kalimatan Province, Karimata Island). **TS**: HPC. *Futao* (3): 10, pl. 3, figs 1–4.

**ssp. banyakensis Hanafusa, 1993**

*Elymnias panthera banyakensis* Hanafusa, 1993. **TL**: Kepulauan Banyak (Indonesia: Aceh, Banyak Islands). **TS**: HPC. *Futao* (13): 8.

**ssp. attenuata Hanafusa, 1994**

*Elymnias panthera attenuata* Hanafusa, 1994. **TL**: Tanahmasa Island (Indonesia: North Sumatra Province, Tanahmasa Island). **TS**: HPC. *Futao* (4): 13.

**ssp. redangensis Hanafusa, 2001**

*Elymnias panthera redangensis* Hanafusa, 2001. **TL**: Redang Island (Peninsular Malaysia: Terengganu, Redang Island). **TS**: HPC. *Futao* (37): 14, pl.1, figs 5–8.

**ssp. zeta Abang, Treadaway & Schröder, 2004**

*Elymnias panthera zeta* Abang, Treadaway & Schröder, 2004. **TL**: Balambangan Island (East Malaysia: Sabah, Balambangan Island). **TS**: MUS. *Futao* (47): 10, pl. 3, figs 33–36.

***obnubila* Marshall & de Nicéville, 1883**

**Figure 11.**
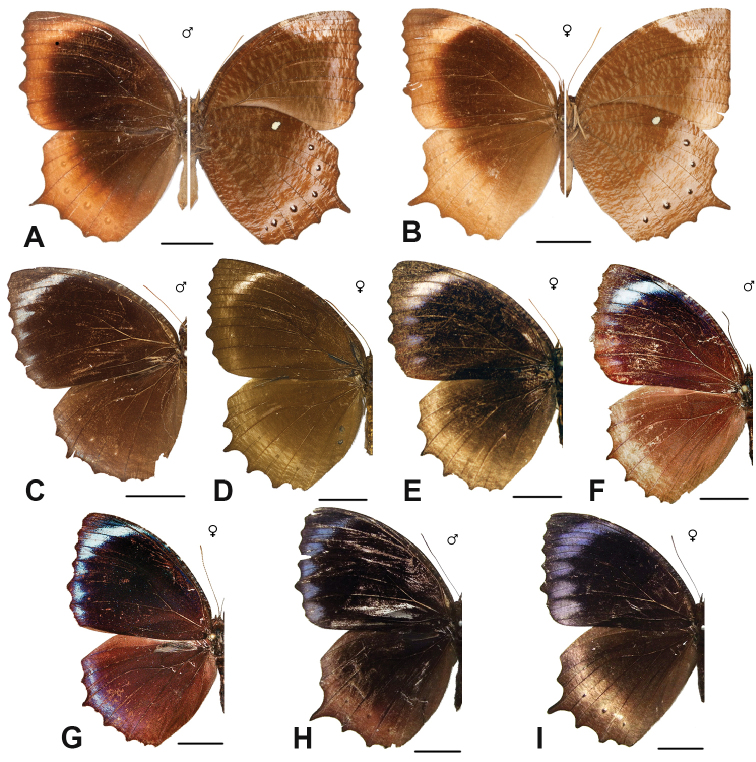
**A** *obnubila* ♂ D+V NHM Malaysia: Perak **B** *obnubila* ♀ D+V NHM Thailand: Ranong **C** *congruenscongruens* ♂ D NHM Philippines: Cebu, Camotes Island **D** *congruenssubcongruens* ♀ D NHM Philippines: Mindoro **E** *congruensendida* ♂ D SMFD Philippines: Bohol **F** *congruensendida* ♀ D SMFD Philippines: Bohol **G** *congruenscongruens* ♀ D NMNH Philippines: Mindanao, Davao **H** *miyagawai* ♂ D SPC Vietnam: Lam Dong, Loc Bao; Holotype **I** *miyagawai* ♀ D SPC Vietnam: Lam Dong, Loc Bao; Paratype.

Specimens: Fig. 11A–B; Male Genitalia: Fig. 24B; Distribution: Fig. 48

*Elymnias obnubila* Marshall & de Nicéville, 1883. **TL**: Mergui (Myanmar: Thanintharyi, Mergui Archipelago). **TS**: IM. *Butts India Burmah Ceylon* 1 (2): 272.

***congruens* Semper, 1887**

Specimens: Fig. 11C–G; Male Genitalia: Fig. 24C; Distribution: Fig. 49

**ssp. congruens Semper, 1887**^35^

*Elymnias congruens* Semper, 1887. **TL**: N. Mindanao (Philippines: northern Mindanao). **TS**: SMFD. *Reisen. Philipp.* 2: 61, pl. 11, fig. 8–10.

*Elymnias congruens photinus* Fruhstorfer, 1907. **syn. n. TL**: N. Mindanao (Philippines: northern Mindanao). **TS**: NHM. *Dt. ent. Z. Iris* 20 (3): 199.

*Elymnias congruens phaios* Fruhstorfer, 1907. **syn. n. TL**: S. Mindanao (Philippines: southern Mindanao). **TS**: NHM. *Dt. ent. Z. Iris* 20 (3): 200.

*Elymnias congruens rafaela* Fruhstorfer, 1907. **syn. n. TL**: Bazilan (Philippines: Sulu Archipelago, Basilan). **TS**: NHM. *Dt. ent. Z. Iris* 20 (3): 200.

**ssp. subcongruens Semper, 1892**

*Elymnias subcongruens* Semper, 1892. **TL**: Mindoro (Philippines: Mindoro). **TS**: SMFD. *Reisen. Philipp.* 7: 329.

**ssp. endida Fruhstorfer, 1911**

*Elymnias congruens endida* Fruhstorfer, 1911. **TL**: Bohol (Philippines: Bohol). **TS**: NHM. *Gross-Schmett. Erde* 9: 379.

**ssp. salipi Schroeder & Treadaway, 1989**

*Elymnias salipi* Schroeder & Treadaway, 1989. **TL**: Philippines: Tawi-Tawi Archipelago, Sanga Sanga Island, Boloboc. **TS**: SMFD. *Ent. Z.* 99 (22): 327, fig. 6.

**ssp. jekei Schroeder & Treadaway, 1989**

*Elymnias jekei* Schroeder & Treadaway, 1989. **TL**: Philippines: Luzon, Nueva Ecija, near Carranglan. **TS**: SMFD. *Ent. Z.* 99 (22): 328, fig. 6.

**ssp. neergaardorum Schroeder & Treadaway, 2003**

*Elymnias neergaardorum* Schroeder & Treadaway, 2003. **TL**: Phillipines: Masbate. **TS**: SMFD. *Nachr. ent. Ver. Apollo* 23 (4): 194, pl. 1, figs 14–15.

***miyagawai* Saito & Kishi, 2012**

Specimens: Fig. 11H–I; Distribution: Fig. 50

*Elymnias miyagawai* Saito & Kishi, 2012. **TL**: Vietnam: Lam Dong. **TS**: SPC. *Butterflies* (62): 4, figs 1–2, 10.

### *nesaea*-group

***nesaea* (Linnaeus, 1764)**

**Figure 12.**
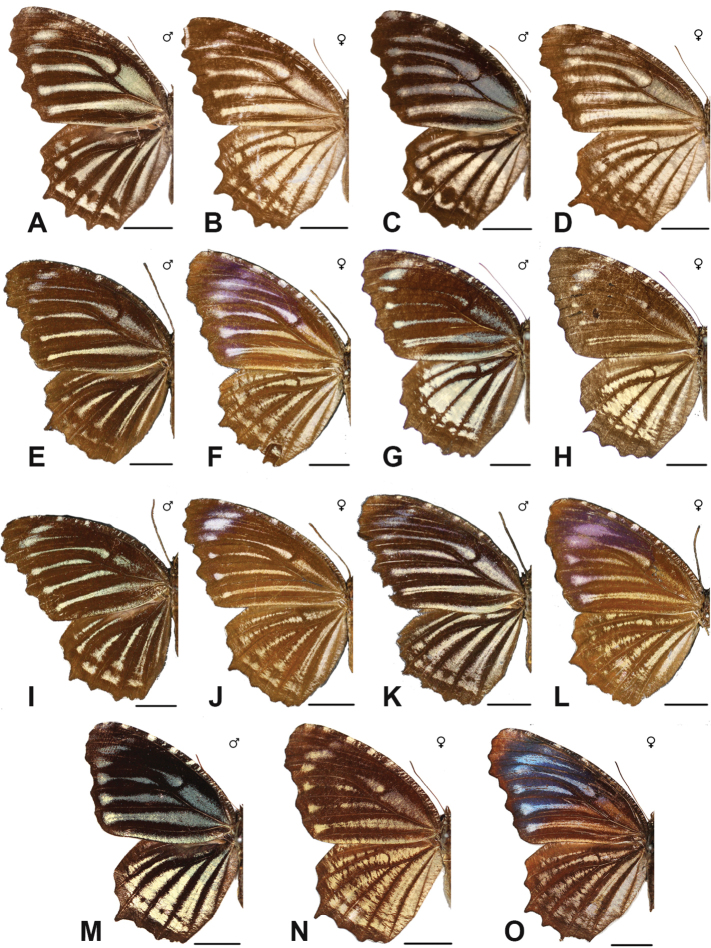
**A** *nesaeanesaea* ♂ D NHM Indonesia: Java **B** *nesaeanesaea* ♀ D NHM Indonesia: Java **C** *nesaeatimandra* ♂ D NHM India: Meghalaya, Khasi Hills **D** *nesaeatimandra* ♀ D NHM India: Meghalaya, Khasi Hills **E** *nesaealaisidis* ♂ D MCZ Indonesia: Sumatra **F** *nesaealaisidis* ♀ D MCZ Indonesia: West Sumatra, Padang **G** *nesaeabaweana* ♂ D NHM Indonesia: East Java, Gresik Regency, Bawean **H** *nesaea baewana* ♀ D NHM Indonesia: East Java, Gresik Regency, Bawean **I** *nesaeaneolais* ♂ D MCZ Indonesia: North Sumatra, Nias, Dymna **J** *nesaeaneolais* ♀ D MCZ Indonesia: North Sumatra, Nias **K** *nesaeahypereides* ♂ D MCZ East Malaysia: Sabah, Sandakan **L** *nesaeahypereides* ♀ D MCZ East Malaysia: Sabah, Sandakan **M** *nesaeaapelles* ♂ D KUTH Thailand: Samut Sakhon **N** *nesaeaapelles* ♀ D KUTH Thailand: Bangkok, Bang Khen **O** *nesaealioneli* ♀ D KUTH Thailand: Satun, Thale Ban.

Specimens: Fig. 12A–O; Male Genitalia: Fig. 24D–G; Distribution: Fig. 51

**ssp. nesaea (Linnaeus, 1764)**^36^

Papilio (Nymphalis) nesaea Linnaeus, 1764. **TL**: [Java] (Indonesia: Java). **TS**: LSL. *Mus. Lud. Ulr. Reg.*: 302.

*Papilio lais* Cramer, 1777. **TL**: Java (Indonesia: Java). **TS**: unknown. *Uitl. Kapellen* 2 (10): 21, pl. 110, f. A–B.

*Elymnias nesaea hermia* Fruhstorfer, 1907. **syn. n. TL**: near Lawang, (Indonesia: East Java, Lawang). **TS**: NHM. *Dt. ent. Z. Iris* 20 (3): 206.^34^

**ssp. timandra Wallace, 1869**^37^

*Elymnias timandra* Wallace, 1869. **TL**: Sylhet (Bangladesh: Sylhet Division), Moulmein (Myanmar: Mon State, Mawlamyine). **TS**: NHM. *Trans. ent. Soc. Lond.* 1869 (4): 326.

*Elymnias nesaea cortona* Fruhstorfer, 1911. **syn. n. TL**: Burma (Myanmar). **TS**: NHM. *Gross-Schmett. Erde* 9: 379.

**ssp. laisidis de Nicéville, 1896**

Elymnias (Melynias) laisidis de Nicéville, 1896. **TL**: Sumatra (Indonesia: Sumatra). **TS**: IM. *J. Asiat. Soc. Bengal*, *Part 2*, 64 (3): 390.

**ssp. baweana Hagen, 1896**

*Elymnias baweana* Hagen, 1896. **TL**: Bawean Island (Indonesia: East Java, Gresik, Bawean). **TS**: NHMT. *Jahrb. Nass. Nat.* 49: 184, pl. 4, fig. 6.

**ssp. neolais de Nicéville, 1898**

Elymnias (Melynias) neolais de Nicéville, 1898. **TL**: Nias Island (Indonesia: North Sumatra, Nias). **TS**: IM. *J. Bomb. nat. Hist. Soc.* 12 (1): 136, pl. X, fig. 6.

**ssp. apelles Fruhstorfer, 1902**

*Elymnias lais apelles* Fruhstorfer, 1902. **TL**: Bangkok (Thailand: Bangkok). **TS**: NHM. *Soc. Ent.* 16 (22): 169

**ssp. vordemani Snellen van Vollenhoven, 1902**

*Elymnias vordemani* Snellen van Vollenhoven, 1902. **TL**: Kangean Island (Indonesia: East Java, Sumenap, Kangean). **TS**: NBC. *Tijdschr. Ent.* 45: 77, pl. 8, fig. 1.

**ssp. hypereides Fruhstorfer, 1903** ^36^

*Elymnias lais hypereides* Fruhstorfer, 1903. **TL**: North Borneo. **TS**: NHM. *Dt. ent. Z. Iris* 15 (2): 315

*Elymnias nesaea coelifrons* Fruhstorfer, 1907. **syn. n. TL**: Southeast Borneo (Indonesia: South or East Kalimantan). **TS**: NHM. *Dt. ent. Z. Iris* 20 (3): 205.^38^

**ssp. kamarina Fruhstorfer, 1906**

*Elymnias lais kamarina* Fruhstorfer, 1906. **TL**: Batu Island (Indonesia: North Sumatra, South Nias Regency, Batu Islands). **TS**: NHM. *Ent Zs.* 20 (15): 98.

**ssp. lioneli Fruhstorfer, 1907**

*Elymnias nesaea lioneli* Fruhstorfer, 1907. **TL**: Malaysia. **TS**: NHM. *Dt. ent. Z. Iris* 20 (3): 203.

**ssp. tawicola Schröder & Treadaway, 1989**

*Elymnias nesaea tawicola* Schröder & Treadaway, 1989. **TL**: Philippines: Tawi-Tawi Archipelago, Sibutu Island, Cavan Cavan. **TS**: SMFD. *Ent. Z.* 99 (22): 326, fig. 4.

***casiphone* Geyer, [1827**]

Specimens: Fig. 12A–M; Male Genitalia: Fig. 24H–L; Distribution: Fig. 52

**ssp. casiphone Geyer, [1827**]

*Elymnias casiphone* Geyer, [1827]. **TL**: not indicated. **TS**: unknown. *Samml. exot. Schmett.* 3: pl. [9], f. 1–2.^39^^40^

*Elymnias kamara* Moore, [1858]. **syn. n. TL**: Java (Indonesia: Java). **TS**: NHM. *Cat. lep. Ins. Mus. East India Coy* 1: 239.

*Elymnias kamara pareuploea* Fruhstorfer, 1911. **TL**: [Java] (Indonesia: Java). **TS**: NHM. *Gross-Schmett. Erde* 9: 382, pl. 87e.

*Elymnias kamara pseudalumna* Fruhstorfer, 1911. **TL**: Java (Indonesia: Java). **TS**: NHM. *Gross-Schmett. Erde* 9: 382.

**ssp. erinyes de Nicéville, 1895. comb. rev.**^41^

Elymnias (Melynias) erinyes de Nicéville, 1895. **TL**: Battak Mountains (Indonesia: North Sumatra). **TS**: IM. *J. Bomb. nat. Hist. Soc.* 10 (1): 19, pl. R, figs 9–10.

**ssp. praetextata Fruhstorfer, 1896**

*Elymnias casiphone praetextata* Fruhstorfer, 1896. **TL**: Lombok (Indonesia: West Nusa Tenggara, Lombok). **TS**: NHM. *Soc. Ent.* 11 (17): 140.^42^

*Elymnias kamara lombokiana* Fruhstorfer, 1911. **syn. n. TL**: Lombok Island (Indonesia: West Nusa Tenggara, Lombok). **TS**: NHM. *Gross-Schmett. Erde* 9: 383.^38^

**ssp. exclusa de Nicéville, 1898. comb. n.**

Elymnias (Melynias) exclusa de Nicéville, 1898. **TL**: Bali (Indonesia: Bali). **TS**: IM. *J. Asiat. Soc. Bengal, Part II* 66 (4): 681.^43^

*Elymnias casiphone djilantik* Martin, 1909, **syn. n. TL**: Bali (Indonesia: Bali). **TS**: NHMT. *Dt. ent. Z. Iris* 22 (1): 49.^41^

**ssp. alumna Fruhstorfer, 1907**

*Elymnias casiphone alumna* Fruhstorfer, 1907. **TL**: East Java (Indonesia: East Java). **TS**: NHM. *Dt. ent. Z. Iris* 20 (3): 209.

***malelas* (Hewitson, 1863)**

Specimens: Fig. 14A–D; Male Genitalia: Fig. 24M; Distribution: Fig. 53

**ssp. malelas (Hewitson, 1863)**^44^

*Melanitis malelas* Hewitson, 1863. **TL**: East India. **TS**: NHM. *Ill. exot. Butts.* 4: [70], pl. [36], f. 6–7.

Elymnias malelas malelas ab. subdecorata Fruhstorfer, 1911. **unavailable name. TL**: Assam (India: Meghalaya). **TS**: NHM. *Gross-Schmett. Erde* 9: 381.

*Elymnias malelas ivena* Fruhstorfer, 1911. **syn. n. TL**: Thailand, N. Vietnam. **TS**: NHM. *Gross-Schmett. Erde* 9: 381.

*Elymnias malelas nilamba* Fruhstorfer, 1911. **syn. n. TL**: India. **TS**: NHM. *Gross-Schmett. Erde* 9: 381.

***saueri* Distant, 1882**^45^

Specimens: Fig. 14E–F; Distribution: Fig. 54

**ssp. saueri Distant, 1882**

*Elymnias saueri* Distant, 1882. **TL**: Malaysia, Province Wellesley (Peninsular Malaysia: Penang, Seberang Perai). **TS**: NHM. *Rhopalocera Malayana* p. 65, pl. 9, fig. 3.

***kochi* Semper, 1887**^46^

Specimens: Fig. 14G–H; Male Genitalia: Fig. 24N; Distribution: Fig. 55

**ssp. kochi Semper, 1887**

*Elymnias kochi* Semper, 1887. **TL**: Philippines: Central Luzon. **TS**: SMFD. *Reisen Philipp.* (2) 55: 63, pl. 12, fig. 4.

***casiphonides* Semper, 1892**^47^

Specimens: Fig. 14I–J; Male Genitalia: Fig. 24O; Distribution: Fig. 56

**ssp. casiphonides Semper, 1892**

*Elymnias casiphonides* Semper, 1892. **TL**: Philippines: Mindanao. **TS**: SMFD. *Reisen Philipp.* (7): 330.

**ssp. sanrafaela Schröder & Treadaway, 1980**

*Elymnias casiphonides sanrafaela* Schröder & Treadaway, 1980. **TL**: Philippines: Samar, San Rafael **TS**: SMFD. *Ent. Z.* 90 (21): 238, fig. 3.

***nelsoni* Corbet, 1942**

Specimens: Fig. 14K–L; Male Genitalia: Fig. 25A; Distribution: Fig. 57

**ssp. nelsoni Corbet, 1942**

*Elymnias nelsoni* Corbet, 1942. **TL**: Mentawei Islands (Indonesia: West Sumatra, Mentawai Islands). **TS**: NHM. *Ann. Mag. nat. Hist.* (11) 9 (56): 612, fig. 5.

***amoena* Tsukada & Nishiyama, 1979**

Specimens: Fig. 14M; Distribution: Fig. 58

**ssp. amoena Tsukada & Nishiyama, 1979**

*Elymnias amoena* Tsukada & Nishiyama, 1979. **TL**: Sumba (Indonesia: East Nusa Tenggara, Sumba). **TS**: TPC. *Mem. Tsukada Coll.* 1: 15, figs 19–20.

***kanekoi* Tsukada & Nishiyama, 1980**

Specimens: Fig. 14N–O; Male Genitalia: Fig. 25B; Distribution: Fig. 59

**ssp. kanekoi Tsukada & Nishiyama, 1980**

*Elymnias kanekoi* Tsukada & Nishiyama, 1980. **TL**: north Negros (Philippines: Negros Occidental). **TS**: TPC. *Mem. Tsukada Coll.* 2: 14, f. 8–9, 14

***saola* Monastyrskii, 2004**

Specimens: Fig. 14P; Distribution: Fig. 60

**ssp. saola Monastyrskii, 2004**

*Elymnias saola* Monastyrskii, 2004. **TL**: Vietnam: Nghe An Province, Pu Mat Nature Reserve. **TS**: NHM. *Atalanta* 35 (1/2): 45, pl. 2a, figs 1–2; fig. 1A, 3

### *melias*-group

***melias* (C. & R. Felder, 1863)**^48^

Specimens: Fig. 15A–D; Male Genitalia: Fig. 25C; Distribution: Fig. 61

**ssp. melias (C. & R. Felder, 1863)**

*Melanitis melias* C. & R. Felder, 1863. **TL**: Lugban (Philippines: Luzon, Quezon, Lucban) and Burias Island (Philippines: Masbate, Burias Island). **TS**: NHMW. *Wien. ent. Monats.* 7 (4): 120.

**ssp. malis Semper, 1887**

*Elymnias melias malis* Semper, 1887**. TL**: Casiguran (Philippines: Central Luzon, Aurora, Casiguran). **TS**: SMFD. *Reisen Philipp.* (2): 62, pl. 12, figs 2–3.

*Elymnias palmifolia* Schultze, 1908. **TL**: Cagayang (Philippines: Northern Luzon, Cagayan). **TS**: ECMP. *Philipp. J. Sci* 3 (1): 27, pl. 1, fig. 1.

***beza* (Hewitson, 1877)**

Specimens: Fig. 15E–F; Male Genitalia: Fig. 25D; Distribution: Fig. 62.

**ssp. beza (Hewitson, 1877)**

*Melanitis beza* Hewitson, 1877. **TL**: Philippines: Mindanao. **TS**: NHM. *Ent. Mon. Mag.* 13: 179.

*Elymnias kochi plateni* Fruhstorfer, 1907. **syn. n. TL**: Philippines: Mindanao. **TS**: NHM. *Dt. ent. Z. Iris* 20 (3): 228.^49^

**ssp. samarana Schröder & Treadaway, 1980**

*Elymnias beza samarana* Schröder & Treadaway, 1980. **TL**: Philippines: Samar, San Rafael. **TS**: SMFD. *Ent. Z.* 90 (21): 236, fig. 2.

***sansoni* Jumalon, 1975**

Specimens: Fig. 15G–J; Male Genitalia: Fig. 25E; Distribution: Fig. 63

**ssp. sansoni Jumalon, 1975**

*Elymnias sansoni* Jumalon, 1975. **TL**: Philippines: Negros. **TS**: JPC. *Trans. Lep. Soc. Jpn.* 26 (2): 47.

**ssp. aklanensis Uémura & Kitamura, 2001**

*Elymnias sansoni aklanensis* Uémura & Kitamura, 2001. **TL**: Philippines: Panay, Aklan Province, Makato, Castillo. **TS**: TME. *Butterflies* 29: 5.

***luteofasciata* Okubo, 1980**

Specimens: Fig. 15K–L; Distribution: Fig. 64

*Elymnias luteofasciata* Okubo, 1980. **TL**: Philippines: Mindanao, Davao, Penangudloton, Upian River, Calinan. TS: OPC. *Tyô to Ga* 31 (1,2): 60.

### *vitellia*-group

***vitellia* (Stoll, [1781])**

Specimens: Fig. 15M–P; Male Genitalia: Fig. 25F–G; Distribution: Fig. 65

**ssp. vitellia (Stoll, [1781])**

*Papilio vitellia* Stoll, [1781]. **TL**: Ambon (Indonesia: Maluku, Ambon). **TS**: unknown. *Uitl. Kapellen.* 4 (30): 116, pl. 349, fig. E–F.

*Melanitis stellaris* Snellen van Vollenhoven, 1861. **TL**: [New Guinea]. **TS**: NBC. *Tijdschr. Ent.* 4 (5/6): 159, pl. 8, fig. 3.

Elymnias vitellia f. basium Fruhstorfer, 1907. **unavailable name. TL**: Saparua (Indonesia: Maluku, Saparau). **TS**: NHM. *Dt. ent. Z. Iris* 20 (3): 230.

*Elymnias vitellia ceramensis* Martin, 1909. **TL**: Ceram (Indonesia: Maluku, Seram). **TS**: NHMT. *Dt. ent. Z. Iris* 22 (1): 65.

**ssp. viminalis Wallace, 1869**

*Elymnias viminalis* Wallace, 1869. **TL**: Buru Island (Indonesia: Maluku, Buru). **TS**: NHM. *Trans. ent. Soc. Lond.* 1869 (4): 328.

***agondas* (Boisduval, 1832)**^50^

Specimens: Figs 16A–H, 17A–I, 18A–M, 19A–M; Male Genitalia: Fig. 25H–N; Distribution: Fig. 66

**ssp. agondas (Boisduval, 1832)**^51^

*Dyctis agondas* Boisduval, 1832. **TL**: Vanikoro (Solomon Islands: Temotu Province, Vanikoro). **TS**: unknown. *Voy. Astrolabe.* 1: 138.

*Dyctis bioculatus* Westwood, 1851. **syn. n. TL**: Arfak Mountains (Indonesia: West Papua). **TS**: NHM. *Gen. diurn. Lep.* 2: 354, pl. 54, fig. 4.

*Elymnias agondas muscosa* Fruhstorfer, 1907. **TL**: Kapaur (Indonesia: West Papua, Fakfak). **TS**: NHM. *Dt. ent. Z. Iris* 20 (3): 243.

*Elymnias agondas tampyra* Fruhstorfer, 1907. **TL**: Kumusi River (Papua New Guinea: Northern Province, Kumusi River). **TS**: NHM. *Ent. Rundschau* 31 (5): 25.

*Elymnias agondas hagias* Fruhstorfer, 1914. **TL**: Eilandenfluß (Indonesia: Papua, Pulau River). **TS**: NHM. *Ent. Rundschau* 31 (5): 25

**ssp. melane (Hewitson, 1858)**

*Melanitis melane* Hewitson, 1858. **TL**: [Key Island] (Indonesia: Maluku, Kei Island). **TS**: NHM. *Proc. zool. Soc. Lond.* 1858: 465, pl. 55, figs 2, 4.

Elymnias (Dyctis) mela de Nicéville, 1902. **TL**: Key Island (Indonesia: Maluku, Kei Island). **TS**: IM. *J. Bomb. nat. Hist. Soc.* 14 (2): 238, pl. FF, figs 4–5.

Elymnias (Dyctis) meletus de Nicéville, 1902. **TL**: Key Island (Indonesia: Maluku, Kei Island). **TS**: IM. *J. Bomb. nat. Hist. Soc.* 14 (2): 241.

Elymnias (Dyctis) melitia de Nicéville, 1902. **TL**: Key Island (Indonesia: Maluku, Kei Island). **TS**: IM. *J. Bomb. nat. Hist. Soc.* 14 (2): 242.

**ssp. melantho Wallace, 1869**

*Elymnias melantho* Wallace, 1869. **TL**: Gagie Island (Indonesia: West Papua, Raja Ampat Regency, Gag Island). **TS**: NHM. *Trans. ent. Soc. Lond.* 1869 (4): 330.

*Elymnias agondas moranda* Fruhstorfer, 1904. **TL**: Waigeu (Indonesia: West Papua, Raja Ampat Regency, Waigeo). **TS**: NHM. *Dt. ent. Z. Iris* 16 (2): 322.

**ssp. glaucopis Staudinger, 1894**

*Elymnias glaucopis* Staudinger, 1894. **TL**: Sattelberg (Papua New Guinea: Morobe Province, Huon Peninsula, Sattelberg). **TS**: ZMHB. *Dt. ent. Z. Iris* 7 (1): 116.

*Elymnias agondas glaucopis* Fruhsforfer, 1907. **TL**: New Guinea. **TS**: NHM. *Dt. ent. Z. Iris* 20 (3): 243.

**ssp. melanippe Grose-Smith, 1894**

*Elymnias melanippe* Grose-Smith, 1894. **TL**: Sattelberg (Papua New Guinea: Morobe Province, Huon Peninsula, Sattelberg). **TS**: NHM. *Novit. Zool.* 1 (3): 587.

*Elymnias vertenteni* Hulstaert, 1925. **TL**: Irian Jaya (Indonesia: Papua or West Papua). **TS**: NBC. *Ann. Mag. nat. Hist.* (9) 15 (88): 447.

**ssp. melanthes Grose-Smith & Kirby, 1897**

*Elymnias melanthes* Grose-Smith & Kirby, 1897. **TL**: Woodlark Island (Papua New Guinea: Milne Bay, Woodlark Island). **TS**: NHM. *Ann. Mag. nat. Hist.* (6) 19: 178.

Elymnias agondas melanthes f. infernalis (♀) Fruhstorfer, 1914. **TL**: Not indicated. **TS**: NHM. *Ent. Rundschau* 31 (5): 26.

Elymnias agondas melanthes f. virginalis (♀) Fruhstorfer, 1914. **TL**: Not indicated. **TS**: NHM. *Ent. Rundschau* 31 (5): 26.

**ssp. melagondas Fruhstorfer, 1900**

*Elymnias melagondas* Fruhstorfer, 1900. **TL**: New Guinea. **TS**: NHM. *Stett. ent. Ztg.* 60 (10-12): 339.

Elymnias agondas melagondas f. taenarides (♀) Fruhstorfer, 1914. **TL**: Milnebai (Papua New Guinea: Milne Bay). **TS**: NHM. *Ent. Rundschau.* 31 (5): 26.

**ssp. australiana Fruhstorfer, 1900**

*Elymnias australiana* Fruhstorfer, 1900. **TL**: Cape York (Australia: Queensland, Cape York). **TS**: NHM. *Stett. ent. Ztg.* 60 (10-12): 339.

**ssp. aruana Fruhstorfer, 1900**

*Elymnias aruana* Fruhstorfer, 1900. **TL**: Aru (Indonesia: Maluku, Indonesia). **TS**: NHM. *Stett. ent. Ztg.* 60 (10-12): 341.

**ssp. goramensis Fruhstorfer, 1900**

*Elymnias goramensis* Fruhstorfer, 1900. **TL**: Goram Island (Indonesia: Maluku, East Seram Regency, Gorong Island). **TS**: NHM. *Stett. ent. Ztg.* 60 (10-12): 341.

**ssp. agondina Fruhstorfer, 1904**

*Elymnias agondina* Fruhstorfer, 1904. **TL**: Salewatti (Indonesia: West Papua, Raja Ampat Islands, Salawati). **TS**: NHM. *Dt. ent. Z. Iris* 16 (2): 322.

**ssp. dampierensis Rothschild, 1915**

*Elymnias dampierensis* Rothschild, 1915. **TL**: Dampier (Papua New Guinea: Madang, Karkar Island). **TS**: NHMT. *Novit. Zool.* 22 (2): 201.

**ssp. multocellata van Eecke, 1915**

*Elymnias multocellata* van Eecke, 1915. **TL**: Kloofbivak (Indonesia: Papua). **TS**: NBC. *Nova Guinea* 13 (1): 66, pl. 3, f. 6.

**ssp. thryallis Kirsch, 1876. comb. n.**^48^ ^48^

*Elymnias thryallis* Kirsch, 1876. **TL**: Mysore, Kordo (Indonesia: Papua, Biak). **TS**: SMTD. *Mitt. zool. Mus. Dresden* 1: 119, pl. 6, fig. 4.

*Elymnias glauconia* Staudinger, 1894. **TL**: Kubary (Papua New Guinea: Jiwaka, Mt. Kubari). **TS**: ZMHB. *Dt. ent. Z. Iris* 6 (2): 362, pl. 6, fig. 2.

Elymnias glauconia var. chloera Staudinger, 1894. **TL**: New Guinea. **TS**: ZMHB. *Dt. ent. Z. Iris* 6 (2): 363.

Elymnias thryallis f. brunnescens Fruhstorfer, 1911. **TL**: New Guinea. **TS**: NHM. *Gross-Schmett. Erde* 9: 389.

Elymnias thryallis f. pseudosalpinx Fruhstorfer, 1911. **TL**: New Guinea. **TS**: NHM. *Gross-Schmett. Erde* 9: 389.

Elymnias thryallis f. terentilina Fruhstorfer, 1911. **TL**: New Guinea. **TS**: NHM. *Gross-Schmett. Erde* 9: 389.

Elymnias thryallis f. violacea Fruhstorfer, 1911. **TL**: Waigiu Island (Indonesia: West Papua, Raja Ampat Regency, Waigeo). **TS**: NHM. *Gross-Schmett. Erde* 9: 389.

***cybele* (C. & R. Felder, 1860)**

Specimens: Fig. 20A–F; Male Genitalia: Figs 25O, 26A; Distribution: Fig. 67

**ssp. cybele (C. & R. Felder, 1860)**^52^

*Melanitis cybele* C. & R. Felder, 1860. **TL**: Batschian Island (Indonesia: North Maluku, Bacan). **TS**: NHMW. *Wien. ent. Monats.* 4 (8): 248.

*Dyctis astrifera* Butler, 1874. **TL**: Batchian (Indonesia: North Maluku, Bacan). **TS**: NHM. *Trans. ent. Soc. Lond.* 1874 (4): 425.

*Elymnias cybele opaca* Fruhstorfer, 1907. **syn. n. TL**: Halmaheira (Indonesia: North Maluku, Halmahera). **TS**: NHM. *Dt. ent. Z. Iris* 20 (3): 229.

*Elymnias cybele ternatana* **syn. n.** Fruhstorfer, 1907. **TL**: Ternate (Indonesia: North Maluku, Ternate). **TS**: NHM. *Dt. ent. Z. Iris* 20 (3): 229.

**ssp. obiana Fruhstorfer, 1904**

*Elymnias obiana* Fruhstorfer, 1904. **TL**: Obi Island (Indonesia: North Maluku, Obi). **TS**: NHM. *Dt. ent. Z. Iris* 16 (2): 321.

**ssp. adumbrata Fruhstorfer, 1907. subsp. rev.**^53^

*Elymnias cybele adumbrata* Fruhstorfer, 1907. **TL**: Buru (Indonesia: Maluku, Buru). **TS**: NHM. *Dt. ent. Z. Iris* 20 (3): 228.

***cumaea* (C. & R. Felder, [1867])**^54^

Specimens: Fig. 20G–H; Male Genitalia: Fig. 26B; Distribution: Fig. 68

**ssp. cumaea (C. & R. Felder, [1867])**^55^

*Melanitis cumaea* C. & R. Felder, [1867]. **TL**: Halmahera (Indonesia: North Maluku Halmahera). **TS**: NHMW. *Reise. Fregatte. Novara.* 2 (3): 452, pl. 452., pl. 61, f. 9–10.

**ssp. thyone Fruhstorfer, 1904. comb. n., stat. n.**^56^

*Elymnias thyone* Fruhstorfer, 1904. **TL**: [North Celebes] (Indonesia: North Sulawesi, Indonesia). **TS**: NHM. *Soc. Ent.* 19: 53.

**ssp. toliana Fruhstorfer, 1899**^57^

*Elymnias cumaea toliana* Fruhstorfer, 1899. **TL**: Toli Toli (Indonesia: Central Sulawesi, Tolitoli). **TS**: NHM. *Berl. Ent. Zs.* 44 (1/2): 53.

*Elymnias pseudeuploea* Fruhstorfer, 1911. **unavailable name. TL**: Sulawesi (Indonesia: Sulawesi). **TS**: NHM. *Gross-Schmett. Erde* 9: 385.

***hewitsoni* Wallace, 1869**

Specimens: Fig. 20I–J; Male Genitalia: Fig. 26C; Distribution: Fig. 69

**ssp. hewitsoni Wallace, 1869**

*Elymnias hewitsoni* Wallace, 1869. **TL**: Macassar (Indonesia: South Sulawesi, Makassar). **TS**: NHM. *Trans. ent. Soc. Lond.* 1869 (4): 327.

Elymnias hewitsoni hewitsoni f. sumptuosa Fruhstorfer, 1907. **TL**: Tanetta (Indonesia: Central Sulawesi, Poso Regency, Tentena). **TS**: NHM. *Dt. ent. Z. Iris* 20 (3): 237.

**ssp. meliophila Fruhstorfer, 1896**

*Elymnias meliophila* Fruhstorfer, 1896. **TL**: Saleyer (Indonesia: South Sulawesi, Selayar Islands, Selayar). **TS**: NHM. *Soc. Ent.* 11 (4): 25.

**ssp. atys Fruhstorfer, 1904**

*Elymnias hewitsoni atys* Fruhstorfer, 1904. **TL**: Bouthain, south Celebes (Indonesia: South Sulawesi, Moncong Lompobatang). **TS**: NHM. *Soc. Ent.* 19 (8): 60.

***mimalon* (Hewitson, 1861)**

Specimens: Fig. 20K–N; Male Genitalia: Fig. 26D; Distribution: Fig. 70

**ssp. mimalon (Hewitson, 1861**)

*Melanitis mimalon* (Hewitson, 1861). **TL**: Toli-Toli (Indonesia: Central Sulawesi, Tolitoli). **TS**: NHM. *Proc. zool. Soc. Lond.* 1861: 52, pl. 9, figs 1–2.

Elymnias mimalon mimalon f. leucostigmata Fruhstorfer, 1907. **TL**: Toli-Toli (Indonesia: Central Sulawesi, Tolitoli). **TS**: NHM. *Dt. ent. Z. Iris* 20 (3): 239.

**ssp. ino Fruhstorfer, 1904**

*Elymnias mimalon ino* Fruhstorfer, 1904. **TL**: Tawaya, Celebes (Indonesia: Central Sulawesi, Towaya). **TS**: NHM. *Soc. Ent.* 19 (7): 53.

**ssp. nysa Fruhstorfer, 1907**

*Elymnias mimalon nysa* Fruhstorfer, 1907. **TL**: South Celebes (Indonesia: Southeast Sulawesi). **TS**: NHM. *Dt. ent. Z. Iris* 20 (3): 239, pl. 7, fig. 5.

***hicetas* Wallace, 1869**

Specimens: Fig. 20O–P; Male Genitalia: Figs 26E–F; Distribution: Fig. 71

**ssp. hicetas Wallace, 1869**

*Elymnias hicetas* Wallace, 1869. **TL**: Macassar, south Celebes (Indonesia: South Sulawesi, Makassar). **TS**: NHM. *Trans. ent. Soc. Lond.* 1869 (4): 327.

*Elymnias hicetas bonthainensis* Fruhstorfer, 1899. **syn. n. TL**: Bua Kraeng (Indonesia: South Sulawesi, Mt. Bawakaraeng). **TS**: NHM. *Berl. ent. Zs.* 44 (1/2): 55.^58^

**ssp. hicetina Fruhstorfer, 1904**

*Elymnias hicetas hicetina* Fruhstorfer, 1904. **TL**: Tombugu (Indonesia: Central Sulawesi, Tombuko). **TS**: NHM. *Soc. Ent.* 19 (7): 53.

**ssp. butona Fruhstorfer, 1904**

*Elymnais hicetas butona* Fruhstorfer, 1904. **TL**: North Buton (Indonesia: Southeast Sulawesi, Buton). **TS**: NHM. *Soc. Ent.* 19 (7): 53.

**ssp. rarior Martin, 1929**^59^

*Elymnias hicetas rarior* Martin, 1929. **TL**: Celebes (Indonesia: Sulawesi). **TS**: NHMT. *Mitt. münchn. ent. Ges.* 19: 160.

***holofernes* (Butler, 1882)**

Specimens: Fig. 21A–B; Male Genitalia: Fig. 26G; Distribution: Fig. 72

*Dyctis holofernes* Butler, 1882. **TL**: Duke-of-York Island (Papua New Guinea: East New Britain, Duke of York Island). **TS**: NHM. *Ann. Mag. nat. Hist.* 10 (55): 42.

***bornemanni* Ribbe, 1889. stat. n.**^60^

Specimens: Fig. 21C–D; Male Genitalia: Fig. 26H; Distribution: Fig. 73

*Elymnias bornemanni* Ribbe, 1889. **TL**: Bangkai (Indonesia: Central Sulawesi, Banggai). **TS**: SMTD (?). *Dt. ent. Z. Iris* 2 (1): 183, pl. 3, f. 1–2.

***phrikonis* Fruhstorfer, 1899. stat. n.**^61^

Specimens: Fig. 21E–F; Male Genitalia: Fig. 26I; Distribution: Fig. 74

*Elymnias cumaea phrikonis* Fruhstorfer, 1899. **TL**: Sula Besi and Sula-Mangoli (Indonesia: North Maluku, Sula Islands, Sanana and Mangole). **TS**: NHM. *Berl. ent. Zs.* 44 (1/2): 53.

*Elymnias cumaea relicina* Fruhstorfer, 1907. **syn. n. TL**: Sula Besi (Indonesia: North Maluku, Sula Islands, Sanana). **TS**: NHM. *Dt. ent. Z. Iris* 20 (3): 234.

***sangira* Fruhstorfer, 1899**

Specimens: Fig. 21G–H; Male Genitalia: Fig. 26J; Distribution: Fig. 75

*Elymnias cumaea sangira* Fruhstorfer, 1899. **TL**: Sangir, Sulawesi (Indonesia: North Sulawesi, Sangihe Islands, Sangir Besar). **TS**: NHM. *Berl. ent. Zs.* 44 (1/2): 54.

***umbratilis* Joicey & Noakes, 1915. stat. n.**^62^

Specimens: Fig. 21I–J; Male Genitalia: Fig. 26K; Distribution: Fig. 76

*Elymnias cybele umbratilis* Joicey & Noakes, 1915. **TL**: Biak (Indonesia: Papua, Biak). **TS**: NHM. *Trans. ent. Soc. Lond.* 1915 (2): 195.

***resplendens* Martin, 1929. stat. n.**^63^

Specimens: Fig. 21K–L; Male Genitalia: Fig. 26L; Distribution: Fig. 77

*Elymnias cumaea resplendens* Martin, 1929. **TL**: Celebes (Indonesia: Sulawesi). **TS**: NHMT. *Mitt. münchn. ent. Ges.* 19: 162.

### Species not placed in any group^64^

***singhala* Moore, [1875**]

Specimens: Fig. 21M–N; Male Genitalia: Fig. 26M; Distribution: Fig. 78

*Elymnias singhala* Moore, [1875]. **TL**: Colombo, Ceylon (Sri Lanka: Western Province, Colombo). **TS**: NHM. *Proc. zool. Soc. Lond.* 1874 (4): 568.

## Discussion

Wing patterns of *Elymnias* butterflies appear to be highly evolvable, which facilitates Batesian mimetic resemblance to a variety of phenotypically dissimilar model species. Many *Elymnias* are found on islands in the Indo-Australian Archipelago, and the isolation provided by islands seems to provide the opportunity for divergence and local adaptation, facilitating resemblance to different model species in different locales. The remarkable capacity for phenotypic evolution of wing patterns has resulted in sexually dimorphic mimicry, convergence of distantly related taxa on similar wing patterns, and marked phenotypic divergence among conspecific popuations. These phenomena have previously confounded attempts to produce an accurate taxonomic framework because few if any morphological characters are taxonomically or phylogenetically informative. Wing veination, male genitalia, and female genitalia are remarkably uniform among species of *Elymnias*; only slight variation in male genitalia might be useful for discriminating some species. Species delimitation and diagnosis in *Elymnias* has therefore traditionally relied almost entirely on wing patterns. Our molecular phylogeny, which uses genetic markers presumed to be unrelated to wing phenotypes, has detected multiple instances of similar wing patterns in non-sister *Elymnias* lineages that mimic the same, widespread model species. This similarity seems to be the result of convergent evolution, and we have therefore split these taxa into two or more monophyletic lineages (*e.g., E. cumaea* and *E. cybele* have each been split into four and three different species, respectively). On the other hand, some *Elymnias* species—like other mimetic butterfly taxa (Kunte et al. 2014; Merrill et al. 2015; Thompson and Timmermans 2014)—are polymorphic, with single species expressing different mimetic phenotypes in allopatric populations where they mimic different models. We have identified several instances of one nominal species nested within another, and synonymize these taxa under a single species name (*e.g., E. cottonis* into *E. hypermnestra* and *E. cybele thryallis* into *E. agondas*).

Strong dimorphism caused many early workers to describe males and females as separate species, most of which have been synonymized. In this paper we confirmed Araya’s (2016) conclusion of synonymizing *E. detanii*, known only from males, into *E. nepheronides*, known only from females; this rare species is known only from the Indonesian islands of Flores and Sumbawa. Similarly, *E. vasudeva oberthurii* has been sunk into *E. esaca andersonii*, as these apparently represent different sexes of the same species.

Females of several *Elymias* species, including *E. agondas*, *E. hypermnestra*, and *E. esaca*, are morphologically variable across their range. Rather than recognize every wing pattern variant as a different subspecies, we have synonymized many subspecies into geographically cohesive taxa, for example, within the islands of Borneo or New Guinea.

Much of the mismatch between *Elymnias*’ previous taxonomic framework and its evolutionary history is due to rapid evolutionary change. This resulted in morphologically-delimited nominal species that were polyphyletic. In these cases, our molecular phylogenetic results make delimiting species relatively straightforward. However, there are several cases that are not as clear-cut. For example, we elected to retain *E. esaca* and *E. vasudeva* as distinct species despite their paraphyletic relationship because of marked, species-specific morphological differences in these two parapatrically distributed taxa. Population genetic theory predicts incomplete lineage sorting of genetic loci to persist for some time after speciation, resulting in paraphyletic species; the probability of reciprocal monophyly increases with time since divergence (Avise and Ball 1990). Thus, requiring all species to be monophyletic would underestimate true species diversity (Hickerson et al. 2006), particularly in recently diverged species (Knowles and Carstens 2007) such as *esaca* and *vasudeva*. However, we decided to sink *E. kamara* into *E. casiphone* despite their morphological differences because both taxa are wholly sympatric and because morphologically intermediate specimens are known. We included four specimens of *E. c. casiphone* and four of *E. “kamara*” from Java, Bali, and Lombok in our molecular phylogeny, and the topology of all genetic loci individually and together clearly indicated these taxa were conspecific. We suspect that a genetic switch is responsible for the distinct pair of *E. casiphone casiphone* male and female phenotypes (which mimic *Euploea mulciber* males and females) and the different, sexually dimorphic forms of *E. casiphone kamara*, which mimic other *Euploea* species.

Our molecular phylogeny identifies several examples of allopatrically or parapatrically distributed populations that form distinct, monophyletic sister groups: *E. sansoni sansoni* on Negros and *E. sansoni aklanensis* on Panay; *E. patna* from India and *E. patna* from peninsular Malaysia; *E. vitellia vitellia* from Seram and *E. vitellia viminalis* from Buru; and *E. hypermnestra* from Java and the Lesser Sundas and *E. hypermnestra* from everywhere else. These monophyletic sister lineages would likely be considered different species under a strict phylogenetic species concept, and, in most cases, preliminary Bayesian species delimitation analyses with the program Bayesian Phylogenetics and Phylogeography (BPP; Yang and Rannala 2010) suggest the sister lineages are different species. However, we refrain from splitting these species because we regard the geographic sampling of our phylogenetic work as too sparse, consider the degree of phylogenetic distance between the lineages to be too small, or otherwise fail to find convincing evidence that reciprocal monophyly is the result of anything more than geographical isolation. In addition, a recent simulation study suggests that programs such as BPP delimit population structure, not species (Sukumaran and Knowles 2017). Further work may find convincing evidence to split one or more of these pairs into two species.

Although there is one African and several mainland Asian species, most of *Elymnias*’ diversity is found on the islands of the Indo-Australian Archipelago. Islands are considered laboratories for the study of evolution because they promote isolation and divergence while simplifying the task of delimitating populations and other taxa. Evolutionary study of this taxon provides an excellent opportunity to study the role of archipelagoes in diversification, and the evolutionary genetics of evolutionary novelty and speciation.

## List of taxonomic changes

**New synonyms**

*Elymnias papua bivittata* van Eecke, 1915, of *Elymnias papua papua* Wallace, 1869

Elymnias (Mimadelias) esaca taeniola Fruhstorfer, 1907, of *Elymnias esaca borneensis*, Wallace, 1869

*Elymnias* (*Mimadelias) oberthuri* Fruhstorfer, 1902, of *Elymnias esaca andersonii* (Moore, 1886)

*Elymnias thycana* Wallace, 1869, of *Elymnias vasudeva vasudeva* Moore, 1857

*Mimadelias deva* Moore, 1894, of *Elymnias vasudeva vasudeva* Moore, 1857

*Mimadelias burmensis* Moore, 1893, of *Elymnias vasudeva vasudeva* Moore, 1857

*Elymnias vacudera* [sic] *sinensis* Chou, Zhang & Xie, 2000, of *Elymnias vasudeva vasudeva* Moore, 1857

*Melanyias patnoides* Moore, 1893, of *Elymnias patna patna* (Westwood, 1851)

*Elymnias patna stictica* Fruhstorfer, 1902, of *Elymnias patna patna* (Westwood, 1851)

*Elymnias kuenstleri mariae* Toxopeus, 1936, of *Elymnias kuenstleri* Honrath, [1885]

*Elymnias nigrescens tonkiniana* Fruhstorfer, 1902, of *Elymnias hypermnestra hainana* Moore, 1878

Elymnias hypermnestra nigrescens f. depicta Fruhstorfer, 1907, of *Elymnias hypermnestra hainana* Moore, 1878

*Elymnias hypermnestra septentrionalis* Chou & Huang, 1994, of *Elymnias hypermnestra hainana* Moore, 1878

*Elymnias smithi* Moulton, 1915, of *Elymnias harterti brookei* Shelford, 1904

Elymnias panthera var. labuana Staudinger, 1889, of *Elymnias panthera lutescens* Butler, 1867

*Elymnias panthera lacrima* Fruhstorfer, 1904, of *Elymnias panthera lutescens* Butler, 1867

*Elymnias defasciata* Fruhstorfer, 1911, of *Elymnias panthera lutescens* Butler, 1867

*Elymnias panthera alfredi* Fruhstorfer, 1907, of *Elymnias panthera lutescens* Butler, 1867

*Elymnias congruens photinus* Fruhstorfer, 1907, of *Elymnias congruens congruens* Semper, 1887

*Elymnias congruens phaios* Fruhstorfer, 1907, of *Elymnias congruens congruens* Semper, 1887

*Elymnias congruens rafaela* Fruhstorfer, 1907, of *Elymnias congruens congruens* Semper, 1887

*Elymnias nesaea hermia* Fruhstorfer, 1907, of *Elymnias nesaea nesaea* (Linnaeus, 1764)

*Elymnias nesaea cortona* Fruhstorfer, 1911, of *Elymnias nesaea timandra* Wallace, 1869

*Elymnias nesaea coelifrons* Fruhstorfer, 1907, of *Elymnias nesaea hypereides* Fruhstorfer, 1903

*Elymnias kamara* Moore, [1858], of *Elymnias casiphone casiphone* Geyer, [1827]

*Elymnias kamara lombokiana* Fruhstorfer, 1911, of *Elymnias casiphone praetextata* Fruhstorfer, 1896

*Elymnias casiphone djilantik* Martin, 1909, of *Elymnias casiphone exclusa* de Nicéville, 1898

*Elymnias malelas ivena* Fruhstorfer, 1911, of *Elymnias malelas malelas* (Hewitson, 1863)

*Elymnias malelas nilamba* Fruhstorfer, 1911, of *Elymnias malelas malelas* (Hewitson, 1863)

*Elymnias kochi plateni* Fruhstorfer, 1907, of *Elymnias beza beza* (Hewitson, 1877)

*Dyctis bioculatus* Westwood, 1850, of *Elymnias agondas agondas* (Boisduval, 1832)

*Elymnias cybele opaca* Fruhstorfer, 1907, of *Elymnias cybele cybele* (C. & R. Felder, 1860)

*Elymnias cybele ternatana* Fruhstorfer, 1907, of *Elymnias cybele cybele* (C. & R. Felder, 1860)

*Elymnias hicetas bonthainensis* Fruhstorfer, 1899, of *Elymnias hicetas hicetas* Wallace, 1869

*Elymnias cumaea relicina* Fruhstorfer, 1907, of *Elymnias phrikonis* Fruhstorfer, 1899

**New combinations**

*Elymnias hypermnestra cottonis* (Hewitson, 1874) (*Melanitis cottonis*)

*Elymnias hypermnestra beatrice* Fruhstorfer, 1902 (*Elymnias nigrescens beatrice*)

*Elymnias hypermnestra jennifferae* Suzuki, 2006 (*Elymnias cottonis jennifferae*)

*Elymnias panthera lutescens* Butler, 1867 (*Elymnias lutescens*)

*Elymnias casiphone exclusa* de Nicéville, 1898 (Elymnias (Melynias) exclusa)

*Elymnias agondas thryallis* Kirsch, 1876 (*Elymnias thryallis*)

*Elymnias cumaea thyone* Fruhstorfer, 1904 (*Elymnias thyone*)

**Resurrected combination**

*Elymnias casiphone erinyes* de Nicéville, 1895

**Resurrected subspecies**

*Elymnias cybele adumbrata* Fruhstorfer, 1907

**Status changes**

*Elymnias ceryxoides* de Nicéville, 1895 stat. rev.

*Elymnias panthera lutescens* Butler, 1867 stat. n.

*Elymnias cumaea thyone* Fruhstorfer, 1904 stat. n.

*Elymnias bornemanni* Ribbe, 1889 stat. n.

*Elymnias phrikonis* Fruhstorfer, 1899 stat. n.

*Elymnias umbratilis* Joicey & Noakes, 1915 stat. n.

*Elymnias resplendens* Martin, 1929 stat. n.

**Incertae sedis**

*Elymnias merula* Swinhoe, 1915

*Elymnias leucocyma* Godart, 1819

**Species not placed in any group**

*Elymnias singhala* Moore, [1875]

## Plates

**Format of each legend for specimen figures (1–22)**:

valid species or subspecies name_♂♀_dorsal/ventral_specimen repository_current name of collection locality (Country: State/Province, Locality). D = dorsal; V = Ventral; ♂ = male ♀ = female.

**Format of each legend for male genitalia figures (22–26)**:

valid species or subspecies_specimen repository_current locality name.

See pages 4–5 for abbreviations of specimen repositories.

Each distribution map (Figs 27–78) indicates the subspecies distributions for a single species. The species name is indicated in the lower left corner, and subspecies distributions are indicated with different colors. Red dots indicate the species type locality and black dots indicate subspecies type localities. If the type locality is vague, then the dot is positioned in the center of the area specified. Type localities are not indicated on small islands, where a dot would obscure the landmass on the map.

**Figure 13.**
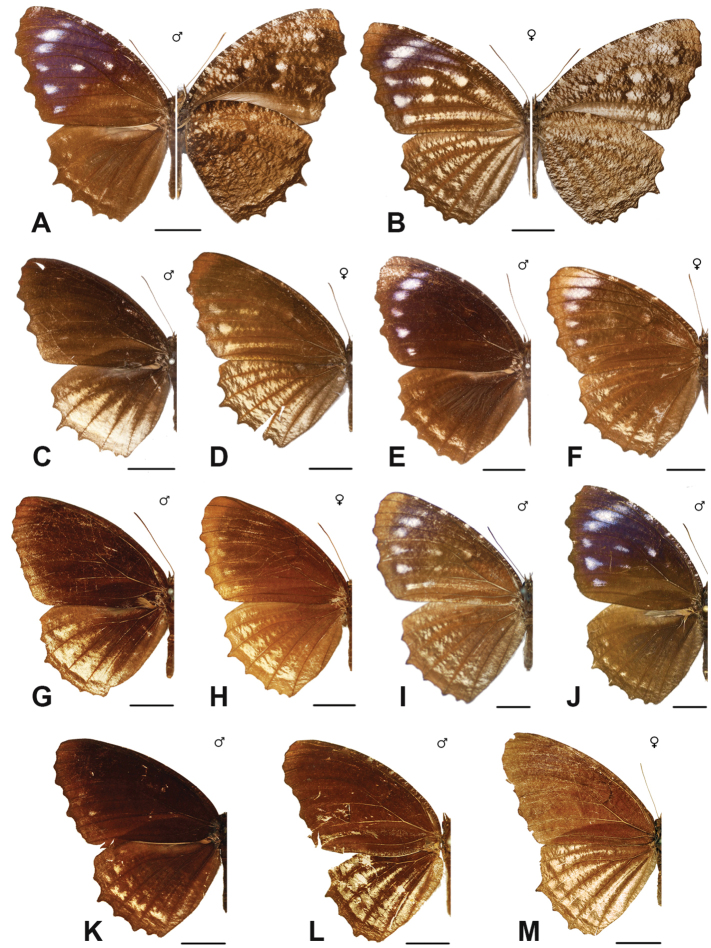
**A** *casiphonecasiphone* ♂ D+V NHM Indonesia: Java **B** *casiphonecasiphone* ♀ D+V NHM Indonesia: Java **C** *casiphonekamara* ♂ D NHM Indonesia: Java **D** *casiphonekamara* ♀ D NHM Indonesia: Java **E** *casiphonepraetextata* ♂ D NHM Indonesia: West Nusa Tenggara, Lombok **F** *casiphonepraetextata* ♀ D NHM Indonesia: West Nusa Tenggara, Lombok **G** *casiphonepraetextata* (=*kamaralombokiana*) ♂ D NHM Indonesia: West Nusa Tenggara, Lombok **H** *casiphonepraetextata* (=*kamaralombokiana*) ♀ D NHM Indonesia: West Nusa Tenggara, Lombok **I** *casiphonepraetextata* (=*kamaralombokiana*) ♀ D NHM Indonesia: West Nusa Tenggara, Lombok; Syntype of *Elymnias kamara lombokiana* **J** *casiphonealumna* ♂ D NHM Indonesia: East Java, Blitar **K** *casiphoneexclusa* ♂ D NHM Indonesia: Bali **L** *casiphoneerinyes* ♂ D NHM Indonesia: Sumatra **M** *casiphoneerinyes* ♀ D NHM Indonesia: Sumatra.

**Figure 14.**
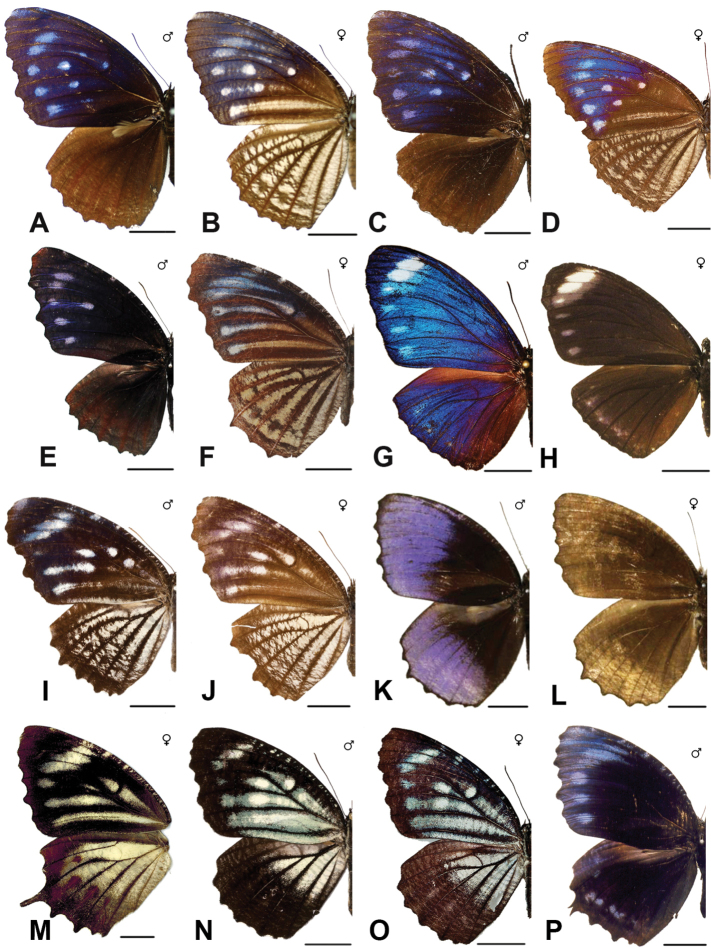
**A** *malelas* ♂ D NHM India: Sikkim **B** *malelas* ♀ D NHM India: Sikkim **C** *malelas* ♂ D MCZ Vietnam **D** *malelas* ♀ D KUTH Thailand: Chiang Mai **E** *saueri* ♂ D IPC Thailand: Phetchabun **F** *saueri* ♀ D NMNH Malaysia: Johor, Mersing to Kluang **G** *kochi* ♂ D SMFD Philippines: Luzon, Sierre Madre Mountain Range **H** *kochi* ♀ D PNM Philippines: Luzon, Sierre Madre Mountain Range **I** *casiphonidescasiphonides* ♂ D NHM Philippines: Mindanao **J** *casiphonidescasiphonides* ♀ D NHM Philippines: Mindanao **K** *nelsoni* ♂ D UPC Indonesia: West Sumatra, Mentawai Regency, Sipora **L** *nelsoni* ♀ D UPC Indonesia: West Sumatra, Mentawai Regency, Sipora **M** *amoena* ♀ D MCZ Indonesia: Sumba, Kombapari Forest **N** *kanekoi* ♂ D NHM Philippines: Negros **O** *kanekoi* ♀ D SMFD Philippines: Negros **P** *saola* ♂ D NHM Vietnam; Holotype.

**Figure 15.**
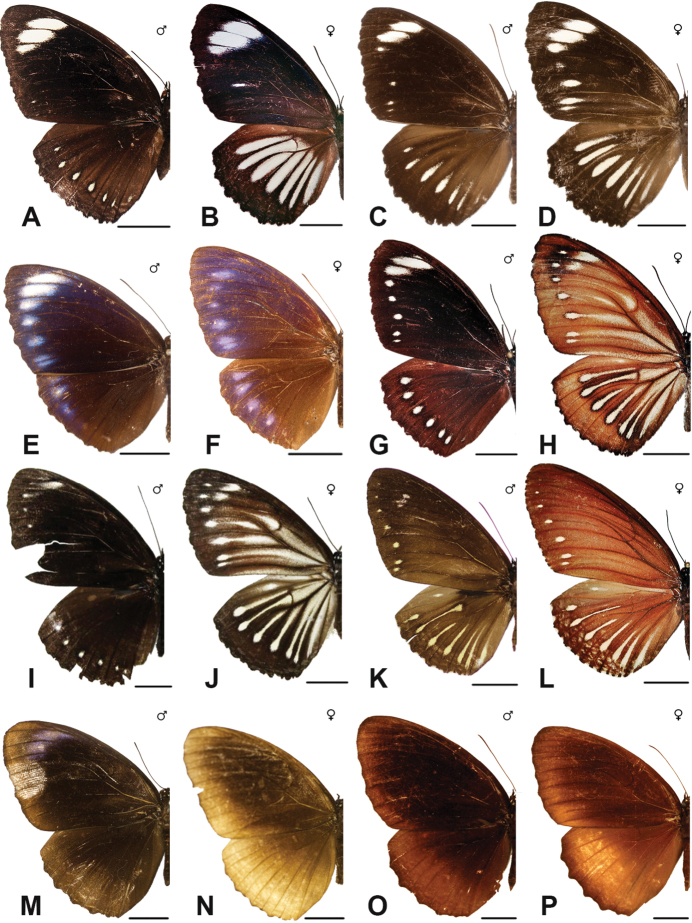
**A** *meliasmelias* ♂ D NMNH Philippines: Luzon, Cavite, Puerto Azul **B** *meliasmelias* ♀ D SMFD Philippines: Luzon, Sierre Madre Mountains **C** *meliasmalis* ♂ D NHM Philippines: Quezon, Polillo Island **D** *meliasmalis* ♀ D NHM Philippines: Luzon, Los Baños **E** *bezabeza* ♂ D NHM Philippines: Mindanao **F** *bezabeza* ♀ D NHM Philippines: Mindanao **G** *sansonisansoni* ♂ D SMFD Philippines: Negros **H** *sansonisansoni* ♀ D SMFD Philippines: Negros **I** *sansoniaklanensis* ♂ D UPC Philippines: Panay, Aklan; Paratype **J** *sansoniaklanensis* ♀ D UPC Philippines: Panay, Aklan; Paratype **K** *luteofasciata* ♂ D OPC Philippines: Mindanao, Penangudltan, Upian River, City of Davao; Holotype **L** *luteofasciata* ♀ D SMFD Philippines: Mindanao, South Cotabato, Mt. Matutum **M** *vitelliavitellia* ♂ D NHM Indonesia: Maluku, Ambon **N** *vitelliavitellia* ♀ D NHM Indonesia: Maluku, Ambon **O** *vitelliaviminalis* ♂ D NHM Indonesia: Maluku, Buru **P** *vitelliaviminalis* ♀ D NHM Indonesia: Maluku, Buru.

**Figure 16.**
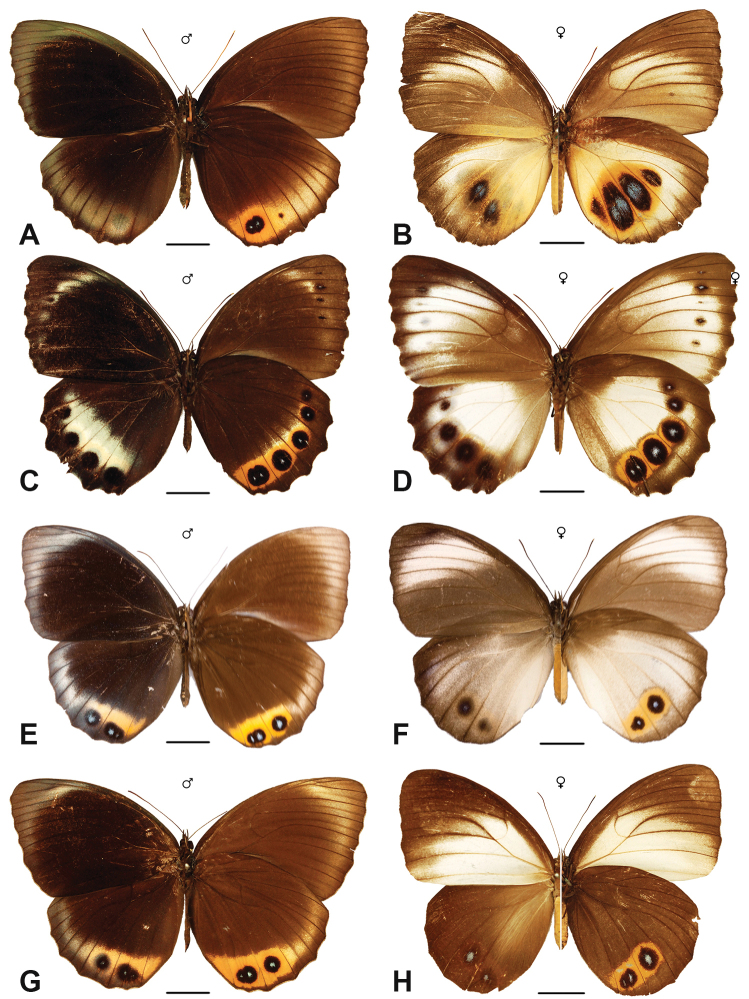
**A** *agondasagondas* ♂ D+V NHM Indonesia: West Papua **B** *agondasagondas* ♀ D+V NHM Indonesia: West Papua **C** *agondasmelane* ♂ D+V NHM Indonesia: Maluku, Kei Island **D** *agondasmelane* ♀ D+V NHM Indonesia: Maluku, Kei Island **E** *agondasglaucopis* ♂ D+V NHM Papua New Guinea: Oro Province, Kumusi River **F** *agondasglaucopis* ♀ D+V NHM Papua New Guinea: Oro Province, Kumusi River **G** *agondasmelanippe* ♂ D+V NHM Papua New Guinea: Morobe Province, Huon Peninsula, Sattelberg **H** *agondasmelanippe* ♀ D+V NHM Papua New Guinea: Morobe Province, Huon Peninsula, Sattelberg.

**Figure 17.**
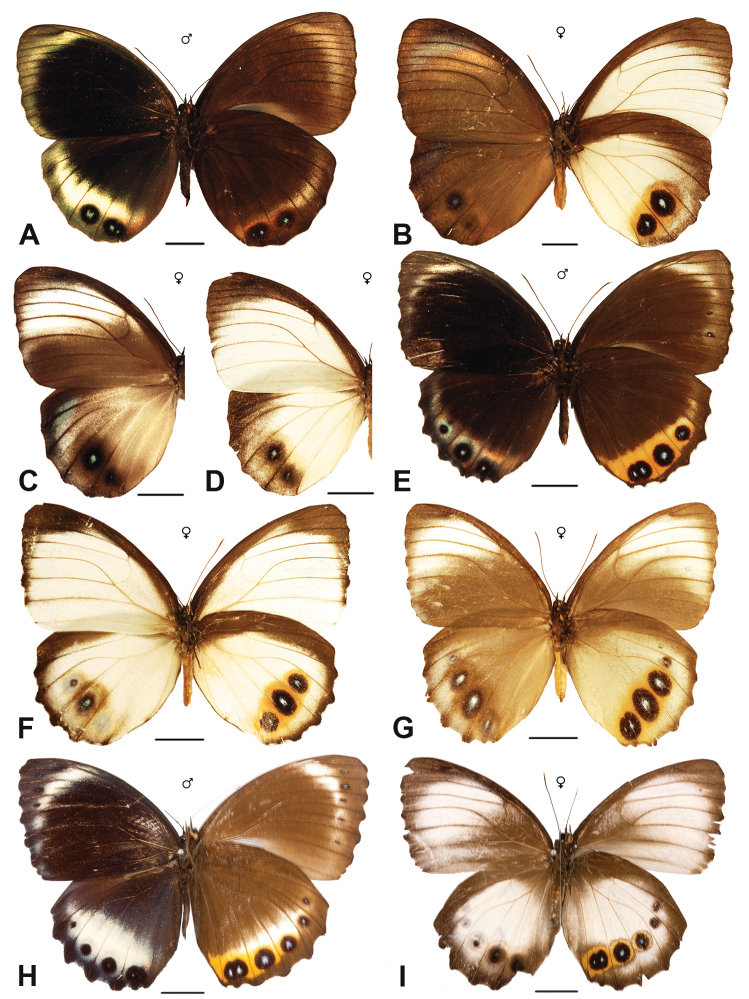
**A** *agondasmelanthes* ♂ D+V NHM Papua New Guinea: Milne Bay, Woodlark Island **B** *agondasmelanthes* ♀ D+V NHM Papua New Guinea: Milne Bay, Woodlark Island **C** *agondasmelanthes* ♀ D NHM Papua New Guinea: Milne Bay, Woodlark Island **D** *agondasmelanthes* ♀ D NHM Papua New Guinea: Milne Bay, Woodlark Island **E** *agondasaruana* ♂ D+V NHM Indonesia: Maluku, Aru **F** *agondasaruana* ♀ D+V NHM Indonesia: Maluku, Aru **G** *agondasaruana* ♀ D+V NHM Indonesia: Maluku, Aru **H** *agondas* ssp. ♂ D+V NHM Indonesia: Maluku, Tanimbar **I** *agondas* ssp. ♀ D+V NHM Indonesia: Maluku, Tanimbar.

**Figure 18.**
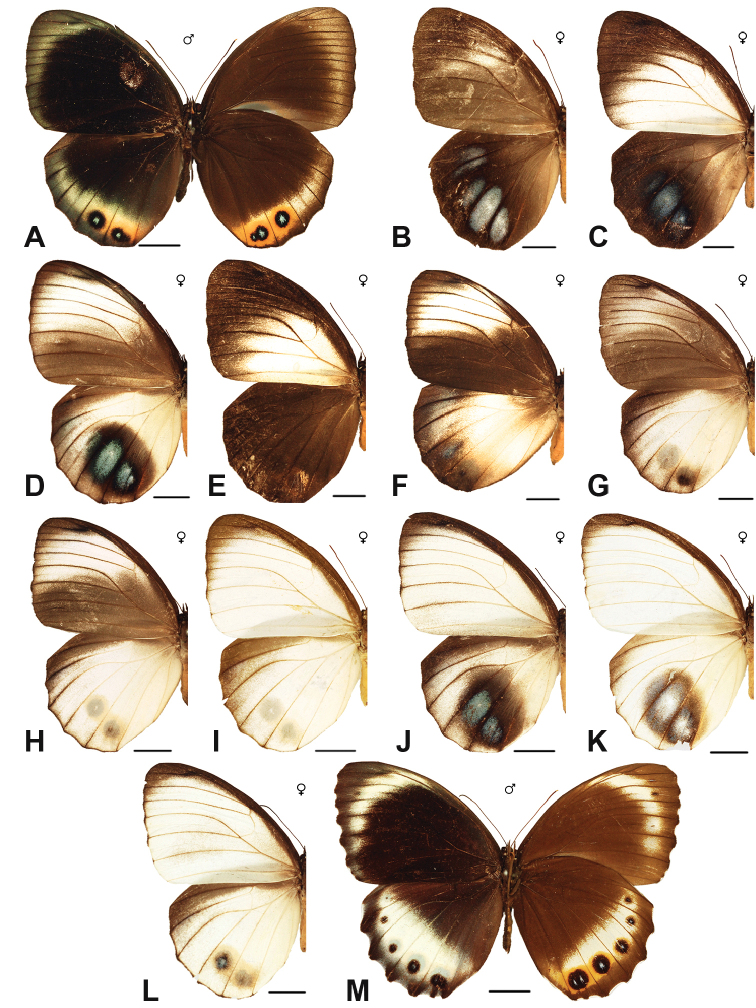
**A** *agondasmelagondas* ♂ D+V NHM New Guinea **B** *agondasmelagondas* ♀ D NHM New Guinea **C** *agondasmelagondas* ♀ D NHM New Guinea **D** *agondasmelagondas* ♀ D NHM New Guinea **E** *agondasmelagondas* ♀ D NHM New Guinea **F** *agondasmelagondas* ♀ D NHM New Guinea **G** *agondasmelagondas* ♀ D NHM New Guinea **H** *agondasmelagondas* ♀ D NHM New Guinea **I** *agondasmelagondas* ♀ D NHM New Guinea **J** *agondasmelagondas* ♀ D NHM New Guinea **K** *agondasmelagondas* ♀ D NHM New Guinea **L** *agondasmelagondas* ♀ D NHM New Guinea **M** *agondasgoramensis* ♂ D+V NHM Indonesia: Maluku, East Seram Regency, Gorong Island.

**Figure 19.**
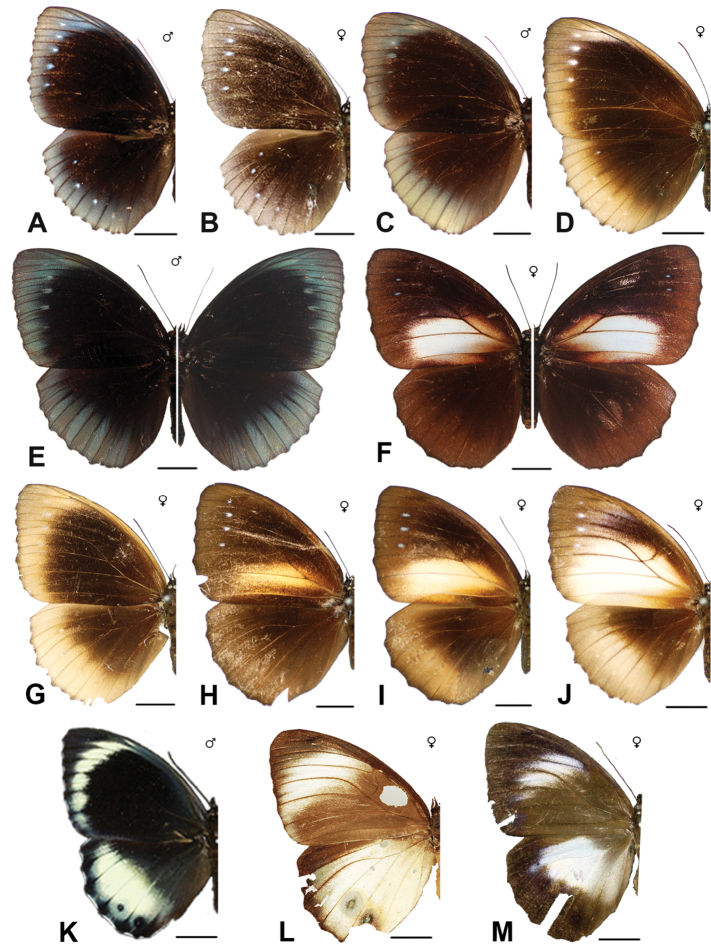
**A** *agondasdampierensis* ♂ D NHM Papua New Guinea: Madang, Karkar Island; Syntype **B** *agondasdampierensis* ♀ D NHM Papua New Guinea: Madang, Karkar Island **C** *agondasthryallis* ♂ D NHM Papua New Guinea **D** *agondasthryallis* ♀ D NHM Papua New Guinea **E** *agondasthryallis* ♂ D+V NMNH Papua New Guinea: East Sepik, Maprik **F** *agondasthryallis* ♀ D+V NMNH Papua New Guinea: Regia, Mapuk **G** *agondasthryallis* ♀ D NHM Indonesia: Papua, Yos Sudarso Bay **H** *agondasthryallis* ♀ D NHM Indonesia: Papua, Yos Sudarso Bay **I** *agondasthryallis* ♀ D NHM Indonesia: Papua, Yos Sudarso Bay **J** *agondasthryallis* ♀ D NHM Indonesia: Papua, Yos Sudarso Bay **K** *agondasaustraliana* ♂ D MCZ Australia: Queensland, Claudie River **L** *agondasaustraliana* ♀ D NHM Australia: Queensland **M** *agondasaustraliana* ♀ D MCZ Australia: Queensland, West Claudie River.

**Figure 20.**
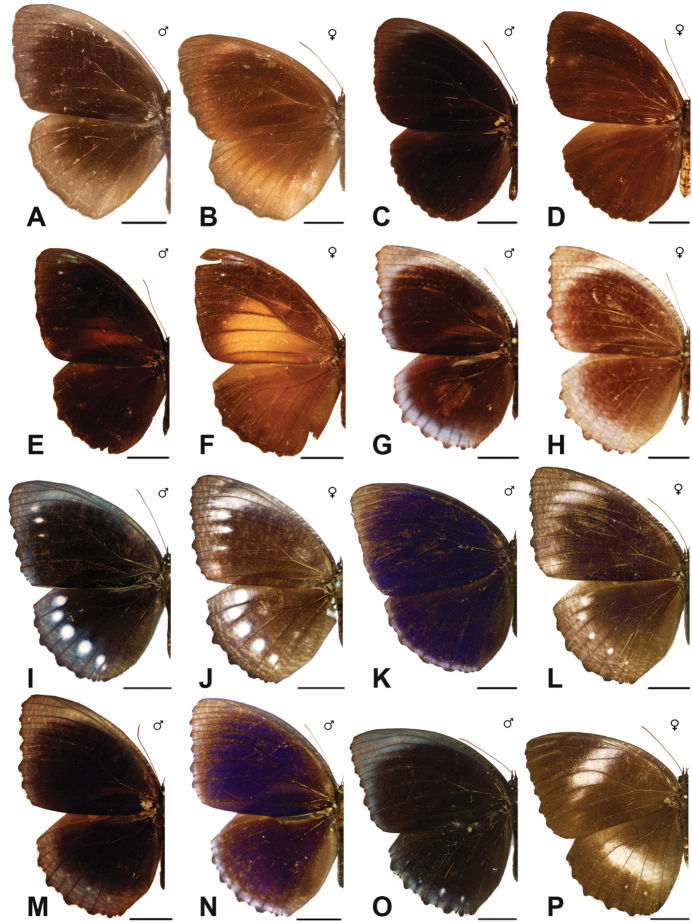
**A** *cybelecybele* ♂ D NHM Indonesia: North Maluku, Halmahera **B** *cybelecybele* ♀ D NHM Indonesia: North Maluku, Halmahera **C** *cybelecybele* ♂ D NHM Indonesia: North Maluku, Bacan **D** *cybelecybele* ♀ D NHM Indonesia: North Maluku, Bacan **E** *cybeleobiana* ♂ D NHM Indonesia: North Maluku, Obi **F** *cybeleobiana* ♀ D NHM Indonesia: North Maluku, Obi **G** *cumaeacumaea* ♂ D NHM Indonesia: North Sulawesi, Menado **H** *cumaeacumaea* ♀ D NHM Indonesia: North Sulawesi, Minahasa **I** *hewitsonihewitsoni* ♂ D NHM Indonesia: South Sulawesi **J** *hewitsonihewitsoni* ♀ D NHM Indonesia: South Sulawesi **K** *mimalonmimalon* ♂ D NHM Indonesia: Sulawesi **L** *mimalonmimalon* ♀ D NHM Indonesia: North Sulawesi, Menado **M** *mimalonnysa* ♂ D NHM Indonesia: South Sulawesi **N** *mimalonino* ♂ D NHM Indonesia: Central Sulawesi; Holotype **O** *hicetashicetas* ♂ D NHM Indonesia: Sulawesi **P** *hicetashicetas* ♀ D NHM Indonesia: South Sulawesi.

**Figure 21.**
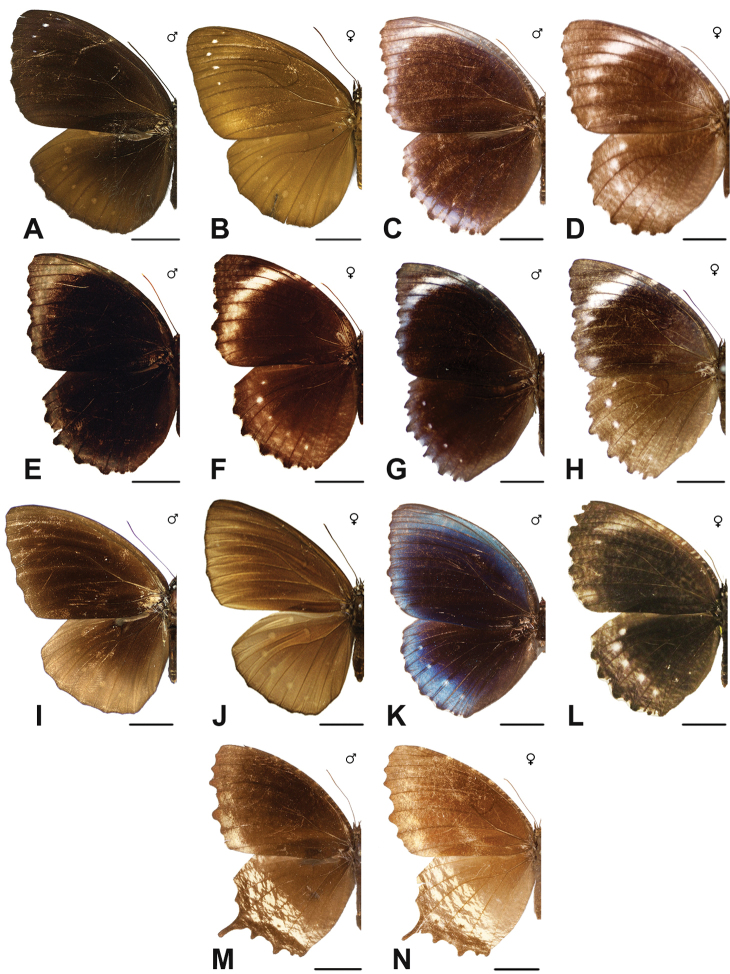
**A** *holofernes* ♂ D NHM Papua New Guinea: New Britain **B** *holofernes* ♀ D NHM Papua New Guinea: New Britain **C** *bornemanni* ♂ D NHM Indonesia: Central Sulawesi, Banggai **D** *bornemanni* ♀ D NHM Indonesia: Central Sulawesi, Banggai **E** *phrikonis* ♂ D NHM Indonesia: Sula Archipelago **F** *phrikonis* ♀ D NHM Indonesia: Sula Archipelago **G** *sangira* ♂ D NHM Indonesia: North Sulawesi, Talaud **H** *sangira* ♀ D NHM Indonesia: North Sulawesi, Talaud **I** *umbratilis* ♂ D NHM Indonesia: Papua, Biak; Holotype **J** *umbratilis* ♀ D OPC Indonesia: Papua, Biak **K** *resplendens* ♂ MCZ Indonesia: Central Sulawesi, Palu **L** *resplendens* ♀ MCZ Indonesia: Central Sulawesi, Palu **M** *singhala* ♂ D NHM Sri Lanka **N** *singhala* ♀ D NHM Sri Lanka.

**Figure 22.**
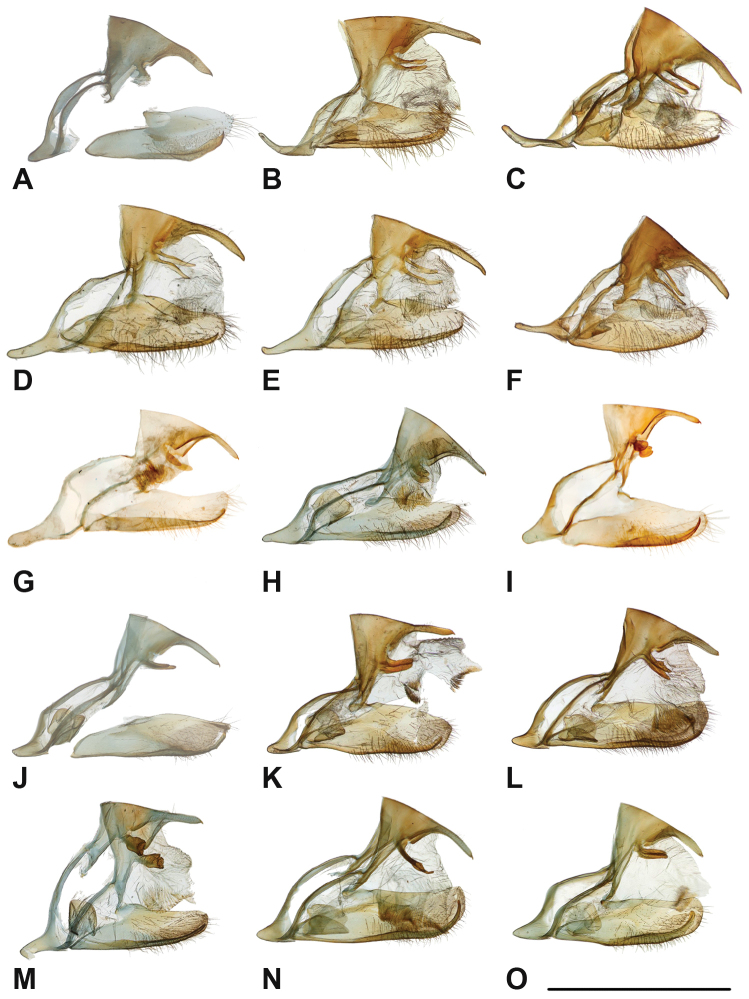
**A** *bammakoobammakoo* NHM Central Africa **B** *paradoxa* NHM Indonesia: Papua, Weyland Mountains **C** *papuacinereomargo* NHM Indonesia: Papua, Biak **D** *esacamaheswara* NHM Indonesia: Java **E** *esacaleontina* NHM Indonesia: North Sumatra, Nias **F** *vasudeva* NHM India: Meghalaya, Khasi Hills **G** *daraalbofasciata* MCZ Philippines: Palawan **H** *darabengena* NHM Indonesia: Java **I** *daradarina* MCZ Peninsular Malaysia: Pahang, Cameron Highlands **J** *patnapatna* NHM India: Sikkim **K** *peali* NHM India: Assam **L** *ceryx* NHM Indonesia: Java **M** *kuenstleri* NHM collection locality unknown **N** *pellucida* NHM East Malaysia: Sabah, Mt. Kinabalu **O** *penangachelensis* NHM Thailand: Ranong.

**Figure 23.**
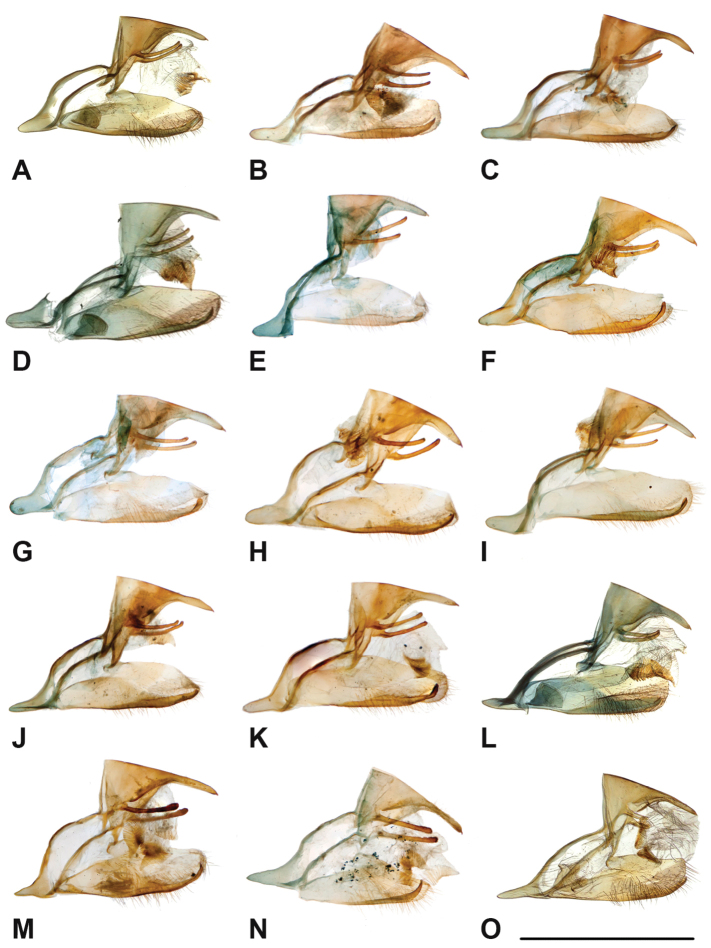
**A** *hypermnestrahypermnestra* NHM Indonesia: West Java, Bogor **B** *hypermnestrahypermnestra* MCZ Indonesia: Maluku, Seram **C** *hypermnestrafraterna* MCZ Sri Lanka: Western Province **D** *hypermnestracottonis* NHM India: Andaman Islands **E** *hypermnestratinctoria* NSYSU Thailand: Trang, Khao Chong **F** *hypermnestrahainana* NSYSU Taiwan: Kaohsiung **G** *hypermnestradiscrepans* NSYSU Peninsular Malaysia: Penang **H** *hypermnestraorientalis* MCZ Indonesia: East Nusa Tenggara, Flores **I** *hypermnestrabaliensis* NSYSU Indonesia: Bali **J** *hypermnestrasumbana* MCZ Indonesia: East Nusa Tenggara, Sumba **K** *hypermnestratimorensis* MCZ Indonesia: East Nusa Tenggara, Timor **L** *caudata* NHM Myanmar (specimen is likely mislabeled) **M** *nepheronidesnepheronides* MCZ Indonesia: East Nusa Tenggara, Flores **N** *parce* MCZ Philippines: Palawan **O** *pantheratautra* NHM Indonesia: Sumatra, Bengkalis, Senggoro.

**Figure 24.**
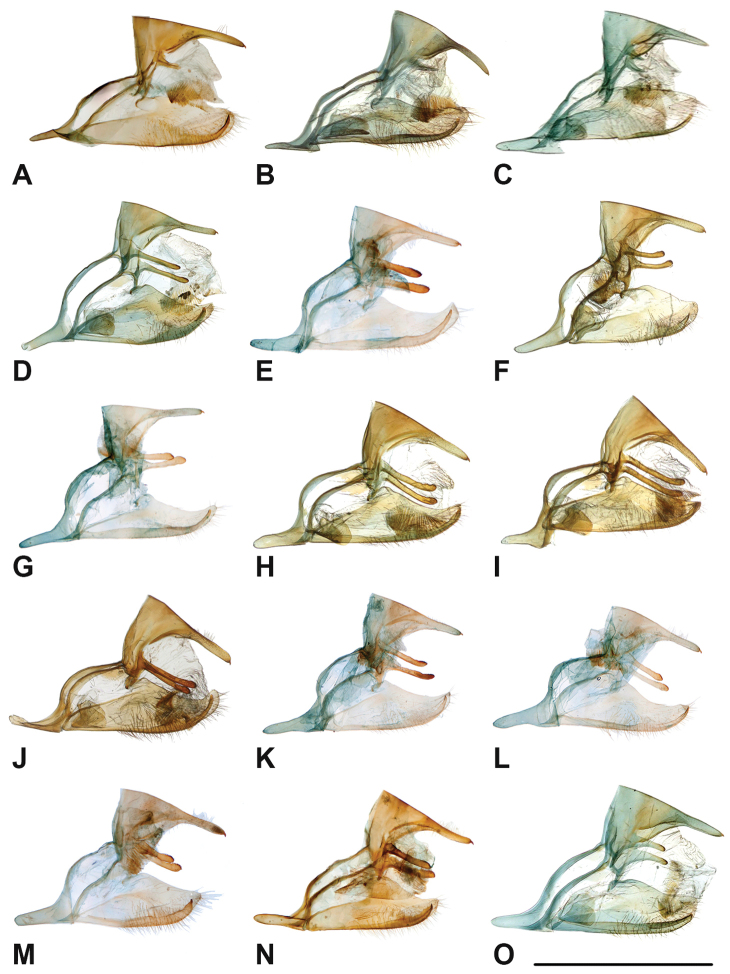
**A** *pantherabalina* MCZ Indonesia: Bali **B** *obnubila* NHM Peninsular Malaysia: Perak **C** *congruenscongruens* NHM Philippines: Cebu, Camotes Island **D** *nesaeanesaea* NHM Indonesia: Java **E** *nesaeanesaea* NSYSU Indonesia: Bali **F** *nesaeatimandra* NHM India: Meghalaya, Khasi Hills **G** *nesaeavordemani* NSYSU Indonesia: East Java, Kangean Islands **H** *casiphonecasiphone* NHM Indonesia: Java **I** *casiphonecasiphone* NHM Indonesia: Java **J** *casiphonepraetextata* NHM Indonesia: East Nusa Tenggara, Lombok **K** *casiphoneexclusa* NSYSU Indonesia: Bali **L** *casiphonealumna* NSYSU Indonesia: Java **M** *malelas* NSYSU Thailand: Chiang Mai **N** *kochi* MCZ Philippines: Luzon, Sierra Madre Range, Isabela **O** *casiphonidescasiphonides* NHM Philippines: Mindanao

**Figure 25.**
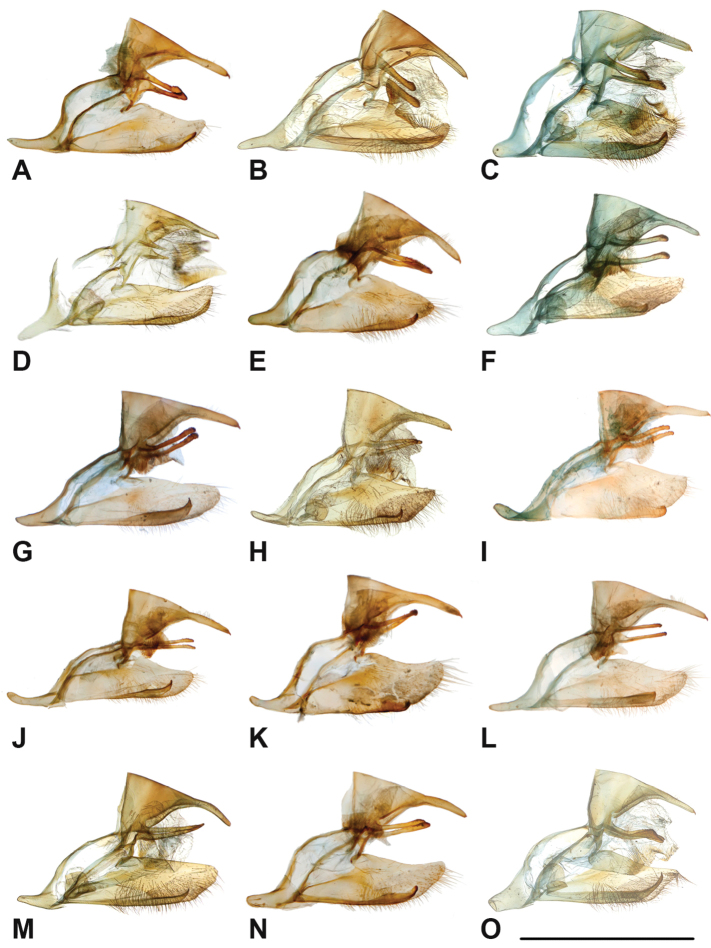
**A** *nelsoni* MCZ Indonesia: West Sumatra, Pagai Island **B** *kanekoi* NHM Philippines: Negros **C** *meliasmalis* NHM Philippines: Quezon, Polillo Island **D** *bezabeza* NHM Philippines: Mindanao **E** *sansoniaklanensis* MCZ Philippines: Panay, Aklan, Mt. Madiaas **F** *vitelliavitellia* NHM Indonesia: Maluku, Ambon **G** *vitelliaviminalis* MCZ Indonesia: Maluku, Buru **H** *agondasglaucopis* NHM Papua New Guinea: Oro Province, Kumusi River **I** *agondasagondas* (previously *E. a. bioculatus*) NSYSU Indonesia: West Papua, Sorong **J** *agondasmelagondas* MCZ Indonesia: West Papua, Sorong **K** *agondasmelagondas* MCZ Indonesia: Papua, Asiki **L** *agondasaruana* MCZ Indonesia: Papua, Aru **M** *agondasthryallis* NHM Papua New Guinea: New Britain **N** *agondasthryallis* MCZ Indonesia: West Papua, Yapen **O** *cybelecybele* NHM Indonesia: North Maluku, Bacan.

**Figure 26.**
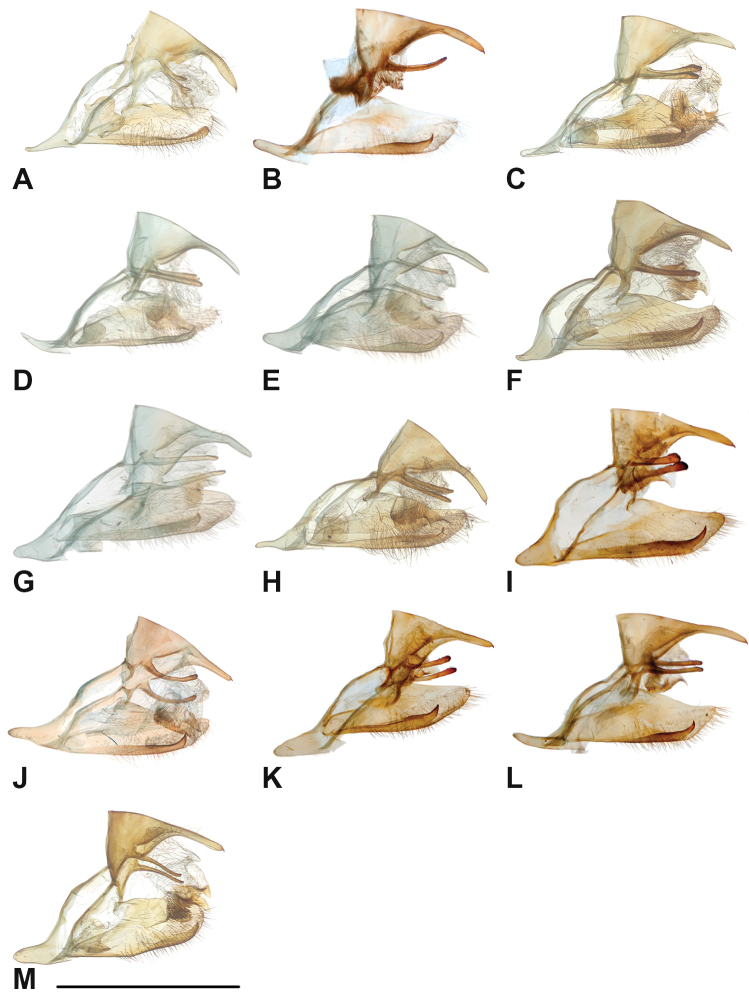
**A** *cybelecybele* NHM Indonesia: North Maluku, Halmahera **B** *cumaeatoliana* MCZ Indonesia: North Sulawesi **C** *hewitsonimeliophila* NHM Indonesia: Maluku, Kisar **D** *mimalonmimalon* NHM Indonesia: Sulawesi **E** *hicetashicetas* NHM Indonesia: Sulawesi **F** *hicetashicetina* NHM Indonesia: Sulawesi **G** *holofernes* NHM Papua New Guinea: New Britain **H** *bornemanni* NHM Indonesia: Central Sulawesi, Banggai **I** *phrikonis* MCZ Indonesia: North Maluku, Sula Regency, Sanana **J** *sangira* NMNH Indonesia: North Sulawesi, Sangir island **K** *umbratilis* MCZ Indonesia: Papua, Biak **L** *resplendens* MCZ Indonesia: Central Sulawesi, Palu **M** *singhala* NHM Sri Lanka

**Figure 27.**
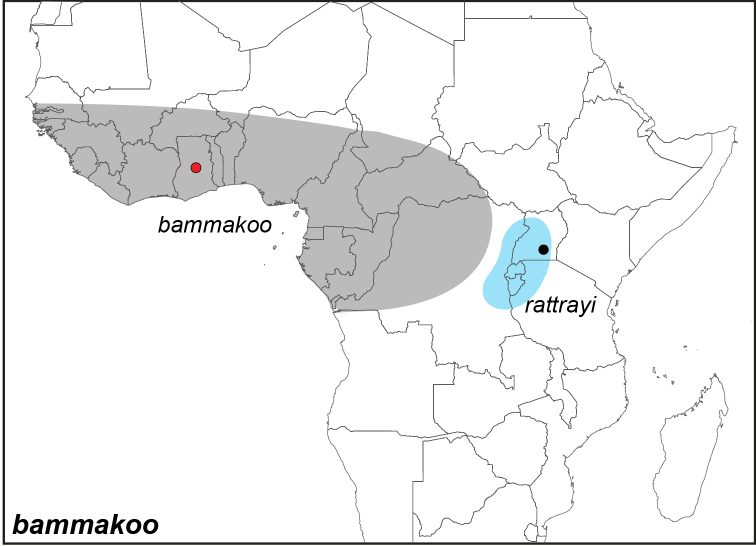
Distribution map of *Elymnias bammakoo*.

**Figure 28.**
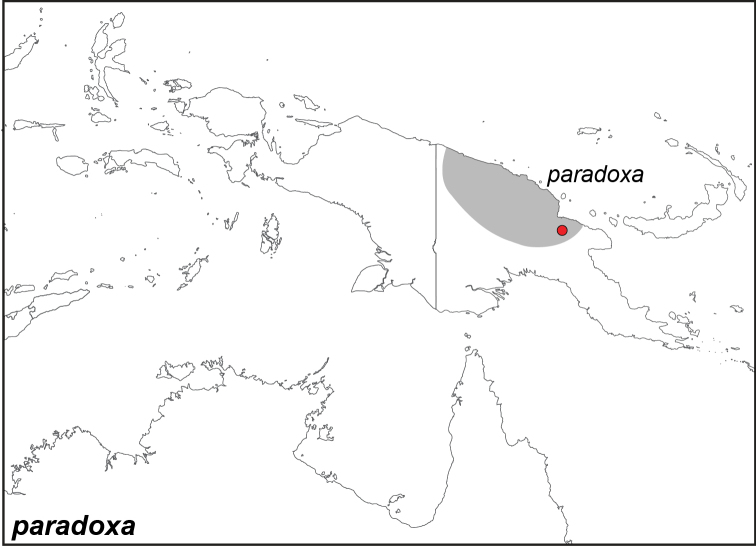
Distribution map of *Elymnias paradoxa*.

**Figure 29.**
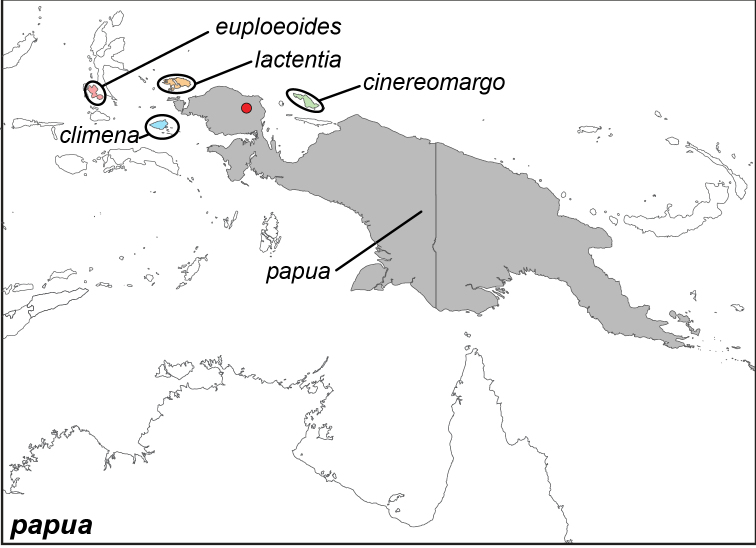
Distribution map of *Elymnias papua*.

**Figure 30.**
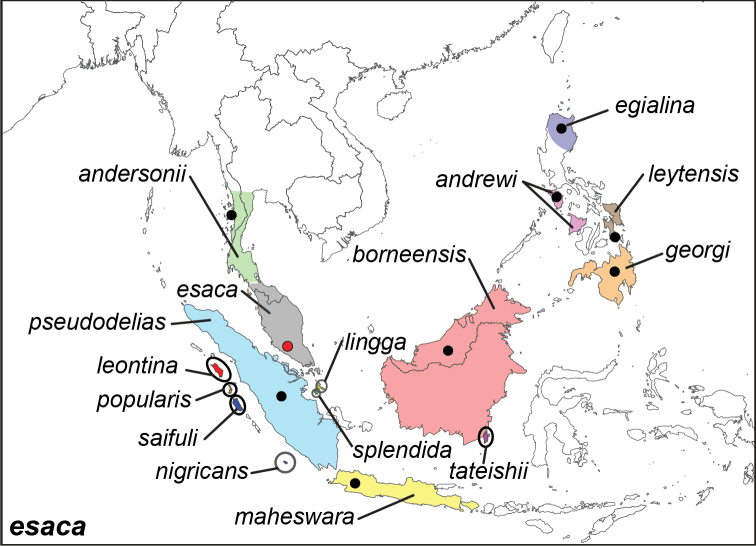
Distribution map of *Elymnias esaca*.

**Figure 31.**
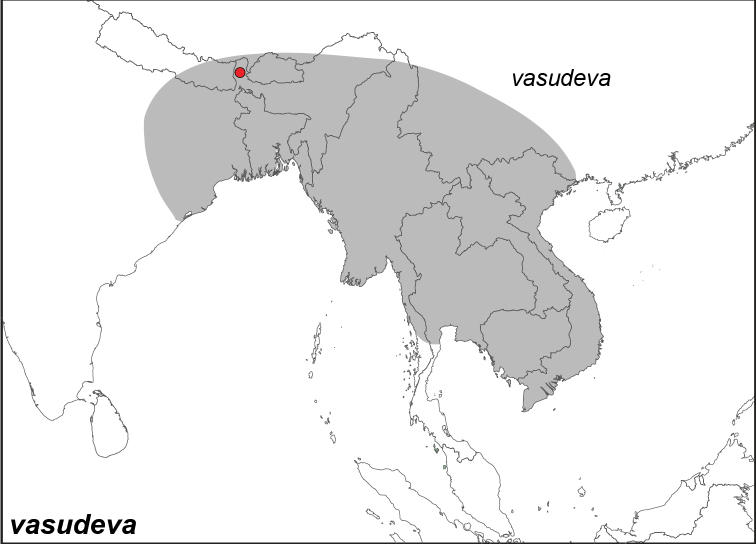
Distribution map of *Elymnias vasudeva*.

**Figure 32.**
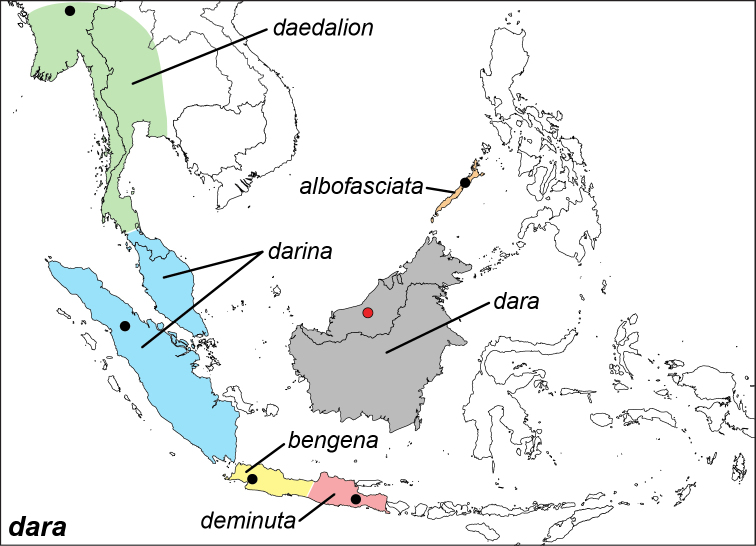
Distribution map of *Elymnias dara*.

**Figure 33.**
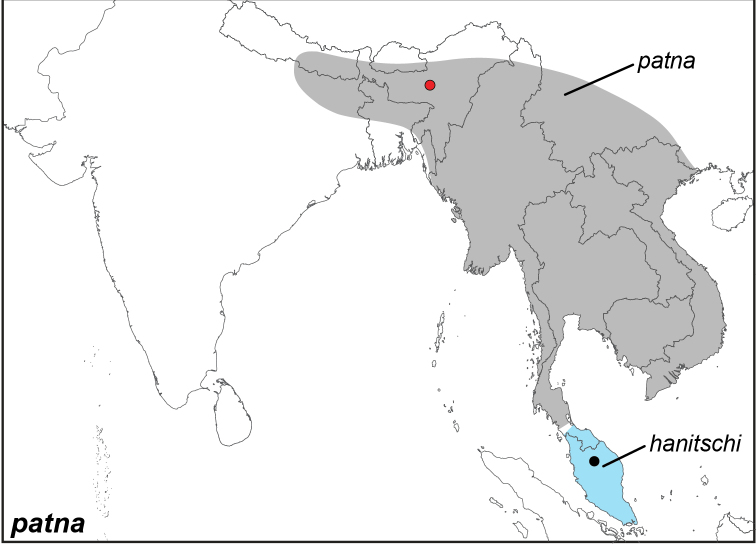
Distribution map of *Elymnias patna*.

**Figure 34.**
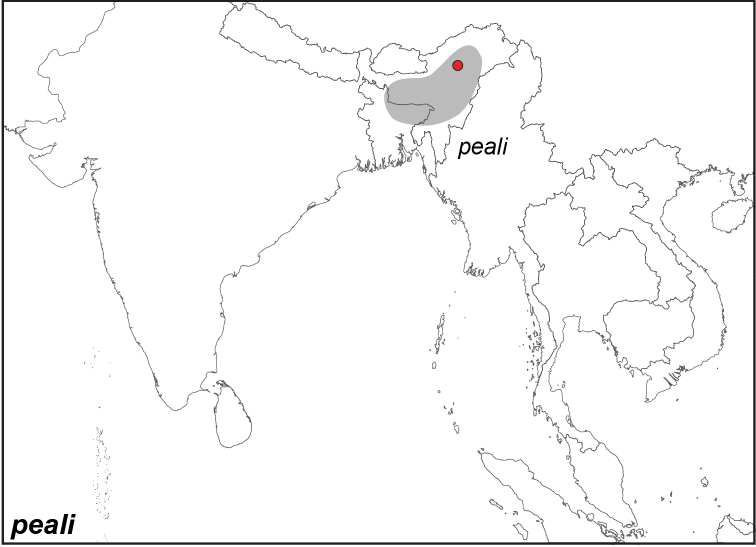
Distribution map of *Elymnias peali*.

**Figure 35.**
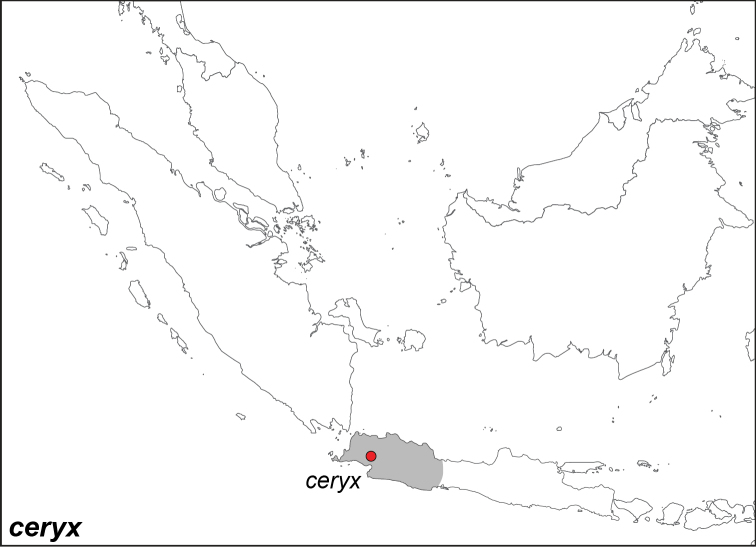
Distribution map of *Elymnias ceryx*.

**Figure 36.**
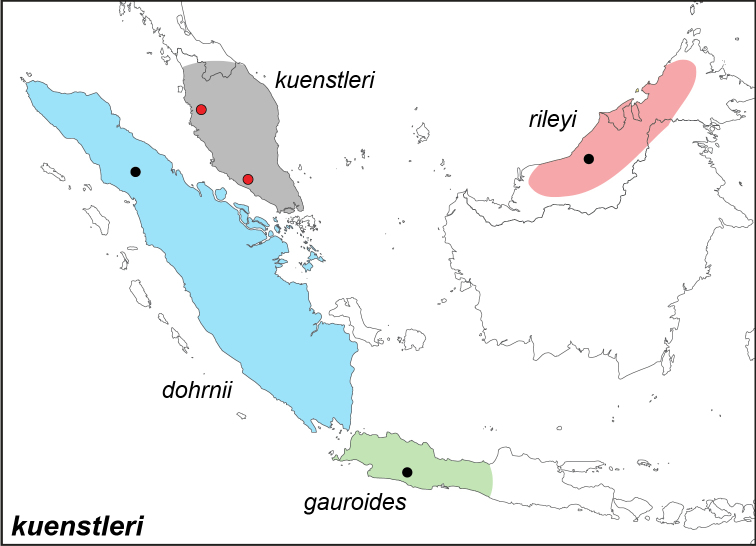
Distribution map of *Elymnias kuenstleri*.

**Figure 37.**
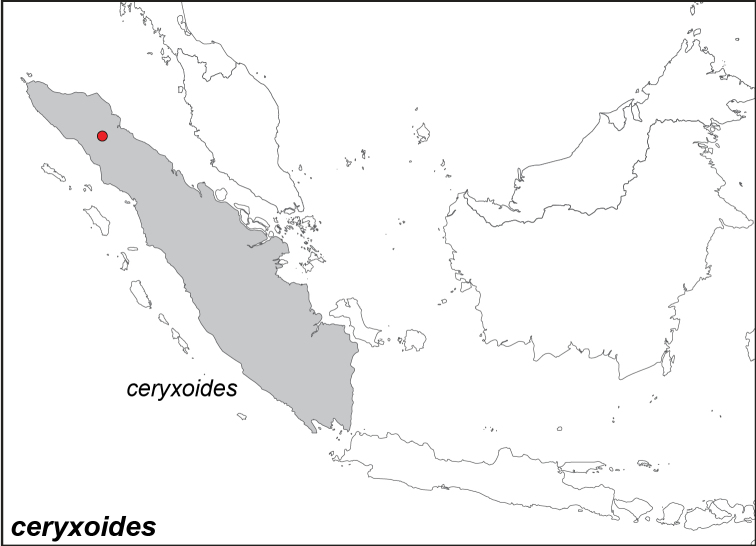
Distribution map of *Elymnias ceryxoides*.

**Figure 38.**
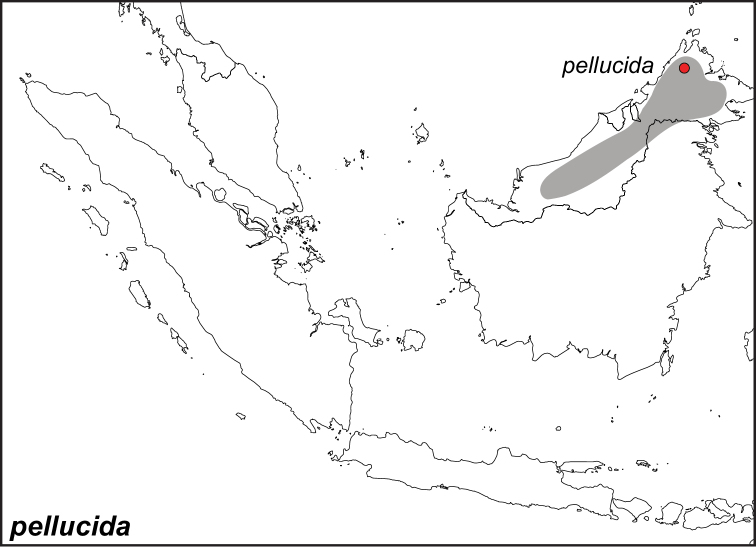
Distribution map of *Elymnias pellucida*.

**Figure 39.**
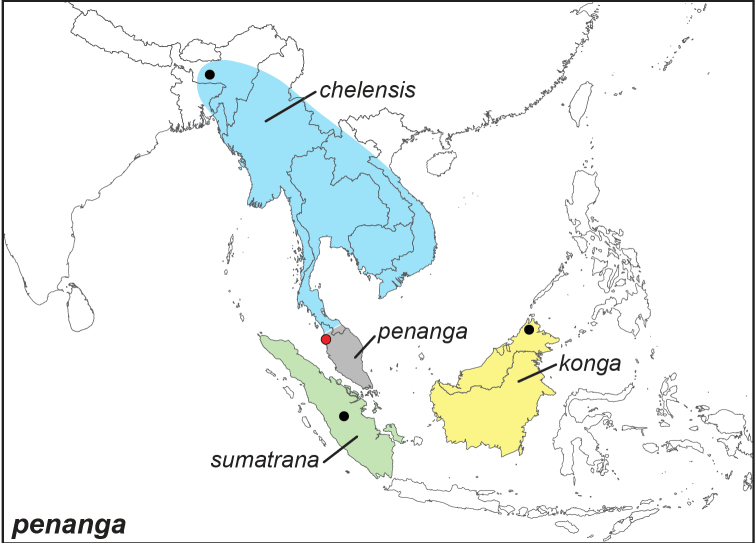
Distribution map of *Elymnias penanga*.

**Figure 40.**
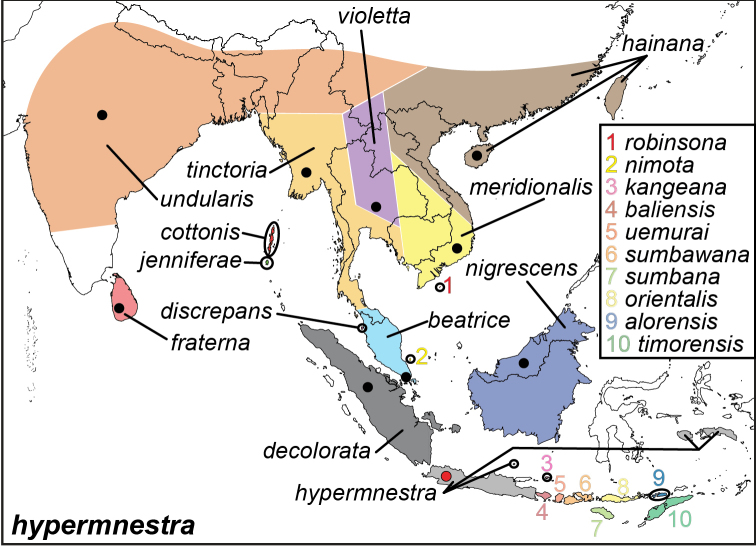
Distribution map of *Elymnias hypermnestra*.

**Figure 41.**
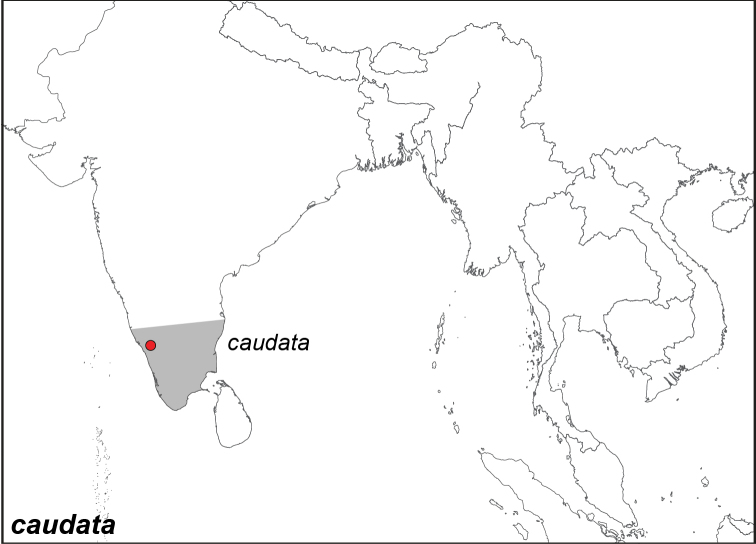
Distribution map of *Elymnias caudata*.

**Figure 42.**
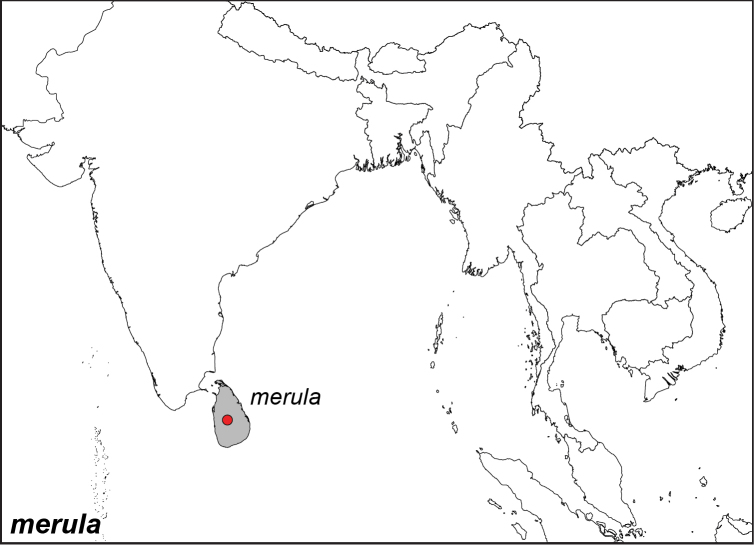
Distribution map of *Elymnias merula*.

**Figure 43.**
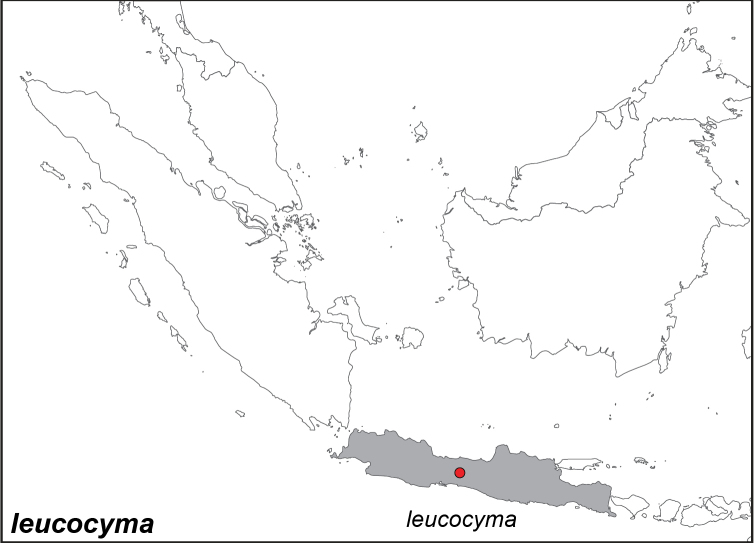
Distribution map of *Elymnias leucocyma*.

**Figure 44.**
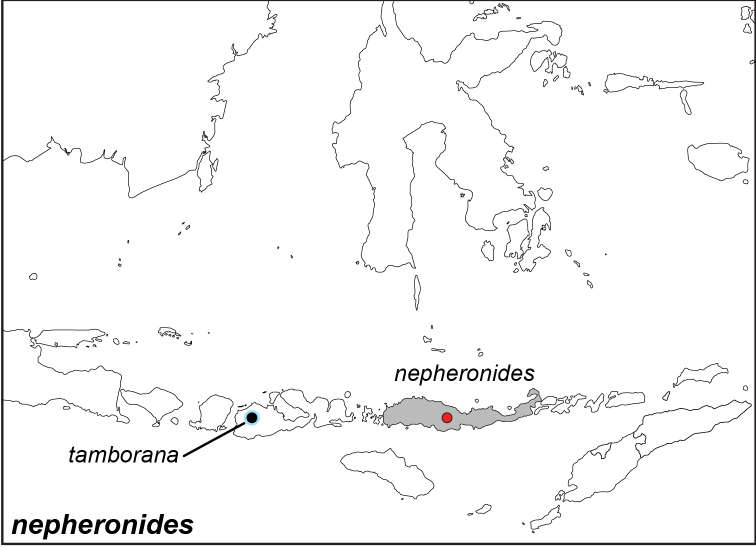
Distribution map of *Elymnias nepheronides*.

**Figure 45.**
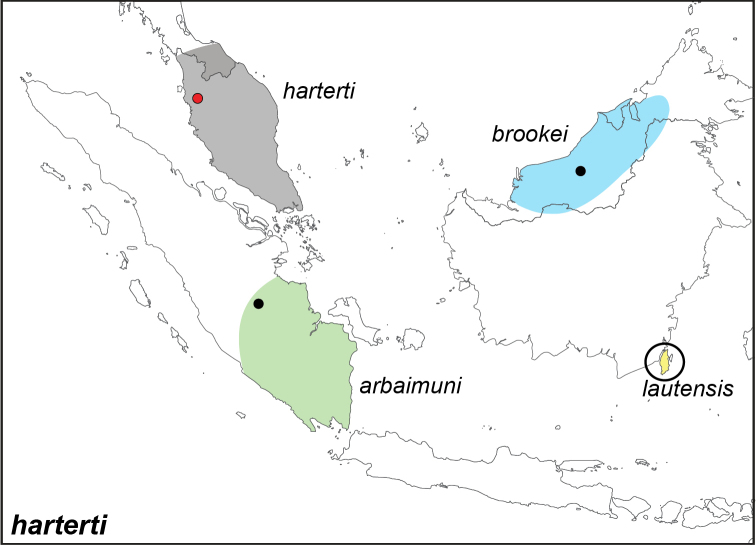
Distribution map of *Elymnias harterti*.

**Figure 46.**
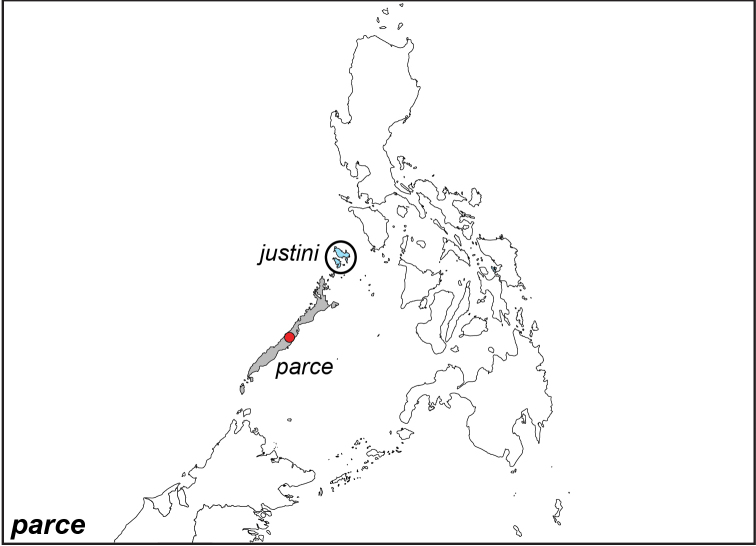
Distribution map of *Elymnias parce*.

**Figure 47.**
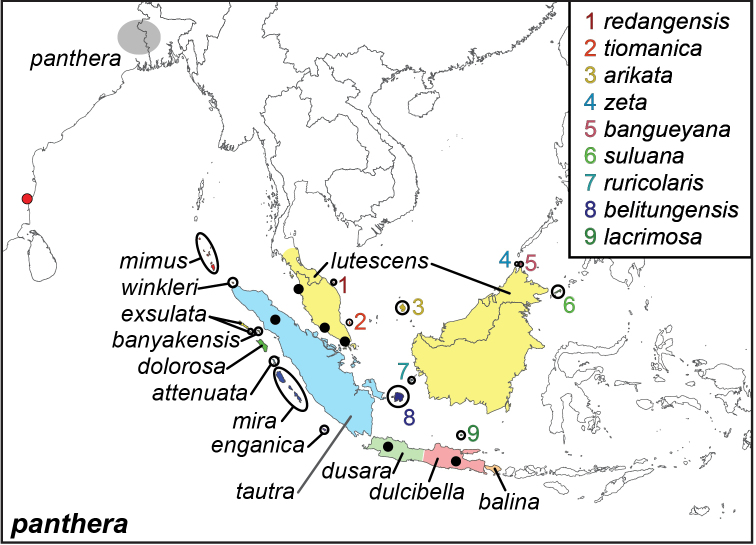
Distribution map of *Elymnias panthera*.

**Figure 48.**
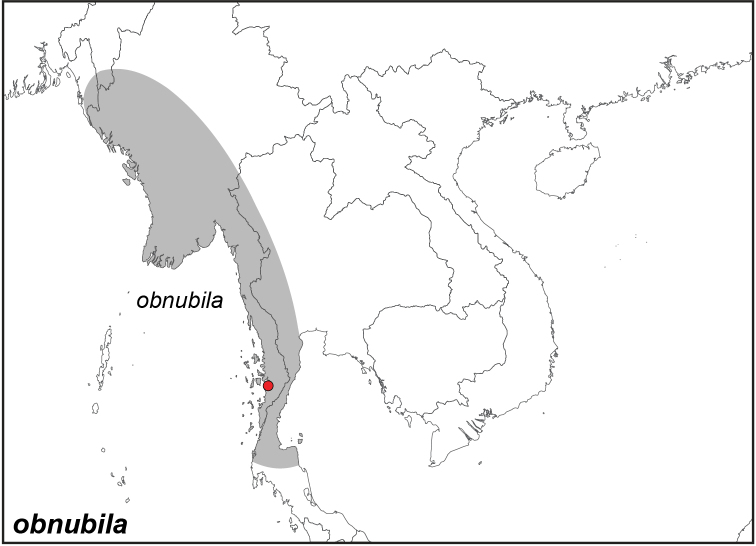
Distribution map of *Elymnias obnubila*.

**Figure 49.**
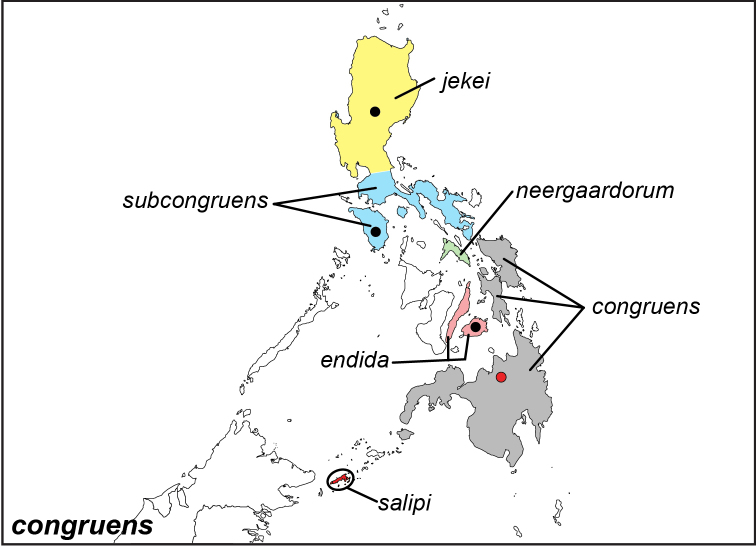
Distribution map of *Elymnias congruens*.

**Figure 50.**
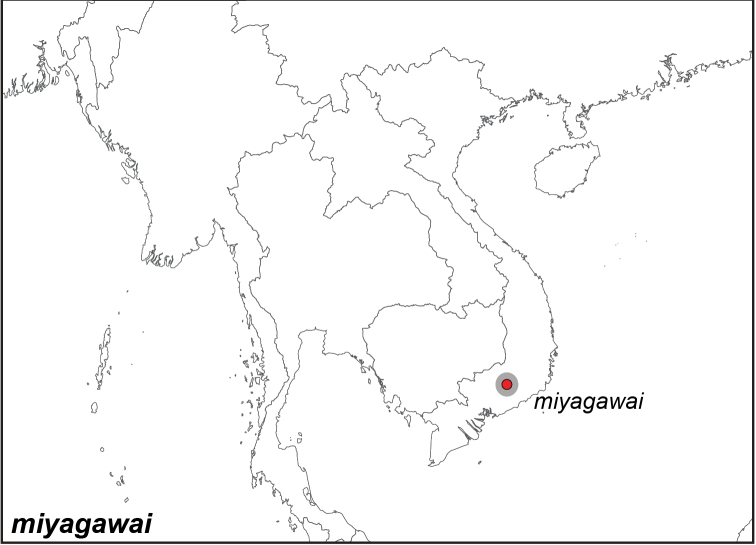
Distribution map of *Elymnias miyagawai*.

**Figure 51.**
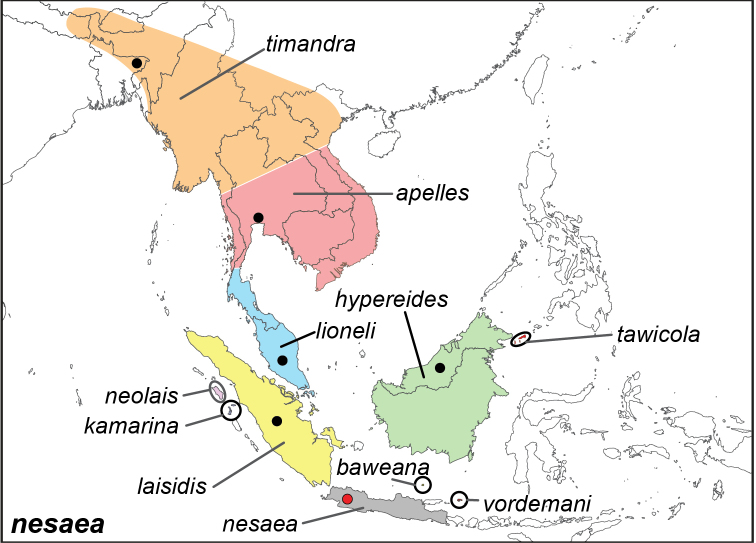
Distribution map of *Elymnias nesaea*.

**Figure 52.**
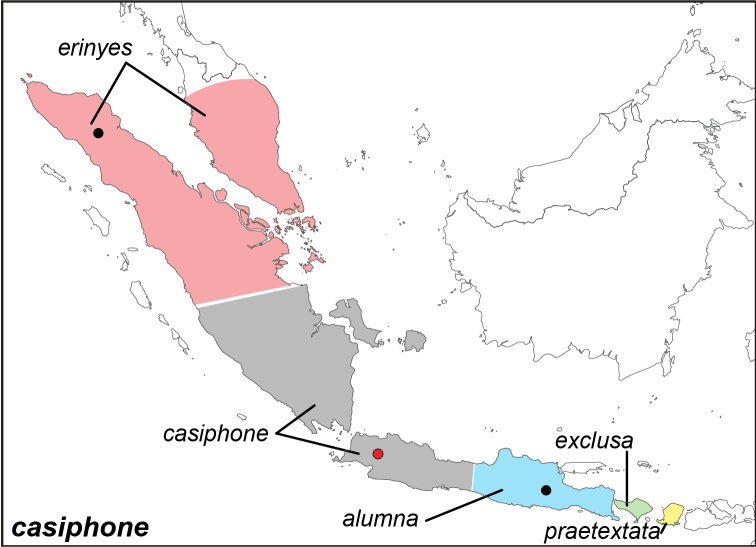
Distribution map of *Elymnias casiphone*.

**Figure 53.**
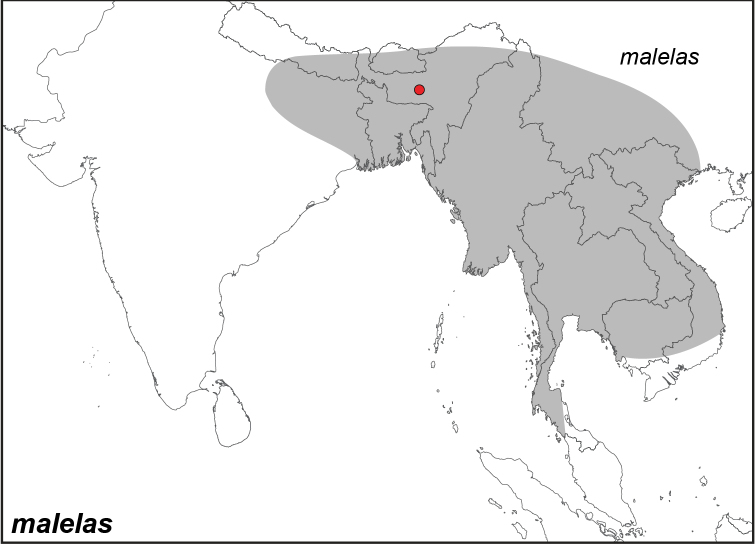
Distribution map of *Elymnias malelas*.

**Figure 54.**
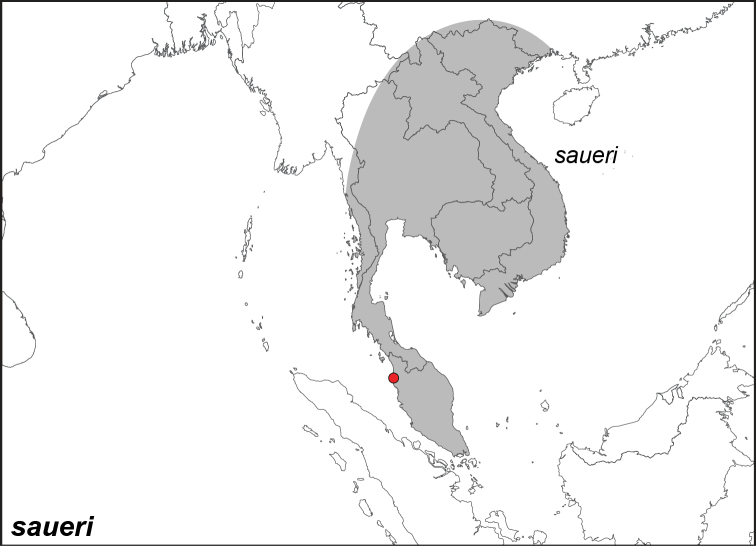
Distribution map of *Elymnias saueri*.

**Figure 55.**
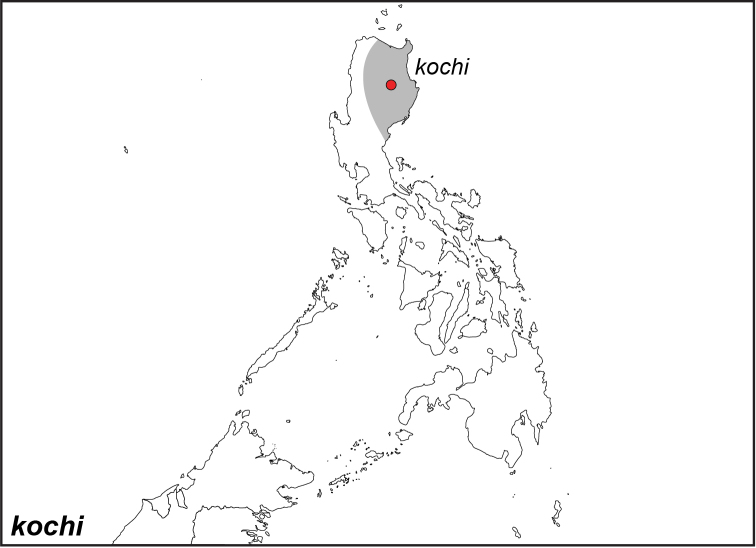
Distribution map of *Elymnias kochi*.

**Figure 56.**
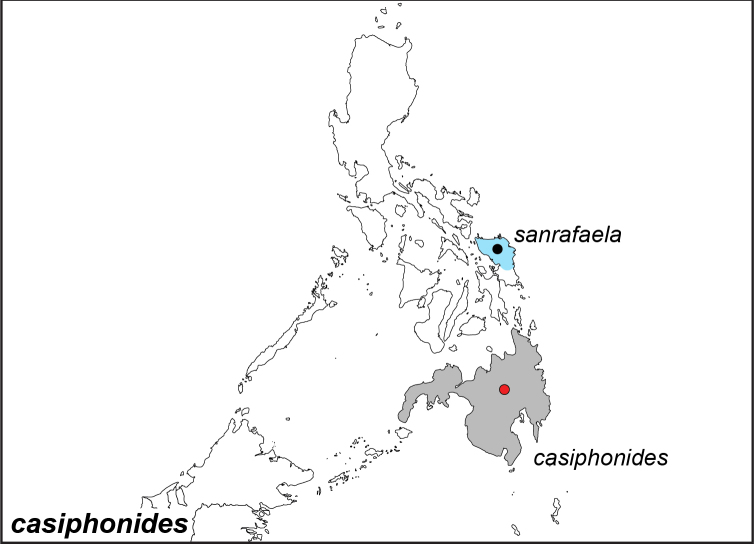
Distribution map of *Elymnias casiphonides*.

**Figure 57.**
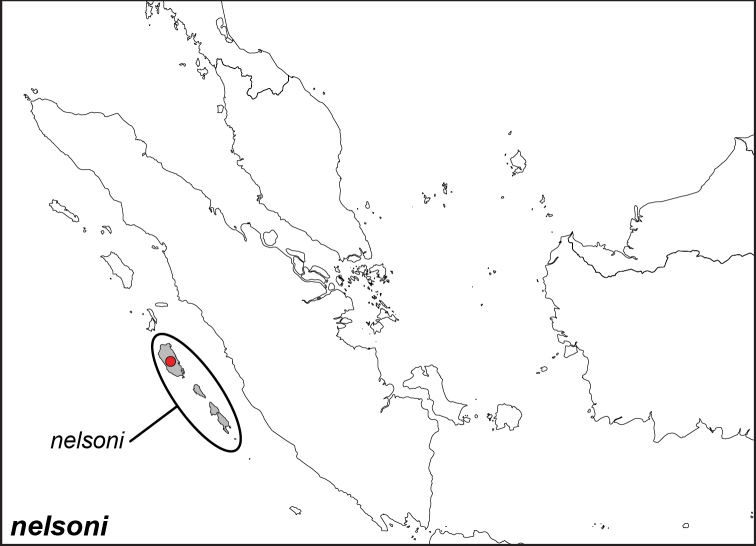
Distribution map of *Elymnias nelsoni*.

**Figure 58.**
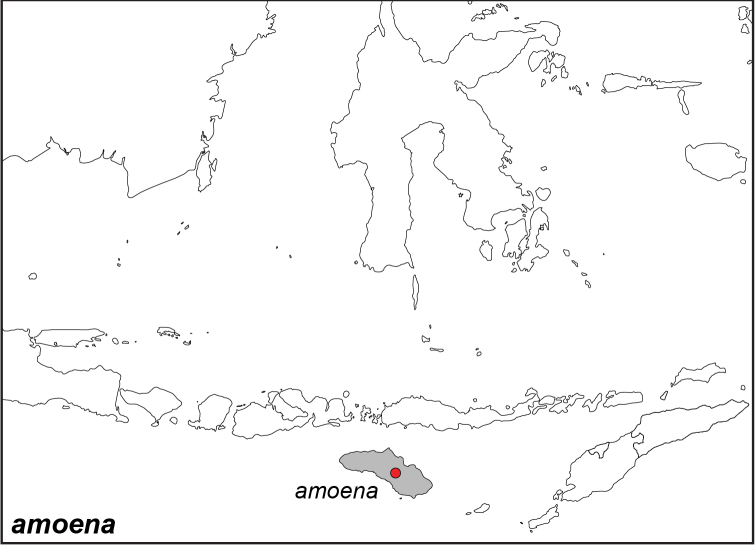
Distribution map of *Elymnias amoena*.

**Figure 59.**
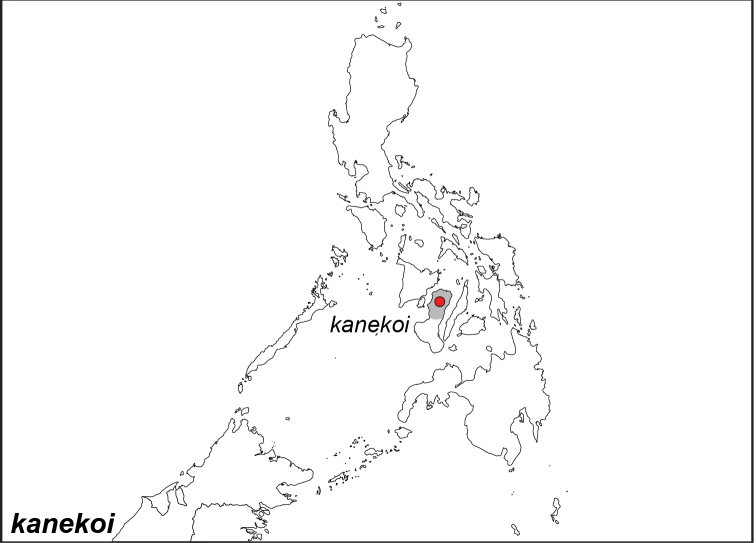
Distribution map of *Elymnias kanekoi*.

**Figure 60.**
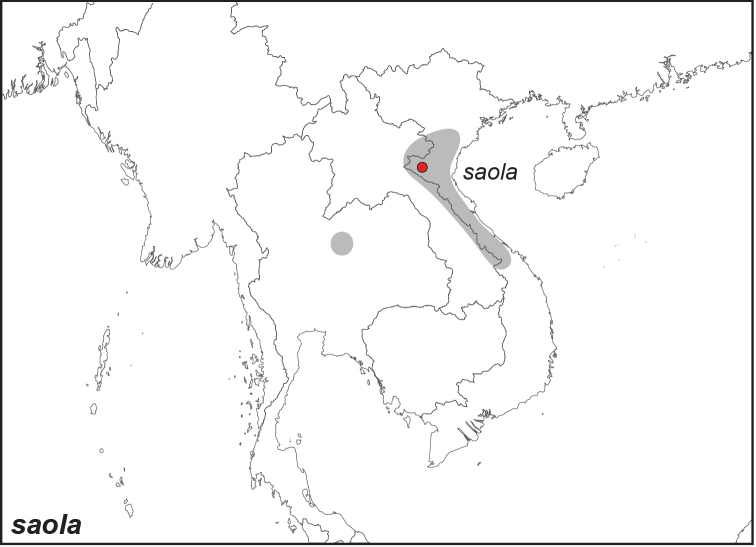
Distribution map of *Elymnias saola*.

**Figure 61.**
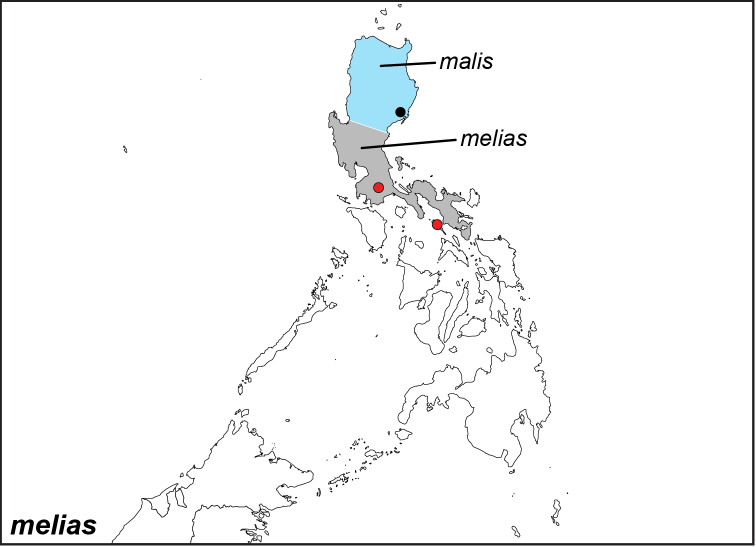
Distribution map of *Elymnias melias*.

**Figure 62.**
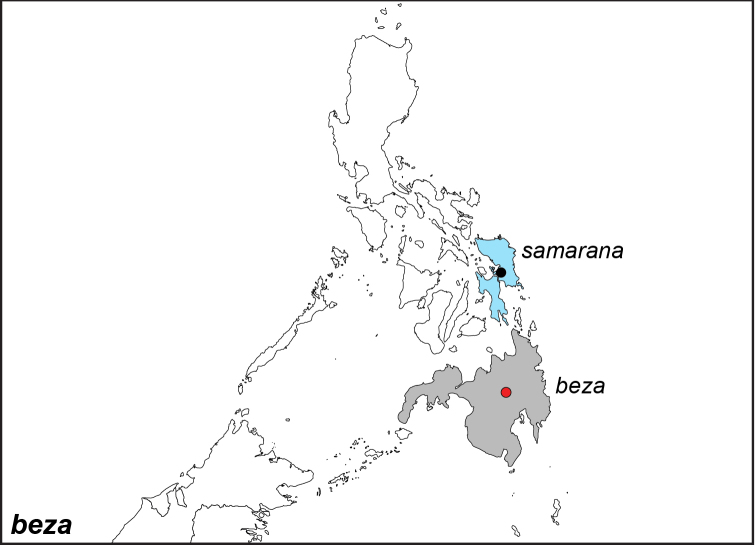
Distribution map of *Elymnias beza*.

**Figure 63.**
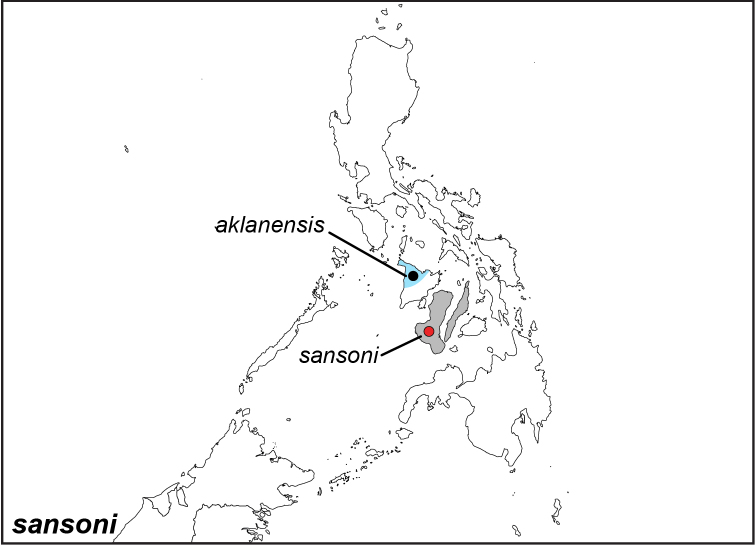
Distribution map of *Elymnias sansoni*.

**Figure 64.**
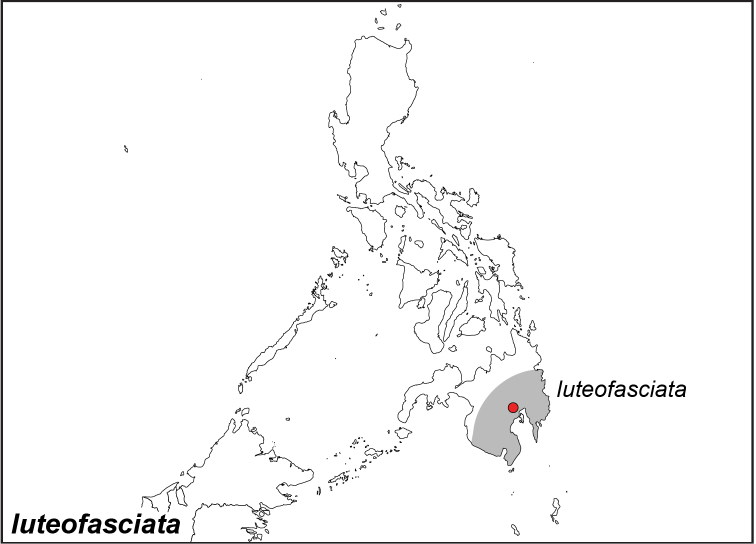
Distribution map of *Elymnias luteofasciata*.

**Figure 65.**
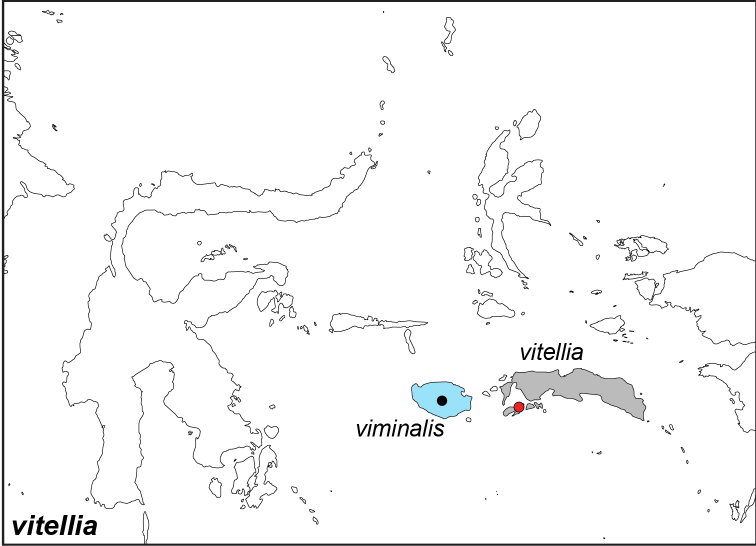
Distribution map of *Elymnias vitellia*.

**Figure 66.**
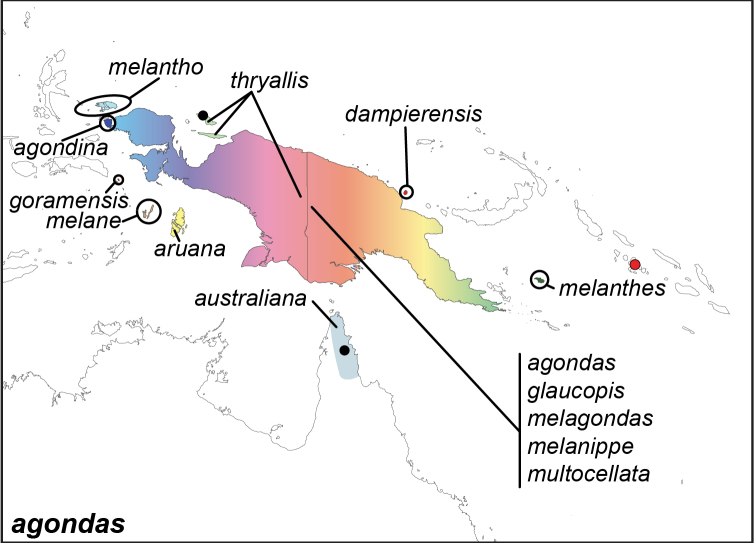
Distribution map of *Elymnias agondas*.

**Figure 67.**
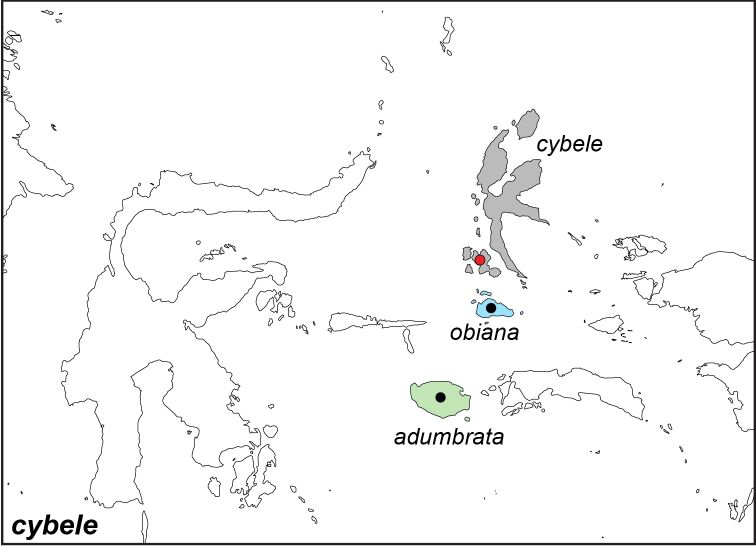
Distribution map of *Elymnias cybele*.

**Figure 68.**
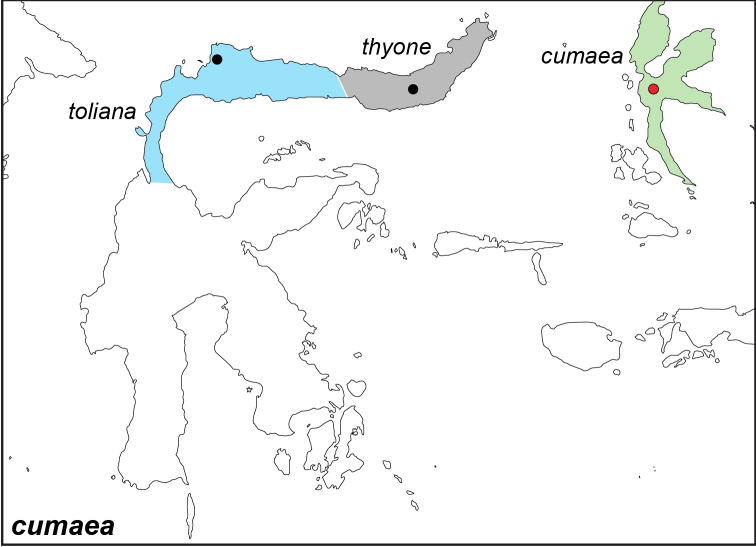
Distribution map of *Elymnias cumaea*.

**Figure 69.**
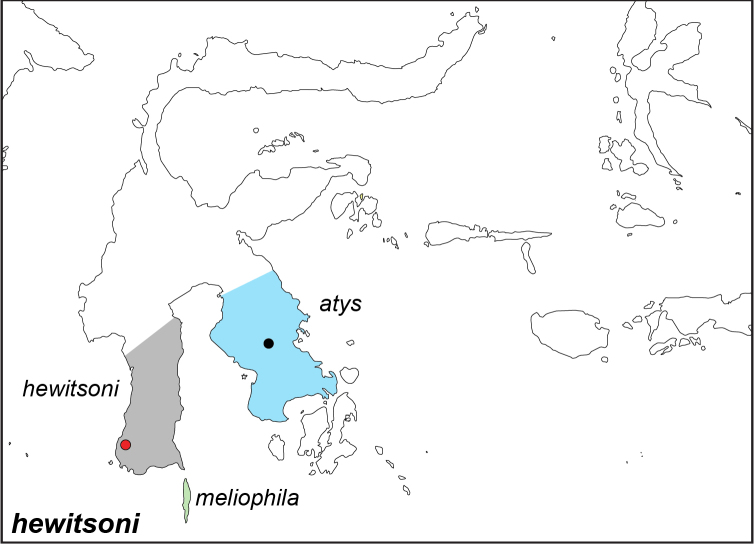
Distribution map of *Elymnias hewitsoni*.

**Figure 70.**
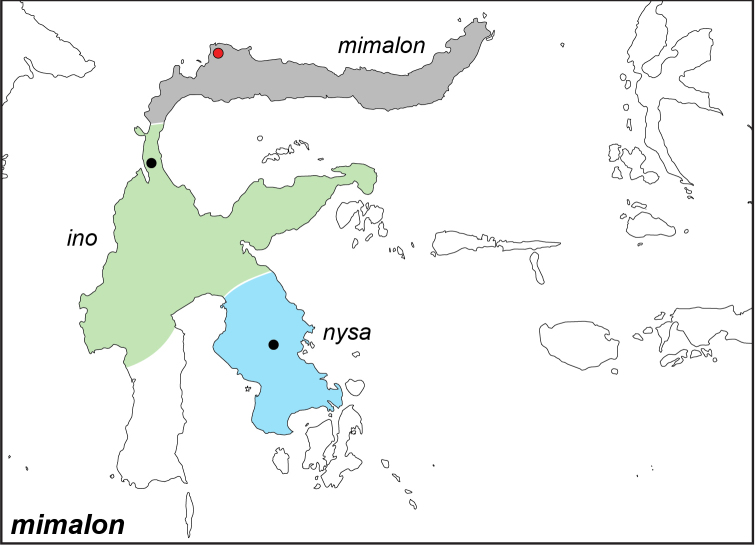
Distribution map of *Elymnias mimalon*.

**Figure 71.**
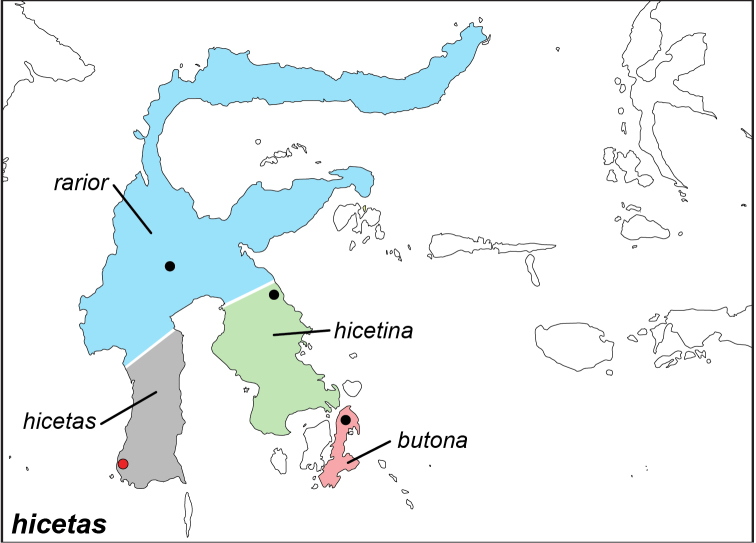
Distribution map of *Elymnias hicetas*.

**Figure 72.**
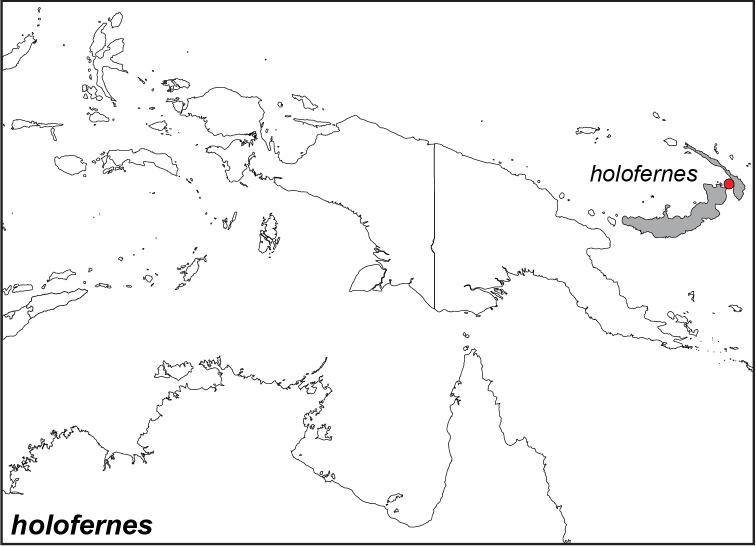
Distribution map of *Elymnias holofernes*.

**Figure 73.**
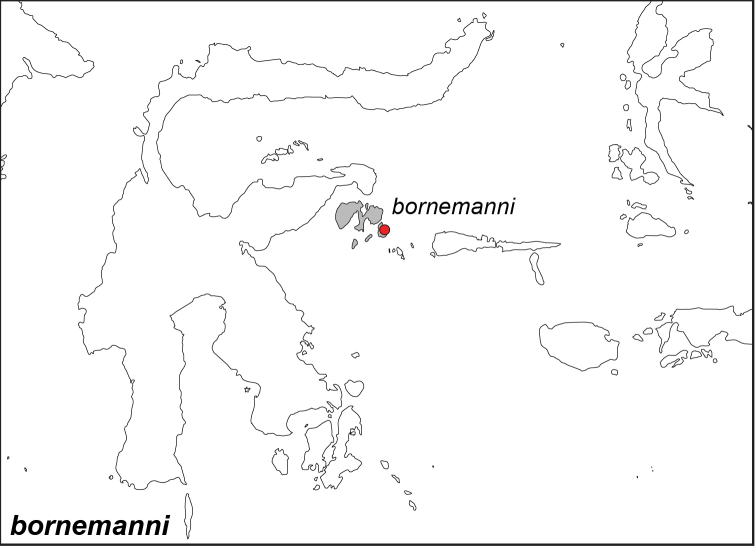
Distribution map of *Elymnias bornemanni*.

**Figure 74.**
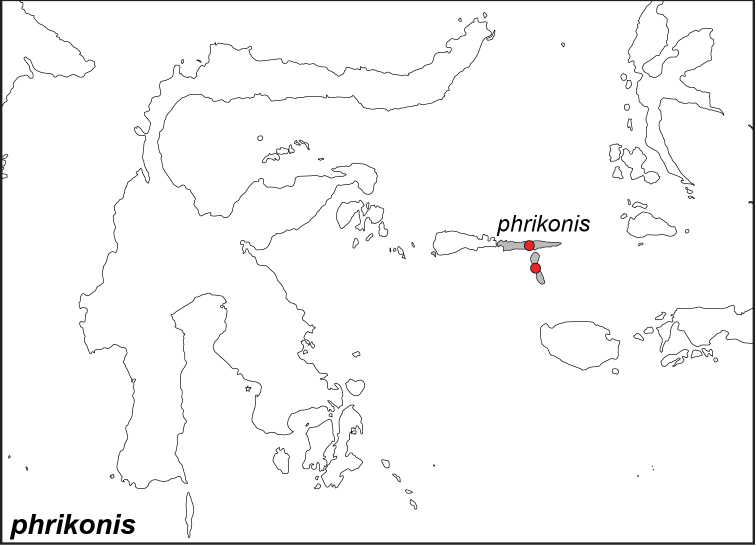
Distribution map of *Elymnias phrikonis*.

**Figure 75.**
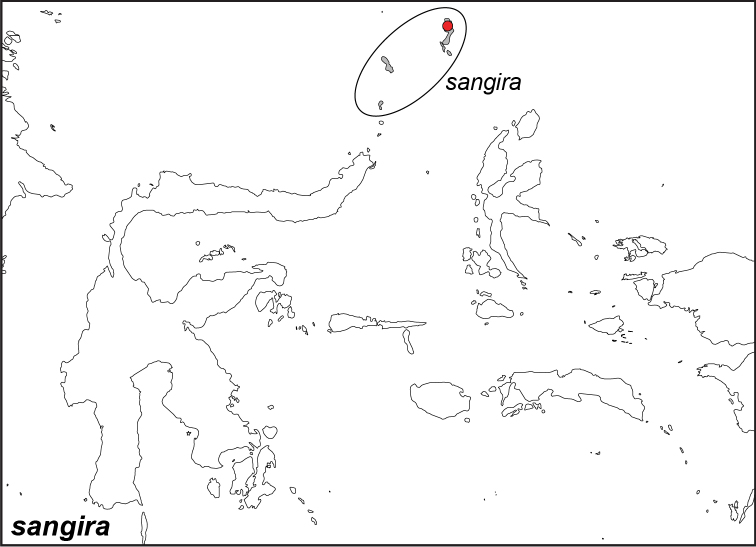
Distribution map of *Elymnias sangira*.

**Figure 76.**
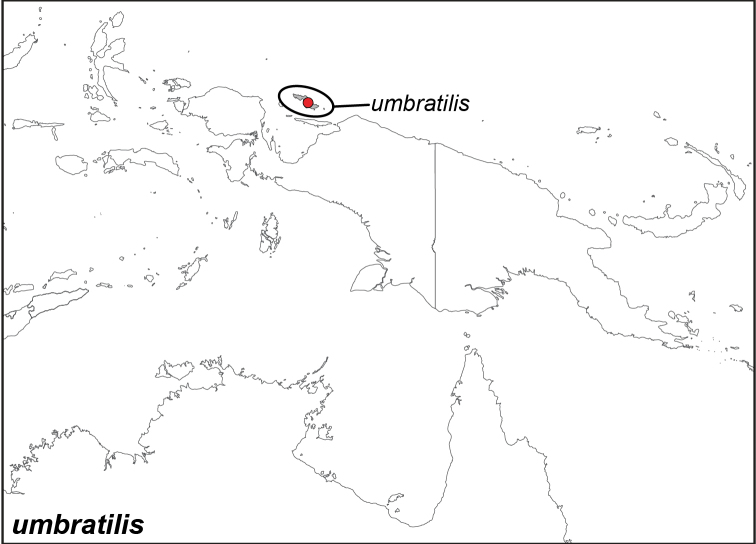
Distribution map of *Elymnias umbratilis*.

**Figure 77.**
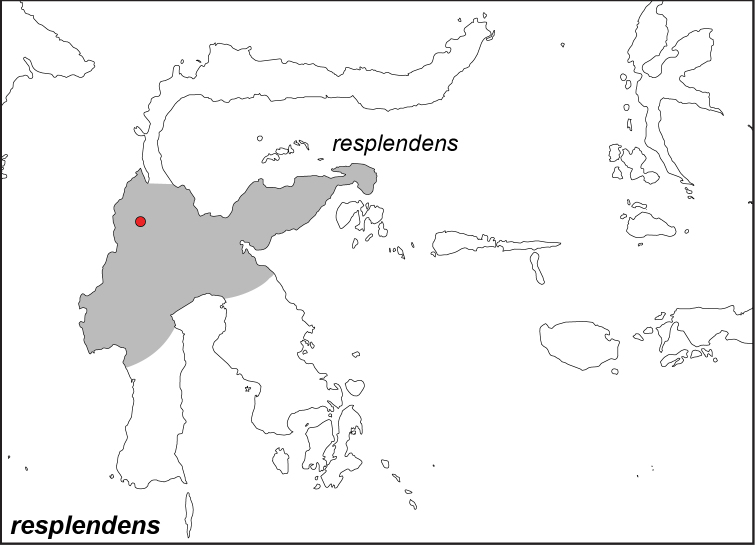
Distribution map of *Elymnias resplendens*.

**Figure 78.**
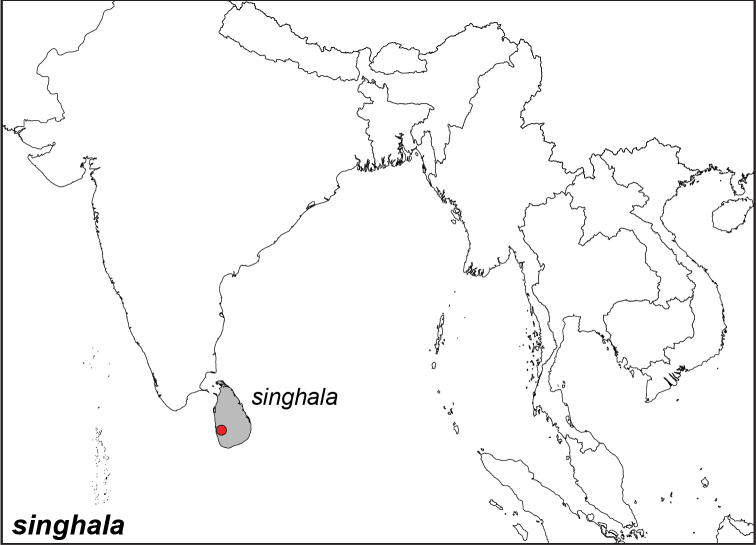
Distribution map of *Elymnias singhala*.

## Footnotes

1 The type species of *Didonis* Hübner was erroneously thought to be *Papilio biblis* Fabricius, 1807 in earlier literature. Scudder (1875) subsequently selected *Papilio vitellia* Stoll as the type species, and this designation thereby prevented misusage of the name and confusion with the genus *Biblis* Fabricius, 1807 as stated by Hemming (1967).

2 Moore (1894) did not include any species when establishing *Mimadelias* in Part 18 of his *Lepidoptera Indica*. The type species *vasudeva* was subsequently designated as the type species in his Part 19 of the same series.

3 *Elymniopsis* has often been regarded as a distinct genus since its establishment in 1907 by Fruhstorfer, and most references of Afrotropical butterflies list it as a genus of its own (*e.g.*, Larsen 2005). Hemming (1943) first synonymized this genus with *Elymnias* and the opinion was followed by Gardiner (2010) and further labelported by the phylogenetic studies by Peña et al. (2006). In our molecular phylogenetic study (Lohman et al. in prep.), this taxon is sister to all of the Asian species. However, aside from their wing patterns, which mimic various *Acraea* spp. (Nymphalidae: Heliconiinae), the morphological features of this species are not distinct from other *Elymnias* (Wei et al. in prep.), and we refrain from retaining the monotypic genus *Elymniopsis*.

4 We recognize 5 subspecies of *papua* in the present study and synonymize *bivitatta* with *papua* because no significant morphological differences were found. In addition, no apparent barriers to dispersal seem to exist between the geographic ranges of these two taxa.

5 Since Wallace only traveled to the Bird’s Head Peninsula on New Guinea, it is likely that the type specimen was collected in what is now the Indonesian province of West Papua.

6 The distinctiveness of *esaca* and *vasudeva* has never been doubted and they have been treated as distinct species in all prior studies. The former is distributed throughout most of the Greater Sunda Islands, the Philippines, and the Thai-Malay peninsula, and the range of the latter encompasses northeast India, Myanmar, northern Laos, northern Vietnam and southwest China. The wings of male *esaca* are shorter and more attenuate than *vasudeva*, and have black ground coloration with a metallic submarginal band in some specimens. The male of *vasudeva* is not dramatically different from the female in wing shape or color pattern. However, our molecular phylogenetic analysis (Lohman et al. in prep.) reveals that both specimens of *vasudeva* (from China and India) are nested within a clade of three *esaca* specimens from Java, Mindanao, and peninsular Malaysia. This paraphyletic relationship suggests that the two species should be synonymized. However, we regard both species as valid because: 1) wing color and pattern are strongly dimorphic in *esaca*, but more or less monomorphic in *vasudeva*; 2) the wing shape of *esaca* males differs from females, and this is not true of *vasudeva*; and 3) the two taxa are parapatric.

7 “Battak Mountains” is the type locality for many butterfly and other animal taxa, but the name appears on no recent map of Sumatra. It seems to refer to the mountainous region historically inhabited by the Batak ethnic groups in northeast Sumatra. From the description in de Nicéville and Martin (1895), “Battak Mountains” seems to refer to the portion of the Barisan Mountain Range running along the western edge of North Sumatra Province, including the peaks surrounding Lake Toba.

8 The subspecies *esaca taeniola* is synonymized with *esaca borneensis* because there are no consistent morphological differences between them and no obvious biogeographical barriers within the island Borneo that would restrict gene flow and maintain subspecific differences.

9 *oberthuri* was originally described as a species by Fruhstorfer (1902b) and subsequently downgraded to be a subspecies of *vasudeva* (Fruhstorfer 1907). We synonymize it with *esaca andersonii* because these two names seem to represent opposite sexes of the same subspecies confined to the Thai-Malay Peninsula.

10 Having examined all the type specimens and a long series of other material ranging from northeast India to southwest China, we failed to detect any consistent differences among the subspecies described by (Moore 1857; 1894), Wallace (1869), and Chou (2000). We consider all *vasudeva* subspecies to be indistinguishable from one another; the variable wing patterns lack diagnostic differences and are not reliable for delimiting subspecies.

11 Staudinger (1889) described *albofasciata* based on specimens from Palawan. However, the locality of a “type specimen” deposited in ZMHB is labeled as “Tanyong Malim, Malacca”. We consider the type to be either mislabeled or simply not a type of this subspecies. Staudinger (1889) indicated that he compared specimens of *dara* from Malacca (specimen provided by Künstler) and Palawan. The mistake in labeling is probably caused by historical confusion of the type locality since its publication.

12 Moore (1894) described *patnoides* as a distinct species, but Fruhstorfer (1907) downgraded it to be a form of *patna patna*. In the same publication, Fruhstorfer (1907) described *patna stictica* from Vietnamese specimens. Having examined long series from India, Myanmar, northern Thailand, northern Laos, Vietnam and Hainan (China), we find no consistent differences in wing pattern and wing shape among populations in this region. We therefore synonymize both *patnoides* and *stictica* with *patna*. The subspecies *hanitschi* from the Malay Peninsula has slightly different metallic blue sheen from the nominotypical subspecies, and molecular phylogenetic analysis reveals that the *patna* from India and *patna* from Malay Peninsula form distinct sister lineages. We therefore retain the subspecies status of *hanitschi* in the present study. Ek-Amnuay et al. (2007) described *inayoshii* based on specimens collected in Ranong and Trang Provinces in peninsular Thailand. The name, however, is not available under the Code. Additionally, the taxonomic status of the peninsular Thai populations is questionable, as we find that the wing shape and coloration of *inayoshii* are markedly different from *patna patna* and *patna hanitschi*. Since we have no material for DNA sequencing, we cannot determine whether the peninsular Thai populations should be treated as a subspecies of *patna* or a different species altogether . The nomenclatural problem of *inayoshii* requires confirmation of the taxon’s species identity.

13 Westwood (1851) was possibly unaware of the true collection locality of the type specimen of *ceryx*. Having examined the description, we confirm that the type locality of the type specimen should be Java, not Mexico.

14 Toxopeus’s *mariae* was described from Sumatra, from which de Nicéville’s *dohrnii* was also collected. Since there are no remarkable or consistent morphological differences between the subspecies and because no biogeographic or climatic barriers to dispersal seem to exist on Sumatra, we consider *mariae* to be a junior synonym of *dohrnii*. de Nicéville’s *dohrnii* was once placed as a subspecies either of *pellucida* (Fruhstorfer 1907) or of *patna* (D’Abrera 1985), here we can confirm it should be associated with *kuenstleri* after having examined the specimens from Sumatra.

15 The taxonomic status of De Nicéville’s *ceryxoides* has been inconsistent. It was originally proposed as a subspecies of *ceryx*, and the treatment was followed by Fruhstorfer (1907). Aoki et al. (1982) suggested upgrading *ceryxoides* as an independent species without giving explanation, but this taxon was again downgraded as a subspecies of *ceryx* by D’Abrera in 1985. Having examined both morphology and genetic data, we conclude that *ceryxoides* should be regarded as a full species endemic to Sumatra.

16 *Elymnias penanga* is one of the few *Elymnias* species with polymorphic female color patterns. However, the female forms are not diagnostically different among subspecies. The diagnostic characters that distinguish subspecies are the size, forewing shape, and metallic sheen of the male.

17 *Elymnias hypermnestra* is one of the few satyrine species that is regarded as a minor pest of several species of palms. The name has been used numerous times in the taxonomic, ecological, and agricultural literature (Corbet 1943; Koh and Gan 2007; Merrett 1993; Shang-Wen 1998; Yong et al. 2012). However, Lamas (2010) raised concern regarding the validity of this name. This species was originally placed in *Papilio* by Linnaeus (1763), which made it a junior primary homonym of *Papilio hypermnestra* Scopoli, 1763 (now a synonym of *Zerynthia polyxena* ([Schiffermüller], 1775) (Papilionidae). *Papilio hypermnestra* Linnaeus was published after 23 June 1763, whereas *Papilio hypermnestra* Scopoli was published before that date. Therefore, according the ICZN Article 57.2, both names are primary homonyms and the junior primary homonym (in this case, *hypermnestra* Linnaeus) is permanently invalid (see also Article 23.9.5). Given this set of circumstances, Linnaeus’ *hypermnestra* can only be regarded as valid under one of three conditions according to the code: 57.2.1. its use as a valid name (nomen protectum) is maintained under the conditions specified in Article 23.9, or 57.2.2. it is conserved by the Commission under Article 81, or 57.2.3. it, but not its senior homonym, is included in a relevant adopted Part of the List of Available Names in Zoology..” However, as already stated by Lamas (2010), *hypermnestra* Linnaeus does not fulfill any of the three conditions specified above, because: 1) *hypermnestra* Linnaeus has not been maintained as a nomen protectum and does not fulfill the conditions specified in Article 23.9; 2) *hypermnestra* Linnaeus has not been conserved by the Commission under Article 81; and 3) no part of the List of Available Names in Zoology has been adopted yet for Lepidoptera. Meanwhile, *hypermnestra* Scopoli has been used numerous times as a valid name after 1899 [see Article 23.9.1.1], and therefore does not qualify as a nomen oblitum. Consequently, if use of the younger homonym [*hypermnestra* Linnaeus] is to be maintained, the case needs to be submitted to the International Commission for Zoological Nomenclature for a ruling under the plenary power (Article 81). In addition, if Linnaeus’s *hypermnestra* is eventually considered invalid by the Commission, the other earliest available name for this species is *Papilio undularis* Drury, 1773, which is now used to represent the subspecies of northeast India, and the valid subspecific name for the population of Java would be *protogenia* Cramer, 1779.

18 Roepke’s *atrata* has rarely been mentioned in previous literature, and, having examined the original description, we regard it as a junior synonym of the nominotypical subspecies, which is also from Java.

19 Interpretation of the type locality of Drury’s *undularis* has been problematic. Drury did not clearly indicate the origin of the specimen he saw but labeled the locality as “East Indies”. During the late 18 ^th^ century, the phrase “East Indies” referred to a wide range of possible localities from India to Indonesia. However, authors working after Drury, without clear reason, interpreted *undularis* as a taxon distributed in northeast India rather than Indonesia. We examined many specimens from north India to Java at different museums and also compared the original drawings of *hypermnestra* (and its junior synonyms) and *undularis* in the literature (*e.g*., Cramer 1779; Drury 1773), but failed to detect any differences between them. We therefore retain the current concept of *undularis* until more evidence becomes available.

20 Hewitson’s *cottonis* was described as a full species due to the lack of metallic sheen or any other notable markings on the upper side of both the fore- and hindwings; its conspecificity with *hypermnestra* has not previously been suggested. Our unpublished data (Wei et al. in prep.) demonstrates that *cottonis* and *hypermnestra* cannot be distinguished using morphological characters unrelated to possible mimicry, and the molecular phylogeny reveals that cottonis is nested within *hypermnestra* with strong labelport (Lohman et al. in prep.). We therefore combine *cottonis* (including subspecies *jennifferae* from Little Andaman) with *hypermnestra*, retaining the names *cottonis* and *jennifferae* as distinct subspecies.

21 Besides *undularis* from northeast India, there are three other subspecies with orange, *Danaus*-mimicking females distributed in Myanmar, Thailand, and Vietnam. The differences between females of the subspecies *tinctoria*, *violetta*, and *meridionalis*, are subtle. Besides the female form mimicking *Danaus chrysippus* (or *D. genutia*), there is another female form with whitish hindwings (the forms *obfuscata* and *paraleuca*) that possibly mimics either *D. melanippus* or *D. affinis* in Thailand and Vietnam. Since the current subspecies classification has been adopted by local guidebooks and other publications for so long (Corbet et al. 1992; Ek-Amnuay 2012; Monastyrskii 2005; Pinratana 1988), we do not propose any nomenclatural change prior to a thorough phylogenetic/population genetics study based dense sampling of the entire region is completed.

22 Moore’s *hainana* was described from specimens from Hainan Island, China, and the name has been applied to the Taiwanese population since the late 19 ^th^ century. Since Hainan is between China’s Guangxi Province and northern Vietnam, where *septentrionalis* and *tonkiniana* were described, respectively, and because examination of dozens of specimens evince no consistent morphological differences among these subspecies, we synonymize these three names and regard *hainana* as the valid name.

23 Penang is a small island in the Andaman Sea lying just off the western coast of peninsular Malaysia. The strait that separates this small island (293 km ^2^) from the peninsula is only 2–8 km wide, yet seems to form a dispersal barrier between the peninsular population (commonly known as *agina*, but herein changed to *beatrice*, see discussion below) and insular *discrepans*. The female type specimen of *discrepans* seems to be a morphologically intermediate form between orange, *Danaus*-mimicking *tinctoria* and dark, *Euploea*-mimicking phenotypes. This phenotype has not been documented from the mainland. We therefore retain the name *discrepans* because of the taxon’s distinctive female wing patterns; further studies will ascertain whether this subspecies is genetically distinct from other *hypermnestra* subspecies.

24 *Elymnias hypermnestra* from Malay Peninsula, Singapore, Borneo, and Sumatra have been historically confusing and chaotic. In 1882, Distant reported and described *nigrescens* from the Malay Peninsula, Malacca, Billiton (Belitung), and Borneo. Since Butler (1871) already stated that the type locality of the “real *nigrescens*” is Sarawak (Borneo), and the female wing patterns of the populations in Borneo and Malay Peninsula are slightly different, it is not appropriate to apply the name *nigrescens* to the peninsular population. Fruhstorfer (1902) noticed that “ *nigrescens*” *sensu* Distant was different from the Bornean one, so he proposed a nomen novum, *beatrice*, to refer to “ *nigrescens**sensu* Distant”. The concept of Fruhstorfer’s *beatrice*, however, is probably not monophyletic as he listed Perak, Lingga (Riau), Deli (North Sumatra), and Sumatra in the geographical range of *beatrice*, but our morphological study does not labelport lumping the Sumatran population with the peninsular Malaysian one. In the same publication, Fruhstorfer (1902a) described an aberration of *nigrescens*, namely *agina*, for populations in Singapore, Sumatra, and Perak. The name *agina* has been used much more frequently than *beatrice* to represent the population in the Malay Peninsula including Singapore (Corbet 1943; Lamas 2010; Pinratana 1988). However, Article 45.6.2 of the Code, *agina* was not an available name when Fruhstorfer first proposed it as an aberrant form of *nigrescens* (now a valid subspecies of *hypermnestra*). This name might have subsequently become available by Corbet (1943) when he discussed the taxonomy of the *Elymnias hypermnestra* of Malay Peninsula, and first used *agina* to represent the populations in Johor and Singapore. According to the Code (Article 45.5.1), Corbet would be the first author to make *agina* available so in the present study, we correct the authorship and year of *agina* to be “Corbet, 1943”. Both *beatrice* and *agina* became available for representing the populations in Malay Peninsula and Singapore. Determination of their validity, therefore, depends on the priority. Considering the fact that Corbet’s (1943) use of *agina* is much later than Fruhstorfer’s (1902a) proposal of a nomen novum, we conclude that *beatrice* should be used to represent the populations in southern part of the Malay Peninsula, including Singapore. The syntype series of both names contain more than one subspecies, so designation of a lectotype for both names will be necessary to fix the concept and use the names. This work will be published elsewhere.

25 de Nicéville and Martin (1895) stated that they had “great difficulty in identifying satisfactorily the common species of *Elymnias* of the *undularis* group occurring in Sumatra”. They decided to follow Distant’s (1882a) concept of “ *nigrescens*” but still noticed that the Sumatran population of *Elymnias hypermnestra* (as *nigrescens* or *protogenia*) had smaller wings and duller coloration. Fruhstorfer (1907) noticed the opinion of de Nicéville & Martin, and decided to give the Sumatran population a status as a color form, and name it *decolorata*. However, since it was originally published as an infrasubspecific taxon, the name is not available under the Code unless another author uses the name to represent a valid taxon. In 1982, Aoki and colleagues enumerated the subspecies of *Elymnias hypermnestra* that occur throughout its range. They became the first authors to use *decolorata* to represent the Sumatran population. According to the Code (Article 45.5.1), the authorship of *decolorata* should be attributed to Aoki et al. (1982) because they made it available for use for the first time.

26 Although *caudata* was originally proposed as a species of its own, some authors (Gupta 2007; Wynter-Blyth 1957) treated it as a subspecies of *hypermnestra* (or *undularis*) due to the similarity in the *Danaus*-mimicking females. Our morphological and molecular studies demonstrate that *caudata* is a distinct, monophyletic taxon that is sister to *hypermnestra*.

27 Swinhoe’s *merula* is based on a single male type collected from Sri Lanka. Having examined the type deposited in the Natural History Museum, London, we are convinced that *merula* should be a synonym of *hypermnestra*. Lamas (pers. comm.) suggests synonymizing *merula* with the Sri Lankan *fraterna*; however, the male of Sri Lankan *fraterna* is quite different from *merula* and we cannot at present conclude that synonymizing it with this subspecies is warranted. We presume that the single specimen of *merula* was accidentally introduced with imported palms or is an aberration; we treat this name as *incertae sedis*.

28 The true identity of Godart’s *leucocyma* has been problematic since its description in 1819. Godart specified “Java” as the source of the specimen but gave a vague description without any figure. Doubleday (1844) suggested that northern India (near the border with Myanmar) might be the source of the specimen. Moore (1878a) mentioned the name *leucocyma* in his checklist without providing any further information. In 1882, Marshall & de Nicéville recognized the validity of *leucocyma* and synonymized *malelas* with it. However, Moore (1894) considered *leucocyma* to be the name that should be validated rather *malelas*. Fruhstorfer (1907), based on Godart’s simple description, doubted that the origin of specimen was Java or northern India, and suggested placing *leucocyma* closer to *hypermnestra*. There are more than 2 species with color patterns similar to *leucocyma* (viz. forewing with metallic blue sheen and hindwing with undulate margin) in Java and northern India, so we cannot specify the use of this name until more evidence becomes available. Moreover, Hewitson’s figure (1861: pl. 9, fig. 34) of *leucocyma* was a misidentification of *hewitsoni* and has no relevance to this problem.

29 Our molecular phylogenetic analysis confirms that *detanii* and *nepheronides* represent opposite sexes of the same species as Araya (2016) demonstrated using morphology. We also accept Araya’s (2016) decision to include *tamborana* as a subspecies of *nepheronides* on morphological grounds; we currently have no genetic data from *tamborana*.

30 Having examined specimens deposited in the NHM, we are confident that *harterti* and *brookei* should be regarded as different subspecies of the same species. Moulton’s *smithi* is identical to Shelford’s *brookei* so they are synonymized in the present study.

31 Staudinger (1889) placed *parce* as a subspecies of *panthera*, but morphological and molecular evidence suggest that *parce* does not belong to the *panthera*-group; it is more closely allied to *harterti*.

32 Fabricius stated that the type locality of the nominotypical *panthera* is Tranquebar (Tharangambadi, Tamil Nadu) in southern India, but the current distribution of this species in India seems to be restricted to the north. It is necessary to confirm the actual distribution of the species in India to verify whether the type locality falls in the actual distribution range or is simply a port from which the specimen was exported during the colonial period.

33 The subspecies *dusara* and *dulcibella* were both described from Java, suggesting the names might be synonymous. However, many lepidopteran species have distinctive populations western and eastern parts of the island (Aoki et al. 1982; Tsukada and Nishiyama 1982; Yata and Morishita 1985), and we therefore retain these two subspecies as valid.

34 Butler’s *lutescens* was proposed as a full species based on specimens from Borneo (collected by Lowe), Malacca, Singapore, and Penang (from Roberts’ collection), and according to Butler (1867: 404), *lutescens* was similar to *dusara*. This taxon, however, has been synonymized with the Indian nominotypical subspecies for long with no clear reason. In the present study, we revalidate the name and use it to represent the population in Borneo, as Borneo is the first locality mentioned in Butler’s original description. The other conspecific taxa described from Borneo, such as *labuana*, *lacrima*, *defasciata*, *alfredi*, and *pantherina* are therefore newly synonymized with *lutescens* in the present study. It is not clear whether *panthera* from peninsular Malaysia is genetically distinct from Bornean populations, so we do not further revise the plethora of subspecific names associated with *panthera*.

36 Linnaeus did not indicate from which part of Java his specimen of *nesaea* was collected, and we found no consistent differences among Javan *nesaea* populations based on examination of specimens at several different museums, so we combine Fruhstorfer’s *hermia* with the nominotypical *nesaea*.

37 Wallace’s *timandra* from Sylhet, Bangladesh, is not different from Fruhstorfer’s *cortona* from Myanmar, so we synonymize them.

38 We examined many *nesaea* from different regions of Borneo and found no consistent difference among them. Frustorfer’s *coelifrons* is therefore treated as a junior synonym of *hypereides*. The relationship between the Malayan *lioneli* with *hypereides* can be addressed in future phylogenetic studies.

39 Lamas (pers. comm.) regards *casiphone* and *kamara* as different species, so *erinyes*, *exclusa*, and *lombokiana* are therefore regarded as subspecies of *kamara*. Our phylogenetic study based on morphology and DNA sequence data, however, show that *kamara* is conspecific with *casiphone*; *kamara* seems to represent a sexually dimorphic, mimetic form in which both males and females differ from the sexually dimorphic mimetic forms of *casiphone*. We therefore associate all subspecies previously included under *kamara* with *casiphone*.

40 Geyer, when describing *casiphone*, did not specify the geographical provenance of his specimen. Westwood (1851) and Fruhstorfer (1907) suspected that Java was possibly the origin of Geyer’s *casiphone*. Having compared the original drawing of *casiphone* and the specimens from Java, Sumatra, Bali and Lombok, we conclude that the color pattern of the Javanese population matches well with Geyer’s figure.

41 de Nicéville’s *erinyes* was originally described as a full species, and then downgraded to be a subspecies of *casiphone* by Fruhstorfer (1907) or *kamara* (Corbet et al. 1992). Since we now consider this subspecies should be associated with *casiphone*, Fruhstorfer’s combination should be revived.

42 Since we regard *kamara* as a junior synonym of *casiphone*, the name *lombokiana*, originally described as a subspecies of *kamara*, is treated as a new junior synonym of *praetextata* in the present study.

43 Martin’s *djilantik* and de Nicéville’s *exclusa* were previously placed under *casiphone* and *kamara*, respectively. We regard them as different color forms.

44 We examined many specimens from northeast India to Vietnam and found no consistent morphological differences to labelport the current subspecies classification. We therefore synonymize *ivena* and *nilamba* with *malelas*.

45 Distant’s *saueri* was originally described as a full species and then placed under *casiphone* as a subspecies by Fruhstorfer (1907) due to the similarity in wing pattern. Recently, Araya and Saito (2014) separated them into two morphologically defined species. Our phylogenetic analysis reveals that these taxa are not even sister to each other. The sister species of *saueri* is *saola*, and we therefore affirm the species status of *saueri*.

46 Semper (1887) described *kochi* as a full species, while Fruhstorfer downgraded it to be a subspecies of *beza* due to the similarity of their wing patterns. Treadaway and Schroeder (2012) considered *kochi* a full species, and our phylogenetic study places *kochi* as the sister group of *kanekoi* from Negros, so its status as a full species is upheld.

47 The physiognomy of *casiphonides* is remarkably similar to female *casiphone* and female *malelas*, however, our molecular phylogenetic study places it as the sister group of *nesaea*. The similarity is almost certainly the result of these different lineages mimicking the same widespread model: female *Euploea mulciber*.

48 According to the current taxonomy, two subspecies of *melias* are recognized and distributed in Luzon, and it seems unusual for a single island to harbor more than one subspecies. The biotic regions of Luzon, however, are complex. The northern Sierra Madre mountains may serve as a geographical barrier within the island as suggested by Vallejo (2014).

49 Fruhstorfer (1907) placed *plateni* as a subspecies of *kochi*, but our molecular phylogenetic analysis reveals that *kochi* is the sister species of *kanekoi*. Therefore, *plateni* should be synonymized with the nominotypical *beza*, which is a member of the *melias* group.

50 The species *agondas* has long been considered a highly variable species, the females of which mimic various *Taenaris* spp. throughout its range in New Guinea and the surrounding islands. Our phylogenetic analysis, however, reveals that *thryallis*, which was currently placed as a subspecies of *cybele*, is nested within *agondas*, and the branch labelport for this relationship is strong. We therefore conclude that both *agondas* and *cybele* are not monophyletic species as currently circumscribed. We sink *thryallis* into *agondas*, thus forming a monophyletic species, but further clarification of the subspecific nomenclature is difficult because of the myriad described taxa, vague descriptions of many type localities (frequently “New Guinea”), and our lack of access to material of several rare “subspecies” of *agondas*, *cybele*, and other members of the species group.

51 The true identity of *agondas* is mysterious. Boisduval stated that the source of the type specimen was the Solomon Islands (Boisduval 1832: 138, pl. 3, fig. 5). However, no *Elymnias* are known from the Solomon Islands at present (Tennent 2002), and the easternmost point in the range of this species is Woodlark Island in Milne Bay Province, Papua New Guinea—approximately 350 km from the Solomon Islands across the Solomon Sea. Although the circumscription of “Solomon Islands” has changed throughout history, an area with that name has never included Woodlark Island. Bougainville Island can be included in the Solomon Islands, but *Elymnias agondas* has never been found there. The original drawing of *agondas* is a male, and since the highly variable wing pattern of male *agondas* is not a reliable diagnostic character, we tentatively retain the nominotypical *agondas* as a taxon with questionable geographical provenance, but the nominal subspecies should not be applied to any population until the true collection locality of the type can be discerned.

52 Our phylogenetic analysis shows that *cybele cybele* from Bacan is nested within *cybele opaca* from Halmahera. Morphological examination failed to distinguish these two subspecies; we therefore synonymize them.

53 The subspecies *adumbrata* was described from Buru, but it was synonymized with the nominotypical *cybele* by previous authors. Since the wing pattern of *adumbrata* is different from that of *cybele*, and Buru island is presently ~280 km from Halmahera, we revive this subspecies from synonymy with *cybele cybele*.

54 According to our phylogenetic analysis, *cumaea* is not a monophyletic group and is part of a radiation on Sulawesi that includes *hicetas* and *hewitsoni*. The subspecies *phrikonis* is not allied with other *cumaea* subspecies, but comprises the sister group to a clade including *cybele*, *vitellia*, *holofernes* and *agondas*. We therefore elevate *phrikonis* to the species level. Fruhstorfer (1907) described *relicina* from Sanana (Sula Besi), and we consider this name should be synonymized with *phrikonis* as they are not different from each other morphologically.

55 Nominotypical *cumaea* is only reported from Halmahera. We have not been able to obtain specimens for inclusion in the molecular phylogeny so we have no indication of how many subspecies previously associated with *cumaea* should be retained.

56 Having examined specimens of *thyone*, we conclude that this taxon should not be regarded as a junior synonym of *cumaea cumaea*, but we tentatively place *thyone* as a subspecies of *cumaea* pending molecular data for inclusion in a phylogenetic study.

57 In our phylogenetic study, a specimen from North Sulawesi identified as *toliana* is sister to *hicetas*, and this pair is sister to *bornemanni*, which we regard as a full species. Since we have no genetic data from nominotypical *cumaea*, we are unsure were to place this taxon and tentatively retain *toliana* as a subspecies of *cumaea*, even though this arrangement makes *cumaea* polyphyletic.

58 Having examined the type specimens of *bonthainensis* and *hicetas*, we failed to find any distinguishing morphological characters. These two names are therefore synonymized.

59 Martin described *rarior* as a subspecies of *hicetas* without a clear indication of its type locality in Sulawesi. In our phylogenetic study, two specimens identified as *rarior* are paraphyletic with regard to *hicetas* and *butona*. Since the type locality of *rarior* is unclear, we retain *rarior* as a subspecies of *hicetas*.

60 The situation of *bornemanni* is similar to that of *phrikonis*. It is not closely related to other *cumaea* subspecies, so we treat it as a full species.

61 *phrikonis* has been regarded as a subspecies of *cumaea*. Our phylogenetic analysis, however, places it as the sister to a clade consisting of *cybele*, *holofernes*, *umbratilis*, *vitellia*, and *agondas*. We therefore elevate *phrikonis* to full species status. Since *relicina* was described from the same locality as *phrikonis* we synonymize these two names in the present study.

62 *Elymnias cybele umbratilis* was originally described based on five syntypes from Biak and synonymized with *thryallis* by subsequent authors. Our phylogenetic analysis, however, reveals that *umbratilis* is a distinct taxon, not closely related to any subspecies of *cybele*, and sister to *holofernes*. We therefore revive this taxon and give it full species status.

63 Martin (1929) placed *resplendens* with *cumaea*, but our analysis suggests that *resplendens* is closely related to *hewitsoni* and deserves full species status.

64 We have been unable to obtain DNA sequence data from our tissue samples of *singhala* from old museum specimens. This species is probably not closely related to either the *hypermnestra* or *harterti* species group, but its true species group affinity cannot be deduced at this time.
